# Amides are excellent mimics of phosphate internucleoside linkages and are well tolerated in short interfering RNAs

**DOI:** 10.1093/nar/gku235

**Published:** 2014-05-09

**Authors:** Daniel Mutisya, Chelliah Selvam, Benjamin D. Lunstad, Pradeep S. Pallan, Amanda Haas, Devin Leake, Martin Egli, Eriks Rozners

**Affiliations:** 1 Department of Chemistry, Binghamton University, The State University of New York, Binghamton, NY 13902, USA; 2 Global Research and Development in Molecular Biology, Thermo Fisher Scientific Bioscience Division, Lafayette, CO 80026, USA; 3 Department of Biochemistry, School of Medicine, Vanderbilt University, Nashville, TN 37232, USA

## Abstract

RNA interference (RNAi) has become an important tool in functional genomics and has an intriguing therapeutic potential. However, the current design of short interfering RNAs (siRNAs) is not optimal for *in vivo* applications. Non-ionic phosphate backbone modifications may have the potential to improve the properties of siRNAs, but are little explored in RNAi technologies. Using X-ray crystallography and RNAi activity assays, the present study demonstrates that 3′-CH_2_-CO-NH-5′ amides are excellent replacements for phosphodiester internucleoside linkages in RNA. The crystal structure shows that amide-modified RNA forms a typical A-form duplex. The amide carbonyl group points into the major groove and assumes an orientation that is similar to the P–OP2 bond in the phosphate linkage. Amide linkages are well hydrated by tandem waters linking the carbonyl group and adjacent phosphate oxygens. Amides are tolerated at internal positions of both the guide and passenger strand of siRNAs and may increase the silencing activity when placed near the 5′-end of the passenger strand. As a result, an siRNA containing eight amide linkages is more active than the unmodified control. The results suggest that RNAi may tolerate even more extensive amide modification, which may be useful for optimization of siRNAs for *in vivo* applications.

## INTRODUCTION

Interest in synthetic chemistry of nucleic acids has been driven by the need for modified oligonucleotides for *in vivo* applications in antisense and RNA interference (RNAi) technologies (1,2). Chemical modifications have been instrumental in improving the stability of oligonucleotides in biological media. However, difficulties in targeted delivery, unfavorable pharmacokinetics and poor cellular uptake remain major obstacles for *in vivo* applications. These difficulties are in large part due to the negatively charged and polar phosphodiester backbone. Although replacement of the non-bridging oxygen with sulfur has showed promising improvement of antisense oligonucleotide properties (3), more dramatic modifications of the phosphodiester backbone have been little explored (4,5).

**Figure 1.**
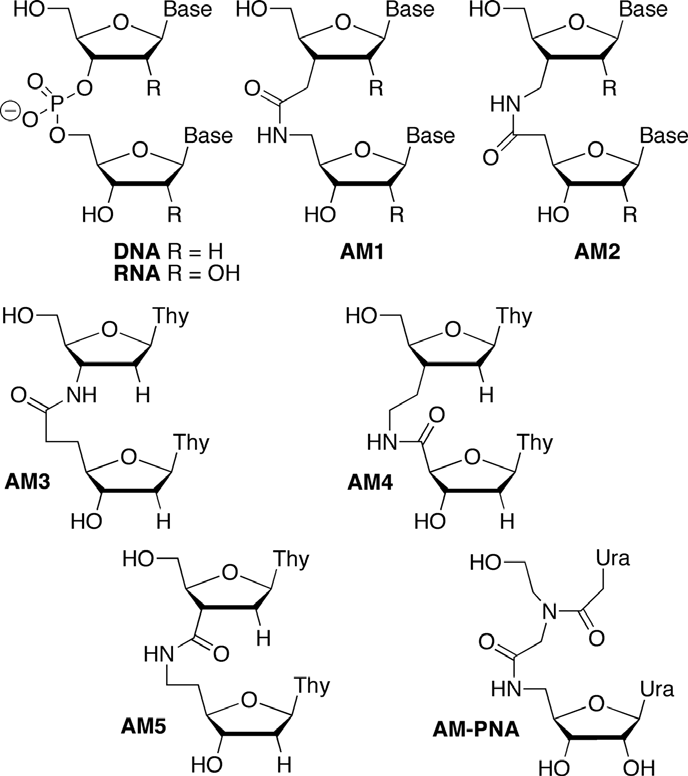
Structure of amide-modified DNA and RNA dimers.

Replacement of DNA phosphodiesters with non-ionic linkages to improve the enzymatic stability has been studied for antisense oligonucleotides (4,5). Among such linkages, amides (Figure 1) emerged as favorites because they were relatively easy to make by peptide-type couplings. Moreover, early results indicated that short DNA sequences with isolated amide linkages formed stable duplexes with complementary RNAs. Dimers **AM3**–**AM5** (Figure 1), the first amides studied in DNA, were found to destabilize DNA–RNA heteroduplexes by –1 to –4°C per modification (decrease in duplex melting temperature, *t*_m_) depending on the sequence context (6–8).

Among various isomeric linkages **AM1** and **AM2** were later identified as the best substituents for phosphodiester linkages in DNA (4). Depending on the sequence context, **AM1** and **AM2** modification of the DNA strand led to slight stabilization or destabilization (+0.9 to –0.8°C per modification) of the DNA–RNA heteroduplexes (9). Both modifications destabilized all-DNA duplexes by about –4°C per modification (9). **AM1**, first described independently by Just *et al.* (10) and De Mesmaeker *et al.* (11,12) in 1993–94, has become the most studied amide modification in DNA. While preliminary nuclear magnetic resonance (NMR) (13) and molecular modeling (14,15) studies suggested that **AM1** linkage adopted an A-like conformation in the DNA strand, a more detailed structure of an amide-modified oligodeoxynucleotide has not been determined to date. De Mesmaeker *et al.* (15) briefly explored **AM1** dimers derived from RNA (R = OH) and 2′-*O*-Me RNA (R = OCH_3_). They found that the 2′-*O*-Me modification increased thermal stability of duplexes, especially when placed on the sugar that has the amide modification attached to the C5′ (15,16). In contrast, the 2′-OH next to the amide linkage (on the sugar that has the amide modification attached to the C3′) decreased the thermal stability (15). However, rationalization of these observations was complicated by the fact that the amide modifications with RNA-like sugars were placed in the DNA strand of DNA–RNA heteroduplexes.

Early studies explored binding of modified DNA to RNA targets (formation of DNA–RNA heteroduplexes) because of the focus on potential antisense applications of the modified oligodeoxyribonucleotides (4). More recently, the discovery of RNAi revitalized the interest in chemical modifications of RNA (1,2). Robins *et al.* reported synthesis of **AM1** linked RNA dimers (17–19) and pentamers (20), but did not study the biophysical properties of these analogues. Rozners *et al.* found that both **AM1** and **AM2** dimers with either 2′-OH or 2′-*O*-Me sugars were well accommodated in all-RNA duplexes (21,22). While the effect of **AM1** on thermal stability was relatively small, **AM2** significantly increased stability of RNA duplexes (22). More recently, detailed thermodynamic and NMR structural studies by our group showed that **AM1** amides had surprisingly little effect on the A-type conformation, thermal stability and hydration of RNA duplexes (23). The local conformational changes caused by the amide linkage were easily accommodated by small adjustments in RNA structure suggesting that amides may be excellent mimics of phosphate linkages in RNA and promising modifications to optimize short interfering RNAs (siRNAs). Herein we extend these studies and present a crystal structure that provides detailed conformational information on how the amide is accommodated in the RNA duplex and further illustrates the excellent hydration and conformational adaptability of amide linkages in RNA.

While our studies on amide-modified RNA were in progress, Iwase *et al.* showed that modification of the 3′-overhangs of an siRNA with two **AM1** (Figure 1) linkages increased the enzymatic stability but did not decrease RNAi activity (24,25). However, this was not unexpected because the 3′-overhangs in general tolerate modifications much better than the internal positions of siRNAs. Gong and Desaulniers studied siRNAs containing an amide linkage derived from insertion of a PNA monomer (**AM-PNA**, Figure 1) (26). The PNA-derived amide linkage was tolerated in the 3′-overhang of the passenger strand. However, internal modification of the guide strand led to significant loss of silencing activity. Potenza *et al.* also reported that replacement of the phosphates in 3′-overhangs with two PNA linkages increased the enzymatic stability of siRNAs but did not affect their RNAi activity (27). Herein we show that amide linkages are not only tolerated at internal positions of both guide and passenger strands of siRNAs but may increase the silencing activity when placed near the 5′-end of the passenger strand. These findings are unexpected and raise the possibility that RNAi may tolerate and benefit from even more substantial modifications than the ones tried so far.

## MATERIALS AND METHODS

### Synthesis and purification of amide-modified RNA

Amide-modified oligoribonucleotides were prepared on a 1 μmol scale using the standard 2′-*O*-TOM RNA phosphoramidite (Glen Research) synthesis protocol on an Expedite 8909 Nucleic Acid Synthesis System. A standard coupling time was used for the dimeric phosphoramidites **11a** and **11b**. Cleavage of oligoribonucleotides from solid support and deprotection of the heterocyclic amino groups was done by treating the solid support with a mixture of ethanolic methylamine and aqueous methylamine (1:1) solution at room temperature for 24 h. The cleavage solution was freeze-dried and the residue was dissolved in dimethyl sulfoxide (100 μl). Triethylamine trihydrofluoride (125 μl) was added and the reaction mixture was left for 24 h at room temperature to remove the 2′-*O*-TBS and 2′-*O*-TOM protecting groups. The reaction mixtures were diluted with water (1275 μl) and desalted on a Sephadex C25 NAP column in accordance with the manufacturer's recommendations. The amide-modified oligoribonucleotides were analyzed by reverse-phase high-performance liquid chromatography (HPLC) with an XBridge™ C18 column (4.6 × 150 mm, 3.5 μm, 1 ml/min) and purified by reverse-phase HPLC with an XBridge™ C18 column (10 × 150 mm, 5 μm, 2 ml/min) using a linear gradient (2–40%) of acetonitrile in 0.1 M triethylammonium acetate buffer, pH 7.0 (for HPLC chromatograms, see Supplementary Figures S1 and S12–S27). The fractions containing the target material were freeze-dried. The residue was dissolved in water (5 ml) and freeze-dried again. To remove the bulk of triethylammonium salts, the latter operation (dissolve and freeze-dry) was repeated one or two times. To completely convert the amide-modified RNAs into the sodium salt form, the samples were dissolved in phosphate buffer (0.5 ml of 20 mM sodium phosphate, 80 mM NaCl, 50 μM EDTA, pH 6.3) and desalted on a Sephadex C25 NAP column. The residue was dialyzed against pure water using a Float-A-Lyzer G2 (MWCO: 100–500 D, Spectrum Laboratories) for 10 h and changing the water after 2 and 6 h. Quantification of amide-modified RNAs was done using the nearest-neighbor approximation (28). The identity of the siRNA oligonucleotides was confirmed by electrospray ionization mass spectrometry (for details, see Supplementary Data).

### X-ray crystallography

**Figure 2.**
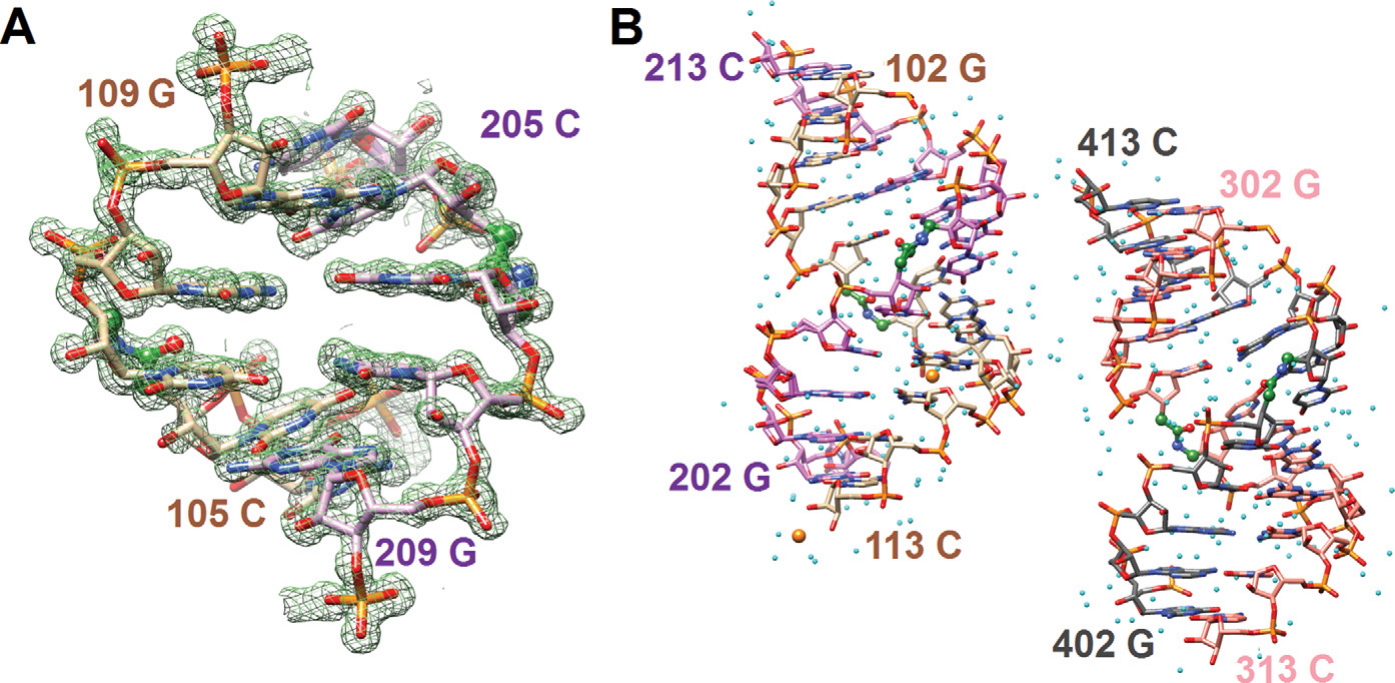
(**A**) Example of the quality of the final Fourier (2Fo*_o_*-Fc*_c_*) sum electron density (∼1.2 σ threshold). (**B**) Overall view of **RNA13** duplexes in the unit cell. Residues in the four strands are numbered from 101–113, 201–213, 301–313 and 401–413, respectively. Carbon atoms of strands 1, 2, 3 and 4 are colored in beige, pink, salmon and gray, respectively, and red, blue and orange mark oxygen, nitrogen and phosphorus atoms, respectively. Strontium ions are shown as orange spheres and smaller spheres in cyan represent water molecules. The carbon atoms in the amide carbonyls and the adjacent linking carbon atoms are colored in green and the C-NH-CO-C linkages are highlighted in ball and stick mode.

Crystals of 5′-r(UpGpApGpCp**UaU**pCpGpGpCpUpC)-3′ (**RNA13**) were grown by the hanging-drop vapor diffusion technique using the Nucleic Acid Miniscreen (Hampton Research, Aliso Viejo, CA) (29) and later optimized by varying the concentration of strontium chloride. The 13-mer amide RNA crystallized from two conditions: 1) droplets (2 μl) containing oligonucleotide (0.6 mM), sodium cacodylate (20 mM, pH 6.0), sodium chloride (40 mM), barium chloride (10 mM), spermine tetrahydrochloride (6 mM), and 2-methyl-2,4-pentanediol (MPD; 5% (v/v)) and 2) droplets (2 μl) containing oligonucleotide (0.6 mM), sodium cacodylate (20 mM, pH 6.0), strontium chloride (45 mM), magnesium chloride (10 mM), spermine tetrahydrochloride (6 mM), and 5% MPD (v/v), that were equilibrated against a reservoir of MPD (1 ml, 35%). All crystals were mounted in nylon loops without further cryo-protection and frozen in liquid nitrogen. Diffraction data were collected on the 21-ID-D beam line of the Life Sciences Collaborative Access Team (LS-CAT) at the Advanced Photon Source (APS), located at Argonne National Laboratory (Argonne, IL). The wavelength was tuned to 0.76 Å for crystals containing Sr^2+^ (for Ba^2+^ containing crystals data were collected at 1.77 Å) and data were collected at 110 K using a MARCCD 300 detector. Diffraction data were integrated, scaled and merged with the XDS package (30). Selected crystal data and data collection parameters are listed in Supplementary Table S1. The structure of **RNA13** contains two duplexes in space group P1 and was phased by single wavelength anomalous dispersion (SAD) of the Sr^2+^ data using the program SHELXD in the SAD mode, followed by density modification using SHELXE (31). Building of the models was performed with COOT (32). The initial orientations of duplexes were optimized by several rounds of rigid body refinement in Refmac5 (33). Refinement was then continued in Refmac5, using the maximum likelihood residual method. Subsequent refinement cycles were carried out using the program SHELXL (31), keeping aside 5% of the reflections to compute the R-free (34). Following a few cycles of refinement in SHELXL, phosphate groups were replaced by amide linkages and the dictionary was adapted to account for the altered backbone chemistry. Ions and water molecules were placed at sites of peaks in the Fourier 2*F*o-*F*c sum and *F*o-*F*c difference electron density maps, and accepted on the basis of standard distance criteria. Final refinement parameters and deviations from ideal geometries are listed in Supplementary Table S1. An example of the quality of the final electron density is depicted in Figure 2A and an overall view of the two duplexes is shown in Figure 2B. While phasing by Sr^2+^-SAD was thus successful in the case of **RNA13** crystals; we have so far been unable to phase the Ba^2+^ crystal form. Final coordinates and structure factors for **RNA13** have been deposited in the Protein Data Bank (http://www.rcsb.org). The PDB ID code is 4O41.

### siRNA activity test

HeLa cells were plated in 96-well plates (1 × 10^4^ cells/well) for 24 h before transfection. On the day of transfection, RNA-lipid complexes were introduced into each well of cells (0.1–100 nM RNA for HeLa cells 0.2 ml/well DharmaFECT 1). siGENOME Non-Targeting siRNA #1 (r(UAGCGACUAAACACAUCAAUU), Thermo Fisher, catalogue # D-001210–01) was used as a non-target control (NTC). NTC and siRNAs targeting the Cyclophilin B (PPIB) gene were both titrated separately at the same concentrations to show that they behave in the same way and do not cause toxicity (cell viability was determined using a Resazurin assay, Supplementary Figures S4–S10). Twenty-four hours post-transfection, the level of target knockdown was assessed using a branched DNA (bDNA) assay specific for the targets of interest according to the manufacturer's instructions (QuantiGene branched DNA signal amplification kit; Panomics, Fremont, CA). Cells were lysed and directly added to the bDNA assay plate without nucleic acid purification or cDNA synthesis steps that can introduce bias. Except for sequences having amide modifications at the 3′-end, modified guide strands were chemically 5′-phosphorylated because of concern that amide modifications close to the 5′-end may interfere with enzymatic phosphorylation normally observed in cells. The modified passenger strands were prepared in 5′-OH and 5′-phosphorylated form. This was done to study different potential phosphorylation states. Appropriate 5′-OH and 5′-phosphorylated unmodified controls were prepared and used and the conclusions were based on the relative activities. The results presented in Figures 5–9 are expressed as ratios of target Cyclophilin B mRNA expression (PPIB) and housekeeping mRNA expression (GAPDH) and are averages of three experiments (biological triplicates from three separate wells of cells). The standard deviations are shown in the length of the error bars. UV melting experiments on siRNA duplexes were carried out in 25 mM NaCl, 15 mM sodium citrate, 1 mM EDTA at pH 7.2 using a Thermo Scientific Evolution 3000.

**Figure 3.**
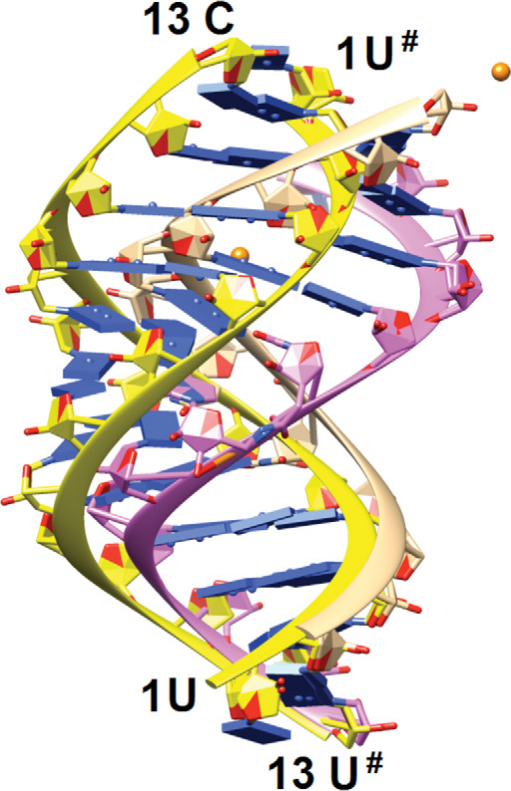
Comparison between the conformation of the amide-modified **RNA13** duplex (backbone ribbons of strands 1 and 2 colored in beige and pink, respectively) and that of the native 13mer RNA duplex (PDB ID 413D; backbone ribbons colored in yellow). The image was generated by overlaying terminal base pairs at one end of the duplexes. The view is across the major and minor grooves, strontium ions are orange spheres and base pairs are depicted as blue slabs.

**Figure 4.**
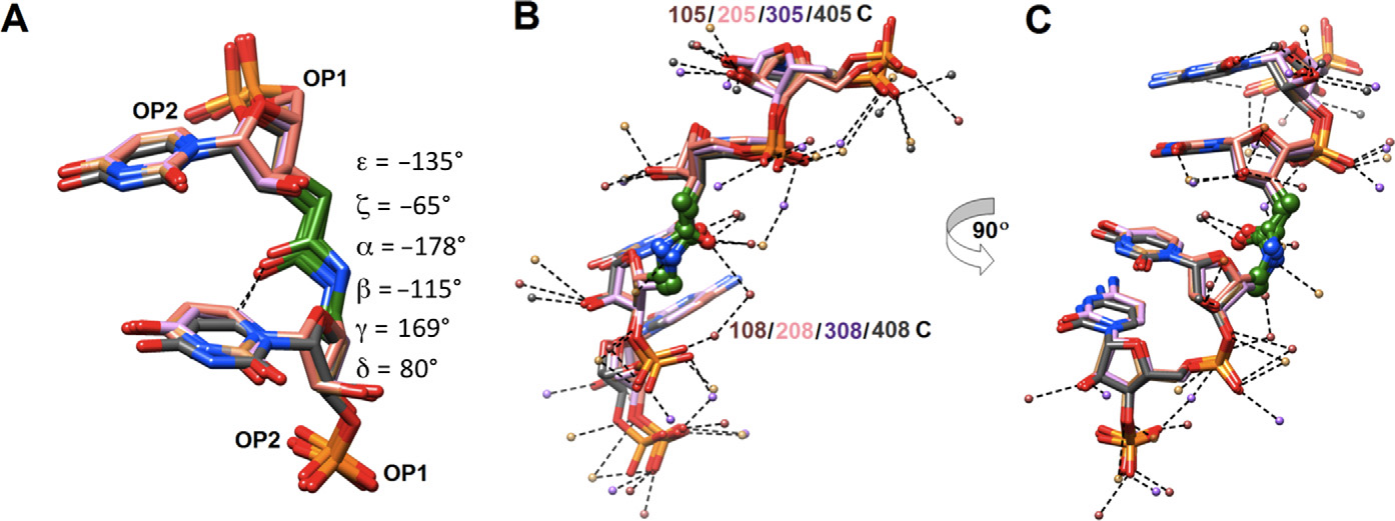
(**A**) Conformation of the amide moiety. The image depicts a superimposition of the four independent amide linkages with average values of torsion angles indicated on the right. (**B** and **C**) Major and minor groove hydration in the central portion of the **RNA13** duplex. Two views of the overlay of four strands (only residues 5–8 are shown) depict (**B**) major groove hydration and (**C**) minor groove hydration (obtained after rotation by 90? around the vertical). Carbon atoms of strands 1, 2, 3 and 4 are colored in beige, pink, salmon and gray, respectively. The small spheres represent water molecules with colors matching their respective strands and dashed lines indicate hydrogen bonds. The amide carbonyls and the adjacent linking carbon atoms are colored in green and C-NH-CO-C linkages are highlighted in ball and stick mode.

**Figure 5.**
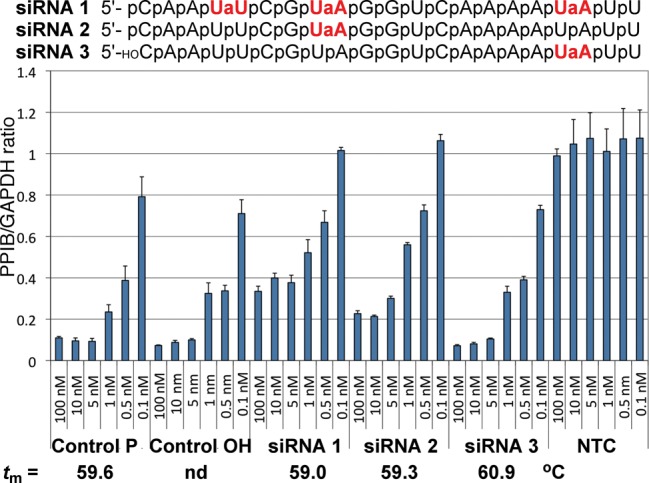
RNAi activity of siRNAs with amide modifications in the guide strand. The results are expressed as ratios of target mRNA expression (PPIB) and housekeeping mRNA expression (GAPDH) and are averages of three replicates; the standard deviations are shown in the length of the error bars. **Control P** and **Control OH** are unmodified siRNAs with 5′-phosphate or 5′-OH, respectively. **NTC** is a non-targeting control siRNA used as a negative control; *t***_m_** values are the melting temperatures of the siRNA duplexes.

**Figure 6.**
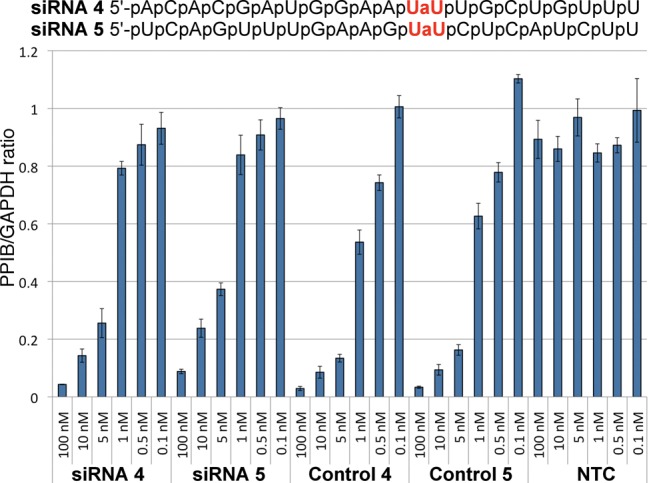
Comparison of the effect of amide modification at the same position in two different guide strands PPIBHFS438 (**siRNA 4**) and PPIBHFS542 (**siRNA 5**). **Control 4** and **Control 5** are unmodified sequences of **siRNA 4** and **siRNA 5**, respectively. For other notes, see Figure 5.

**Figure 7.**
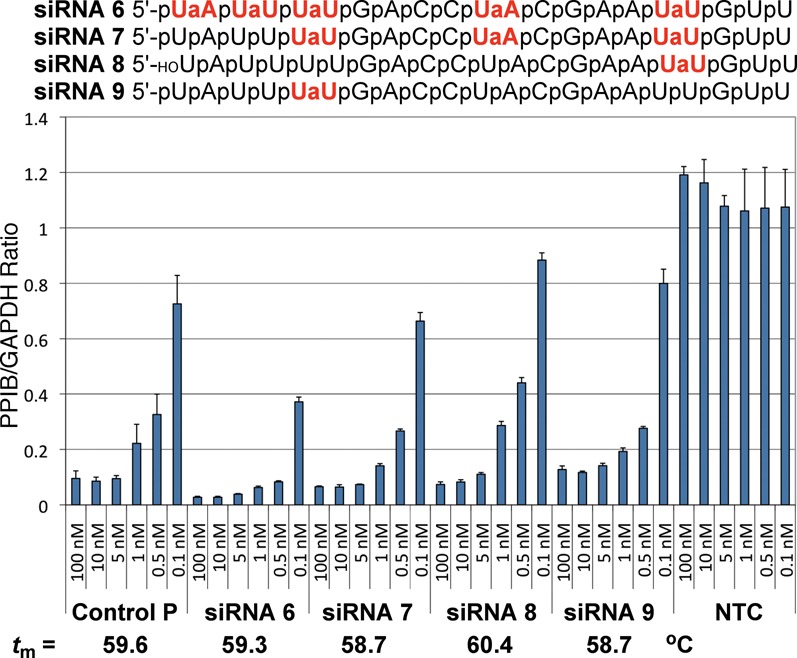
RNAi activity of siRNAs with amide modifications in the passenger strand. For other notes, see Figure 5.

**Figure 8.**
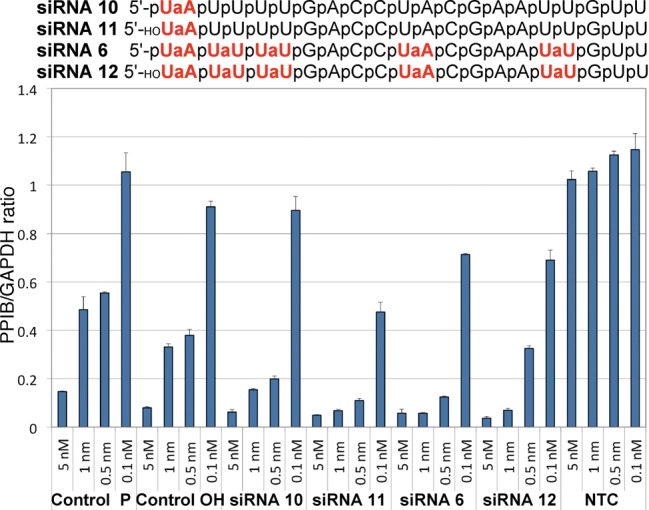
Comparison of the effect of 5′-phosphorylation on silencing activity of amide siRNAs. For other notes, see Figure 5.

**Figure 9.**
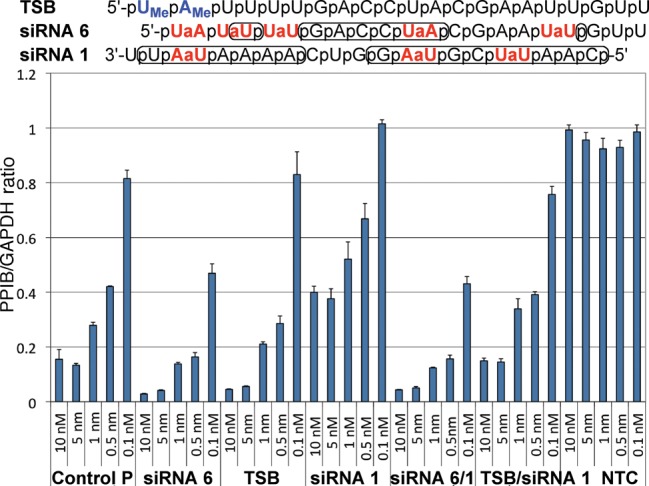
Comparison of the abilities of 2′-OMe (TSB) and amide-modified (**siRNA 6**) passenger strands to enhance RNAi activity of amide-modified guide strand **siRNA 1**. Phosphates implicated in RNA–protein interactions are circled in RNA sequences; for other notes, see Figure 5.

## RESULTS

### Synthesis of amide-modified RNA

**Scheme 1.**
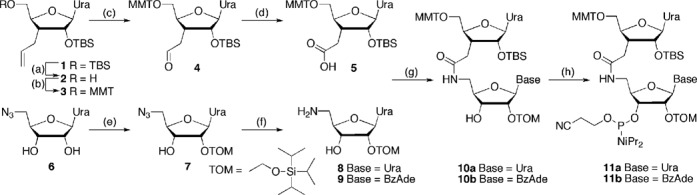
Synthesis of dimeric r(U_AM1_U) and r(U_AM1_A) phosphoramidites. Steps: (a) TFA, tetrahydrofuran, H_2_O 0°C, 4 h, 92%; (b) p-methoxytrityl chloride, pyridine, 0°C to rt, 14 h, 85%; (c) OsO_4_, 4-methylmorpholine N-oxide, dioxane, rt, 10 h, then NaIO_4_ in water, rt, 12 h, 95%; (d) NaClO_4_, NaH_2_PO_4_, 2-methylbut-2-ene, *t*-BuOH, THF water, rt, 1 h, 82%; (e) DIEA, Bu_2_SnCl_2_, dichloroethane, rt, 1 h, then add TOM-Cl, 80°C, 45 min, 39%; (f) H_2_S, pyridine, water, rt, 14 h 93%; (g) HBTU, HOBt, DIEA, CH_2_Cl_2_, rt, 12 h, 95% **11a**, 86% **11b**; (h) DIEA, ClP(OCH_2_CH_2_CN)N(iPr)_2_, CH_2_Cl_2_, rt, 7 h, 71% **11a**, 60% **11b**.

Synthesis of amide-linked dimers started with optimization of the previous procedures (22) for preparation of the uridine carboxylic acid **5** (Scheme 1). The 5′-*O*-TBS group was selectively cleaved using trifluoroacetic acid (TFA) and replaced with the 5′-*O*-methoxytrityl (MMT) group in **3**. Two-step oxidative degradation of the alkene gave the carboxylic acid **5**, which had the more stable 2′-*O*-TBS instead of the previously used 2′-*O*-Ac protection (23). The new route was also one step shorter. The uridine **8** and adenosine **9** amines were prepared as previously described (23). N,N,N′,N′-Tetramethyl-O-(1H-benzotriazol-1-yl)uronium hexafluorophosphate (HBTU) mediated coupling of **5** with aminouridine **8** or aminoadenosine **9** gave the r(U**_AM1_**U) and r(U**_AM1_**A) dimers **10a** and **10b**, respectively (Scheme 1), which were converted into **11a** and **11b** in one standard step of phosphoramidite synthesis (for experimental details, see Supplementary Data). We chose modification of U and A nucleosides because of more straightforward chemical synthesis than that of C and G nucleosides. Dimers **11** were used together with common 2′-*O*-TOM protected ribonucleoside monomers (Glen Research) to synthesize a series of amide-modified RNAs according to standard phosphoramidite chemistry on an Expedite 8909 instrument.

### X-ray crystal structure of amide-modified RNA

Out of several self-complementary RNAs with a single isolated amide linkage we synthesized for crystallography studies, RNA 13mer rUGAGC**U_AM1_U**CGGCUC (abbreviated **RNA13**) gave well diffracting crystals. The melting temperatures of **RNA13** (*t*_m_ = 78°C) and the unmodified RNA (*t*_m_ = 79°C) were similar. The circular dichroism spectra had significant differences (Supplementary Figure S2) but were not expected to be similar because of different conformations of the respective crystal structures (see Figure 3). This 13mer features non-canonical UoG wobble and U*C base pairs in the middle of the duplex and 5′-terminal U overhangs. The space group was P1 with two 13mer duplexes in the unit cell. The structure was phased by strontium single wavelength anomalous dispersion (Sr-SAD) using a wavelength of 0.76 Å for data collection on the LS-CAT 21-ID-D beamline at the APS (Argonne, IL). Two partially occupied strontium ions were retrieved and the experimental density allowed building of complete models of the two duplexes without the dangling Us. Their structures along with 269 water molecules were refined to an R-factor of 17.1% (R-free 23.5%) at a resolution of 1.2 Å. The 5′-terminal uridines are disordered in all four strands and were omitted from the refinement. An electron density map is depicted in Figure 2A. Both strontium ions are associated with a single duplex (Figure 2B). One is coordinated to N7 and O6 of G from a G:C pair flanking the central stretch of mismatch pairs. The other is coordinated to O2′ and O3′ of the 3′-terminal C of the first strand, reminiscent of the coordination modes of platinum and osmium in crystals of phenylalanine tRNA (35).

The **RNA13** duplex adopts an A-like conformation with modulated groove widths as a result of the UoG wobble and U*C pairs in the central part. Thus, the minor groove width along the duplex as measured by phosphate–phosphate distances varies between 15 and 18 Å, whereby the section with mismatch base pairs is notably wider. The conformation of **RNA13** differs significantly from that displayed by the native RNA 13mer (Figure 3). The native duplex is of the so-called A′-form with 12 base pairs per turn compared to 11 base pairs in the canonical A-form duplex (PDB ID 413D) (36). Moreover, the base pairs in the native RNA exhibit smaller inclination angles and the major groove is considerably wider (and the minor groove therefore contracted) relative to the **RNA13** duplex. Substantial differences between the two duplexes are also illustrated by the fact that attempts to solve the **RNA13** structure with molecular replacement and using the native RNA duplex as the search model failed consistently.

The reasons for this switch from the A′- to the A-conformation are complex. It is likely that relatively subtle changes such as the replacement of a phosphate by an amide moiety, altered packing forces or ionic strength, or the presence of a ligand can trigger the conversion from one form to the other. Interestingly, the native RNA duplex is located on a dyad in space group C2, whereas **RNA13** duplexes are located in general positions in P1. The different packing modes go along with a marked reduction in the volume per base pair of about 25% in the case of **RNA13** duplexes. The two independent **RNA13** duplexes in space group P1 also exhibit subtle differences in their overall conformations (Supplementary Figure S3).

Amide moieties in all four strands assume similar conformations (Figure 4A). The backbone torsion angles fall into the following ranges: *ac-* (ϵ), *sc-* (ζ), *ap* (α), *ac-* (β), *ap* (γ) and *sc+* (δ). By comparison, the standard A-form RNA torsion angle ranges are *ap* (ϵ), *sc-* (ζ), *sc-* (α), *ap* (β), *sc+* (γ) and *sc+* (δ). The structure reveals that the amide carbonyl group is rotated into the major groove and thus assumes an orientation that is similar to that of the P–OP2 bond (Figure 4A). In the case of the U**_AM1_**U step, this orientation of the amide C = O bond results in a relatively short contact between the amide oxygen and uracil C6–H6 (average distance 3.4 Å) that is consistent with formation of a C–H…O hydrogen bond.

Analysis of the water structure around the backbones of **RNA13** duplexes shows that phosphate and amide carbonyl oxygens as well as 2′-hydroxyl groups are well hydrated (Figure 4B and C). Compared to 2′-OH, the amide N–H function appears to be a less attractive donor for H-bonding interactions. Along the rim of the major groove OP2 phosphate oxygens are bridged by single water molecules (Figure 4B), a pattern long ago noticed in A-form DNA and RNA duplexes (37,38). Compared with the distances between adjacent OP2 atoms (as short as 5 Å), the distances between amide carbonyl oxygen and OP2s from 5′- and 3′-adjacent residues are slightly longer (ca. 6 Å), resulting in tandem waters linking the carbonyl group and phosphate oxygens (Figure 4B).

### RNAi activity of amide-modified siRNAs

Using the dimeric r(U**_AM1_**U) and r(U**_AM1_**A) phosphoramidites, we first introduced up to three amide modifications in the guide strand of siRNA (PPIBHFS308) targeting the Cyclophilin B (PPIB) gene. The modified siRNAs (combined with an unmodified passenger strand) and appropriate unmodified controls were transfected into HeLa cells using DharmaFECT 1 (Thermo Fisher) for 24 h. PPIB expression was assayed by branched DNA (bDNA Quantigene) relative to GAPDH (housekeeping gene) expression at different siRNA concentrations (100–0.1 nM). Consistent with Iwase's results (24,25), insertion of one amide at the 3′-end of **siRNA 3** had little effect on silencing activity (Figure 5). One modification in the middle of the guide strand (**siRNA 2**) was also relatively well tolerated, which was in contrast to Gong and Desaulniers’ report (26) that the **AM-PNA** linkage in the middle of the guide strand significantly decreased RNAi activity. Increasing the number of amide modifications in the guide strand **siRNA 1** decreased the silencing activity. It is very likely that the reduced RNAi activity was due to inefficient loading of the amide-modified guide strand in RNA-induced silencing complex (RISC), as discussed below.

In crystal structures of complexes between oligonucleotides and Ago or Ago domains, the protein recognizes the overall shape of siRNA by hydrogen bonding to backbone phosphates (39–43). Except for the preferential recognition of U and A at the 5′-end of guide strand by the MID domain (39,40,44), Ago does not make specific interactions to nucleobases. Since the interactions of Ago with siRNA should have little sequence specificity, it is conceivable that the effect of amide modification would depend mostly on its location along the siRNA duplex and not on the specific sequence of siRNA. To test this notion, we selected two guide strands targeting the Cyclophilin B gene, PPIBHFS438 (**siRNA 4**) and PPIBHFS542 (**siRNA 5**) that allowed for an amide modification at the same position, between nucleosides 12 and 13 (Figure 6). Similar to **siRNA 2**, this position is close to the mRNA cleavage site and may be sensitive to modifications. As expected, amide-modified **siRNA 4** and **siRNA 5** had somewhat decreased RNAi activity compared to unmodified controls. Most importantly, the change in RNAi activity upon amide modification was about the same for both sequences. **Control 5** was somewhat less active than **Control 4** and this difference was reflected in **siRNA 4** and **siRNA 5** as well. These results provided preliminary evidence that amide modifications are likely to have position-specific, but not sequence-dependent effects on RNAi activity. In other words, amide modification may be expected to have a similar effect at the same position in different siRNAs.

The effect of amide modifications in the passenger strand on silencing activity was studied using the same siRNA (PPIBHFS308) as for the guide strand. The most striking result was that five amide modifications in **siRNA 6** increased RNAi activity (Figure 7). The most favorable effect came from the modifications at the 5′-end because **siRNA 7** with three modifications in the middle and at the 3′-end of the siRNA had activity similar to unmodified control. A single amide in **siRNA 9** appeared to have relatively little effect, while the 3′-end modification in **siRNA 8** slightly reduced the activity.

Testing individual 5′-end modifications revealed that an amide between nucleosides 1 and 2 was the most beneficial (Figure 8 and Supplementary Figure S11), at least in the 5′-OH series. These experiments also revealed an interesting effect of 5′-phosphorylation on the activity of amide-modified passenger strands. The guide strand requires 5′-phosphorylation to be loaded in RISC. Conversely, 5′-phosphorylation of the passenger strand is not desirable as it may decrease the activity by enhancing loading of the passenger strand instead of the guide strand. Chemical modifications of the passenger strand that prevent 5′-phosphorylation have been used to improve RNAi activity and suppress off-target effects (45). As expected, the non-phosphorylated **siRNA 11** was somewhat more active than the phosphorylated **siRNA 10** (Figure 8). Interestingly, phosphorylation had little effect on the heavily modified **siRNA 6** and **siRNA 12**, indicating that the amide modifications are more important for directing the RISC loading than phosphorylation.

Consistent with our earlier biophysical studies (22,23), the **AM1** modification had relatively little impact on thermal stability (melting temperatures, *t*_m_) of siRNA duplexes (Figures 5 and 7). The lack of correlation between *t*_m_ and silencing activity suggested that the observed changes in activity were likely due to specific siRNA–Ago interactions and not due to modulation of the thermodynamic stability of siRNA duplexes.

Thermodynamic strand bias (TSB, Figure 9), a passenger strand having two 2′-OMe groups at the 5′-end, is a modification for increasing activity and selectivity by enforcing the loading of the guide strand in RISC (46). Both TSB and amide-modified passenger strand improved siRNA activity in a similar way. Interestingly, the amide-modified passenger **siRNA 6** was more efficient in improving the silencing activity of the amide-modified guide **siRNA 1** (c.f., Figure 9, **siRNA 6/1** and TSB**/siRNA 1**). These data show that suitable amide modifications in the passenger strand are capable of forcing effective loading of the guide strand in RISC. Another striking result was the ability of amide modifications in the passenger strand **siRNA 6** to more than compensate for RNAi activity decrease upon modification of the guide strand **siRNA 1** (Figure 9). The highly modified **siRNA 6/1** (eight amide linkages) was more active than the unmodified control.

## DISCUSSION

siRNAs have been modified mostly in the ribose moiety and to lesser extent in the heterocyclic bases (1,2). Apart from phosphorothioates (47–49), phosphorodithioates (50) and boranophosphates (51), chemical modification of the phosphate backbone in siRNAs has been little explored. Our early work suggested that **AM1** amides are structurally fit as replacements of phosphodiesters in A-form RNA duplexes (21,22). More recently, we used NMR and osmotic stress to show that **AM1** linkages did not disturb the conformation and hydration of double-stranded RNA (23). Before the present study, a crystal structure of an **AM1** modified RNA duplex that would confirm these notions and provide a detailed structure of amide hydration had not been solved.

The present crystal structure provides unique insights into the conformation and hydration of the amide linkage and confirms our earlier observations that **AM1** amide is surprisingly efficient in mimicking the phosphate backbone in RNA (22,23). Consistent with our previous osmotic stress data (23), amide linkages are well hydrated by tandem waters linking the carbonyl group and phosphate oxygens (Figure 4B). The amide conformation in the present structure is similar to that observed in our earlier NMR structure (23). Our most recent NMR and osmotic stress study showed that even three consecutive amide linkages had relatively little effect on structure and hydration of a short RNA duplex (52). The alignment of the amide carbonyl group in an orientation that is similar to that of the P–OP2 bond is noteworthy and suggests that the amide C = O may be able to mimic non-bridging P–O in RNA–protein interactions.

Recent crystal structures of siRNAs bound to Argonaute 2 (Ago2), the key protein of the RISC, show that most of the phosphates of the guide (antisense) strand make hydrogen bonds to amino acid side chains of Ago2 (39–43). In contrast, the passenger (sense) strand is partially solvent exposed and phosphates 1, 2, 5, and 13–17 do not interact with the protein (43). The phosphates implicated in RNA–protein interactions are circled in RNA sequences in Figure 9. Accordingly, one would expect that replacement of the guide strand phosphates with amides might significantly decrease RNAi activity, while similar modification of the passenger strand might be tolerated at certain positions. This would be in line with previous results on other modifications that showed the guide strand to be more sensitive to backbone modifications than the passenger strand (26,51). From this perspective, the results in Figure 5 were encouraging because all three phosphates in the modified guide strand **siRNA 1** form hydrogen bonds to Piwi and Paz domains of Ago in crystal structures (39,42,43), yet the loss of activity was less than that observed for a single **AM-PNA** linkage in the middle of the guide strand (26). These results suggest a hypothesis that amide C = O may be able to interact with amino acid side chains of Ago in a manner similar to the non-bridging P–O of unmodified siRNAs.

The above hypothesis was also supported by the lack of strong correlation between RNAi activities of modified passenger strands and whether the phosphate modified was or was not shown to hydrogen bond with Ago. Instead, the results suggested that modification of the 5′-end of the passenger strand might have an unexpected beneficial effect. While future studies will be needed to confirm the generality of these results, the observation that siRNAs having the heavily modified passenger strand **siRNA 6** were among the most active was surprising and encouraging. The increase in RNAi activity of **siRNA 6** is most likely caused by favorable loading of the associated guide strand into the RISC.

Perhaps the most significant result of the present study is that the combination of highly modified **siRNA 6** and **siRNA 1** (an siRNA with eight amide linkages) was more active than the unmodified control. This result leads us to hypothesize that RNAi may tolerate significant guide strand modifications when combined with properly modified passenger strands that enhance the RISC loading. It is conceivable that optimization of siRNAs for *in vivo* applications will require more extensive amide modification than in the present study. Future work will explore introduction of consecutive amide linkages in siRNAs. Toward this goal we recently reported that introduction of three consecutive amide linkages between the four uridines at the 5′-end of siRNA PPIBHFS308 had relatively small impact on RNAi activity (52). It should be noted that the three consecutive amide linkages did not include the 5′-UaA modification (as in **siRNA 10** and **11**) that may be the most beneficial for RNAi activity. Taken together, our results suggest that optimized combination of amide-modified guide and passenger strands may have potential to improve the properties of siRNAs critical for *in vivo* applications.

## CONCLUSION

Our crystallographic and RNAi activity studies are consistent with earlier biophysical and NMR results (23) and, taken together, strongly suggest that amides are excellent structural mimics of the phosphate backbone in RNA and may have the potential to improve the properties of siRNAs. The crystal structure of an amide-modified RNA illustrates how the amide linkage is accommodated in an A-form duplex. Consistent with previous osmotic stress results (23), amides are well hydrated. The amide linkages are not only tolerated at internal positions of both guide and passenger strands of siRNAs, but may also increase the silencing activity when placed near the 5′-end of the passenger strand. As a result, an siRNA containing eight amide linkages was more active than the unmodified control. Taken together, these results are encouraging for further exploration of amides and other non-ionic backbone modifications as means of improving the properties of siRNAs.

## Accession Number

PDB ID 4O41

## SUPPLEMENTARY DATA

Supplementary Data are available at NAR Online.

SUPPLEMENTARY DATA

## References

[1] Watts J.K., Corey D.R. (2012). Silencing disease genes in the laboratory and the clinic. J. Pathol..

[2] Deleavey G.F., Damha M.J. (2012). Designing chemically modified oligonucleotides for targeted gene silencing. Chem. Biol..

[3] Levin A.A. (1999). A review of issues in the pharmacokinetics and toxicology of phosphorothioate antisense oligonucleotides. Biochim. Biophys. Acta.

[4] Freier S.M., Altmann K.H. (1997). The ups and downs of nucleic acid duplex stability: structure-stability studies on chemically-modified DNA:RNA duplexes. Nucleic Acids Res..

[5] De Mesmaeker A., Altmann K.-H., Waldner A., Wendeborn S. (1995). Backbone modifications in oligonucleotides and peptide nucleic acid systems. Curr. Opin. Struct. Biol..

[6] Lebreton J., De Mesmaeker A., Waldner A., Fritsch V., Wolf R.M., Freier S.M. (1993). Synthesis of thymidine dimer derivatives containing an amide linkage and their incorporation into oligodeoxyribonucleotides. Tetrahedron Lett..

[7] De Mesmaeker A., Lebreton J., Waldner A., Fritsch V., Wolf R.M., Freier S.M. (1993). Amides as substitute for the phosphodiester linkage in antisense oligonucleotides. Synlett.

[8] De Mesmaeker A., Lebreton J., Waldner A., Fritsch V., Wolf R.M. (1994). Replacement of the phosphodiester linkage in oligonucleotides: comparison of two structural amide isomers. Bioorg. Med. Chem. Lett..

[9] Lebreton J., Waldner A., Fritsch V., Wolf R.M., De Mesmaeker A. (1994). Comparison of two amides as backbone replacement of the phosphodiester linkage in oligodeoxynucleotides. Tetrahedron Lett..

[10] Idziak I., Just G., Damha M.J., Giannaris P.A. (1993). Synthesis and hybridization properties of amide-linked thymidine dimers incorporated into oligodeoxynucleotides. Tetrahedron Lett..

[11] De Mesmaeker A., Waldner A., Lebreton J., Hoffmann P., Fritsch V., Wolf R.M., Freier S.M. (1994). Amides as a new type of backbone modifications in oligonucleotides. Angew. Chem., Int. Ed. Engl..

[12] Lebreton J., Waldner A., Lesueur C., De Mesmaeker A. (1994). Antisense oligonucleotides with alternating phosphodiester-”amide-3” linkages. Synlett.

[13] Blommers M.J.J., Pieles U., De Mesmaeker A. (1994). An approach to the structure determination of nucleic acid analogs hybridized to RNA. NMR studies of a duplex between 2’-OMe RNA and an oligonucleotide containing a single amide backbone modification. Nucleic Acids Res..

[14] Nina M., Fonne-Pfister R., Beaudegnies R., Chekatt H., Jung P.M.J., Murphy-Kessabi F., De Mesmaeker A., Wendeborn S. (2005). Recognition of RNA by amide modified backbone nucleic acids: molecular dynamics simulations of DNA-RNA hybrids in aqueous solution. J. Am. Chem. Soc..

[15] De Mesmaeker A., Lesueur C., Bevierre M.O., Waldner A., Fritsch V., Wolf R.M. (1996). Amide backbones with conformationally restricted furanose rings: Highly improved affinity of the modified oligonucleotides for their RNA complements. Angew. Chem., Int. Ed..

[16] De Mesmaeker A., Lebreton J., Jouanno C., Fritsch V., Wolf R.M., Wendeborn S. (1997). Amide-modified oligonucleotides with preorganized backbone and furanose rings. Highly increased thermodynamic stability of the duplexes formed with their RNA and DNA complements. Synlett.

[17] Robins M.J., Sarker S., Xie M., Zhang W., Peterson M.A. (1996). Nucleic acid related compounds. 90. Synthesis of 2’,3’-fused (3.3.0) γ-butyrolactone-nucleosides and coupling with amino-nucleosides to give amide-linked nucleotide-dimer analogs. Tetrahedron Lett..

[18] Robins M.J., Zou R., Guo Z., Wnuk S.F. (1996). Nucleic acid related compounds. 93. A solution for the historic problem of regioselective sugar-base coupling to produce 9-glycosylguanines or 7-glycosylguanines. J. Org. Chem..

[19] Peterson M.A., Nilsson B.L., Sarker S., Doboszewski B., Zhang W., Robins M.J. (1999). Amide-linked ribonucleoside dimers derived from 5’-amino-5’-deoxy- and 3’-(carboxymethyl)-3’-deoxynucleoside precursors. J. Org. Chem..

[20] Robins M.J., Doboszewski B., Nilsson B.L., Peterson M.A. (2000). Nucleic acid related compounds. 112. Synthesis of amide-linked [(3’)CH2CO-NH(5’)] nucleoside analogs of small oligonucleotides. Nucleosides Nucleotides Nucleic Acids.

[21] Rozners E., Strömberg R. (1997). Synthesis and properties of oligoribonucleotide analogs having amide (3’-CH2-CO-NH-5’) internucleoside linkages. Nucleosides Nucleotides.

[22] Rozners E., Katkevica D., Bizdena E., Strömberg R. (2003). Synthesis and properties of RNA analogs having amides as interuridyl linkages at selected positions. J. Am. Chem. Soc..

[23] Selvam C., Thomas S., Abbott J., Kennedy S.D., Rozners E. (2011). Amides as excellent mimics of phosphate linkages in RNA. Angew. Chem., Int. Ed..

[24] Iwase R., Toyama T., Nishimori K. (2007). Solid-phase synthesis of modified RNAs containing amide-linked oligoribonucleosides at their 3’-end and their application to siRNA. Nucleosides Nucleotides Nucleic Acids.

[25] Iwase R., Kurokawa R., Ueno J. (2009). Synthesis of modified double stranded RNAs containing duplex regions between amide-linked RNA and RNA at both ends and enhanced nuclease resistance. Nucleic Acids Symp. Ser..

[26] Gong W., Desaulniers J.-P. (2012). Gene-silencing properties of siRNAs that contain internal amide-bond linkages. Bioorg. Med. Chem. Lett..

[27] Potenza N., Moggio L., Milano G., Salvatore V., Di Blasio B., Russo A., Messere A. (2008). RNA interference in mammalia cells by RNA-3’-PNA chimeras. Int. J. Mol. Sci..

[28] Puglisi J.D., Tinoco I. (1989). Absorbance melting curves of RNA. Methods Enzymol..

[29] Berger I., Kang C., Sinha N., Wolters M., Rich A. (1996). A highly efficient 24-condition matrix for the crystallization of nucleic acid fragments. Acta Cryst. D.

[30] Kabsch W. (1993). Automatic processing of rotation diffraction data from crystals of initially unknown symmetry and cell constants. J. Appl. Cryst..

[31] Sheldrick G.M., Schneider T.R. (1997). SHELXL: high-resolution refinement. Methods Enzymol..

[32] Emsley P., Cowtan K. (2004). Coot: model-building tools for molecular graphics. Acta Cryst. D.

[33] Murshudov G.N., Vagin A.A., Dodson E.J. (1997). Refinement of macromolecular structures by the maximum-likelihood method. Acta Cryst. D.

[34] Bruenger A.T. (1992). Free R value: a novel statistical quantity for assessing the accuracy of crystal structures. Nature.

[35] Kim S.H., Shin W.C., Warrant R.W. (1985). Heavy metal ion-nucleic acid interaction. Methods Enzymol..

[36] Tanaka Y., Fujii S., Hiroaki H., Sakata T., Tanaka T., Uesugi S., Tomita K.-i., Kyogoku Y. (1999). A’-form RNA double helix in the single crystal structure of r(UGAGCUUCGGCUC). Nucleic Acids Res..

[37] Saenger W. (1984). Principles of Nucleic Acid Structure.

[38] Egli M., Portmann S., Usman N. (1996). RNA hydration: A detailed look. Biochemistry.

[39] Wang Y., Juranek S., Li H., Sheng G., Tuschl T., Patel D.J. (2008). Structure of an argonaute silencing complex with a seed-containing guide DNA and target RNA duplex. Nature.

[40] Ma J.B., Yuan Y.R., Meister G., Pei Y., Tuschl T., Patel D.J. (2005). Structural basis for 5’-end-specific recognition of guide RNA by the A. fulgidus Piwi protein. Nature.

[41] Parker J.S., Roe S.M., Barford D. (2005). Structural insights into mRNA recognition from a PIWI domain-siRNA guide complex. Nature.

[42] Ma J.B., Ye K., Patel D.J. (2004). Structural basis for overhang-specific small interfering RNA recognition by the PAZ domain. Nature.

[43] Sheng G., Zhao H., Wang J., Rao Y., Tian W., Swarts D.C., van der Oost J., Patel D.J., Wang Y. (2014). Structure-based cleavage mechanism of Thermus thermophilus Argonaute DNA guide strand-mediated DNA target cleavage. Proc. Natl. Acad. Sci. U. S. A..

[44] Frank F., Sonenberg N., Nagar B. (2010). Structural basis for 5’-nucleotide base-specific recognition of guide RNA by human AGO2. Nature.

[45] Chen P.Y., Weinmann L., Gaidatzis D., Pei Y., Zavolan M., Tuschl T., Meister G. (2008). Strand-specific 5’-O-methylation of siRNA duplexes controls guide strand selection and targeting specificity. RNA.

[46] Jackson A.L., Burchard J., Leake D., Reynolds A., Schelter J., Guo J., Johnson J.M., Lim L., Karpilow J., Nichols K. (2006). Position-specific chemical modification of siRNAs reduces “off-target” transcript silencing. RNA.

[47] Braasch D.A., Jensen S., Liu Y., Kaur K., Arar K., White M.A., Corey D.R. (2003). RNA interference in mammalian cells by chemically-modified RNA. Biochemistry.

[48] Amarzguioui M., Holen T., Babaie E., Prydz H. (2003). Tolerance for mutations and chemical modifications in a siRNA. Nucleic Acids Res..

[49] Harborth J., Elbashir S.M., Vandenburgh K., Manninga H., Scaringe S.A., Weber K., Tuschl T. (2003). Sequence, chemical, and structural variation of small interfering RNAs and short hairpin RNAs and the effect on mammalian gene silencing. Antisense Nucl. Acid Drug Dev..

[50] Yang X., Sierant M., Janicka M., Peczek L., Martinez C., Hassell T., Li N., Li X., Wang T., Nawrot B. (2012). Gene silencing activity of siRNA molecules containing phosphorodithioate substitutions. ACS Chem. Biol..

[51] Hall A.H.S., Wan J., Shaughnessy E.E., Ramsay Shaw B., Alexander K.A. (2004). RNA interference using boranophosphate siRNAs: structure-activity relationships. Nucleic Acids Res..

[52] Tanui P., Kennedy S.D., Lunstad B.D., Haas A., Leake D., Rozners E. (2014). Synthesis, biophysical studies and RNA interference activity of RNA having three consecutive amide linkages. Org. Biomol. Chem..

