# Erratum

**DOI:** 10.1093/nar/gku669

**Published:** 2014-09-02



Amides are excellent mimics of phosphate internucleoside linkages and are well tolerated in short interfering RNAs

Daniel Mutisya, Chelliah Selvam, Benjamin D. Lunstad, Pradeep S. Pallan, Amanda Haas, Devin Leake, Martin Egli, and Eriks Rozners Nucl. Acids Res. (2014) 42 (10): 6542-6551. doi: 10.1093/nar/gku235.

First published online: May 9, 2014. Corrected after print: June 2, 2014.

Due to an error in production, in the original version of this Article published on May 9, 2014, an oversized version of Figure 9 was inserted instead of Scheme 1. Scheme 1 was missing. These errors have now been corrected in the new online version of the Article and Scheme 1 is included below. The Publisher wishes to apologise to the Authors for this error.

**Scheme 1.**
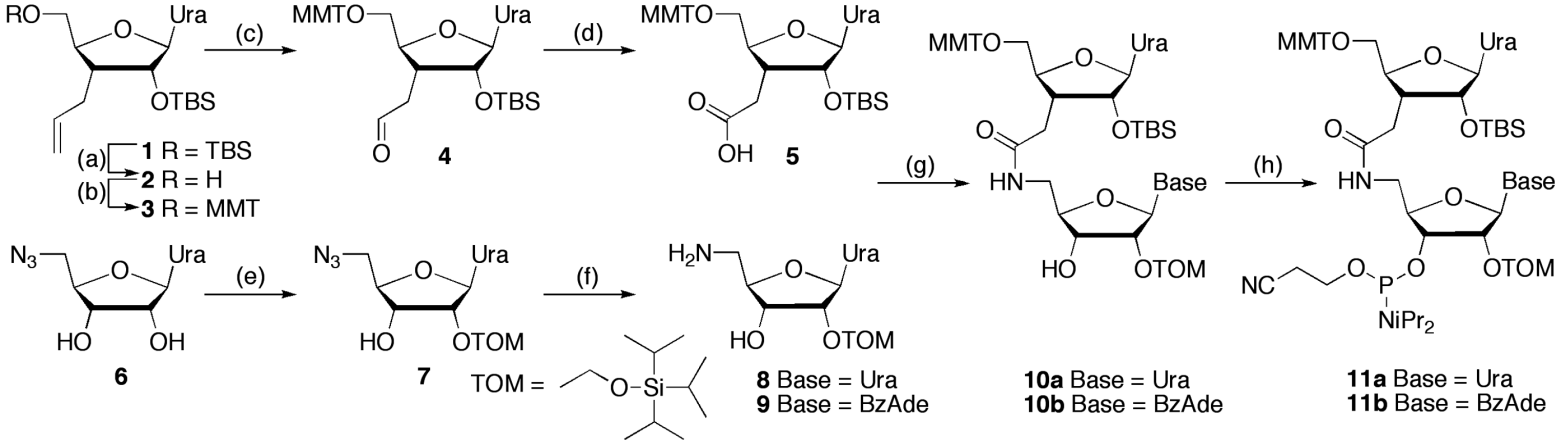
Synthesis of dimeric r(U_AM1_U) and r(U_AM1_A) phosphoramidites.*^a^* *^a^*Steps: (a) TFA, THF, H_2_O 0 °C, 4 h, 92%; (b) p-methoxytrityl chloride, pyridine, 0 °C to rt, 14 h, 85%; (c) OsO_4_, 4-methylmorpholine N-oxide, dioxane, rt, 10 h, then NaIO_4_ in water, rt, 12 h, 95%; (d) NaClO_4_, NaH_2_PO_4_, 2-methylbut-2-ene, *t*-BuOH, THF water, rt, 1 h, 82%; (e) DIEA, Bu_2_SnCl_2_, dichloroethane, rt, 1 h, then add TOM-Cl, 80 °C, 45 min, 39%; (f) H_2_S, pyridine, water, rt, 14 h 93%; (g) HBTU, HOBt, DIEA, CH_2_Cl_2_, rt, 12 h, 95% **11a**, 86% **11b**; (h) DIEA, ClP(OCH_2_CH_2_CN)N(iPr)_2_, CH_2_Cl_2_, rt, 7 h, 71% **11a**, 60% **11b**.

