# Supplementary material for: Amides are excellent mimics of phosphate internucleoside linkages and are well tolerated in short interfering RNAs
Source: Nucleic Acids Res. 2014 May 9;42(10):6542–51. doi: 10.1093/nar/gku235 (PMC4041415; doi:10.1093/nar/gku235)
Supplement: SUPPLEMENTARY DATA [file supp_42_10_6542__index.html]

Amides are excellent mimics of phosphate internucleoside linkages and are well tolerated in short interfering RNAs — Amides are excellent mimics of phosphate internucleoside linkages and are well tolerated in short interfering RNAs — SUPPLEMENTARY DATA 

# Amides are excellent mimics of phosphate internucleoside linkages and are well tolerated in short interfering RNAs

## SUPPLEMENTARY DATA

**Files in this Data Supplement:**

- SUPPLEMENTARY DATA
