# Supplementary material for: Amides are excellent mimics of phosphate internucleoside linkages and are well tolerated in short interfering RNAs
Source: Nucleic Acids Res. 2014 May 9;42(10):6542–51. doi: 10.1093/nar/gku235 (PMC4041415; doi:10.1093/nar/gku235)
Supplement: SUPPLEMENTARY DATA [file supp_gku235_nar-03689-y-2013-File012.pdf]

SUPPLEMENTARY DATA

Amides Are Excellent Mimics of Phosphate Internucleoside Linkages and Are Well Tolerated in Short Interfering RNAs

Daniel Mutisya,[1] Chelliah Selvam,[1] Benjamin D. Lunstad,[2] Pradeep S. Pallan,[3] Amanda Haas,[2] Devin Leake,[2] Martin Egli[3,]* and Eriks Rozners[1,]*

[1]Department of Chemistry, Binghamton University, The State University of New York, Binghamton, New York 13902.

[2]Global Research and Development in Molecular Biology, Thermo Fisher Scientific Bioscience Division, Lafayette, Colorado 80026.

[3]Department of Biochemistry, School of Medicine, Vanderbilt University, Nashville, Tennessee 37232.

Table of contents

Experimental procedures for synthesis of amide-modified RNA	Pg. S2
HPLC trace and CD spectra of **RNA13**	Pg. 8
Crystal structure determination methodology and results	Pg. S9
Cell viability and additional siRNA activity data	Pg. S11
HPLC analysis of amide-modified RNA	Pg. S16
Copies of ^{1}H, ^{13}C, and ^{31}P NMR spectra	Pg. S24

Experimental Procedures

Methylene chloride, tetrahydrofuran and toluene were purified by MBRAUN (MB SPS) solvent purification system, fitted with double filter columns. Pyridine, acetonitrile, and dimethylformamide were dried by refluxing with calcium hydride (CaH_2) followed by distillation and stored over activated molecular sieves. Reactions were carried out in oven (150 °C) dried glassware under an atmosphere of dry nitrogen. NMR spectra were recorded either on an Bruker Avance III 600 MHz or Bruker AM360 MHz NMR spectrometers operating at ambient temperature. Reverse-phase high pressure liquid chromatography (HPLC) purification was carried out on Shimadzu SPD-10A or SPD-20A systems fitted with analytical XBridge™ C18 3.5µm, 4.6 x 150 mm or semi-preperative C18 5µm, 10 x 150 mm columns respectively. Analytical thin layer chromatography (TLC) was performed on SiliCycle 0.25 mm 60Å silica gel F254 plates, while the preparative thin layer chromatography was performed on SiliCycle 2000µm 60Å silica gel F254; 20cm x 20cm plates. Column chromatography was done on SiliaFlash® P60 230-400 mesh silica gel (SiliCycle).

3′-Allyl-3′-deoxy-2′-*O*-*tert*-butyldimethylsilyluridine (2) A mixture of TFA and H_2O (24 mL, 1:1 v/v) was cooled to 0 ºC and slowly added to a cooled (0 ºC) solution of 3′-allyl-3′-deoxy-2′,5′-*O*-bis(*ter*-butyldimethylsilyl)uridine **1**[1] (1.00 g, 2.01 mmol) in THF (14 mL). The reaction mixture was stirred at 0 ˚C for 4 hours and was quenched by addition of saturated aqueous solution of $NaHCO_3$ (30 mL). The mixture was extracted with ethyl acetate (30 mL). The organic phase was separated, washed with water (10 mL), brine (10 mL), dried over anhydrous Na_2SO_4 and concentrated under reduced pressure. The crude residue was purified using flash silica gel chromatography (0-5% methanol in dichloromethane) affording compound **2** as white solid. Yield: 0.704 g, 92%. R_f = 0.3 (4 % methanol in dichloromethane). ^{1}H NMR ($CDCl_3$, 600 MHz) δ: 9.56 (s, 1H), 8.20 (d, *J* = 12.0 Hz, 1H), 5.79-5.74 (m, 1H), 5.69 (d, *J* = 12.0 Hz, 1H), 5.64 (s, 1H), 5.12-5.09 (m, 1H), 5.06-5.04 (m, 1H), 4.33 (d, *J* = 6.0 Hz, 1H), 4.14-4.10 (m, 2H), 3.81 (dd, *J* = 12.0, 6.0 Hz, 1H), 2.39-2.34 (m, 1H), 2.17-2.15 (m, 1H), 2.14-2.06 (m, 1H), 0.92 (s, 9H), 0.24 (s, 3H), and 0.12 (s, 3H). ^{13}C NMR ($CDCl_3$, 150 MHz) δ: 164.6, 150.5, 141.6, 135.9, 117.0, 101.1, 92.3, 85.4, 77.8, 61.2, 40.6, 29.0, 26.1, 18.3, -4.1 and -5.2. MS (ESI) calculated for $C_{18}H_{30}N_2O_5Si$, [M + H] 383.53, found 383.5

3′-Allyl-3′-deoxy-5′-*O*-methoxytrityl-2′-*O*-*ter*-butyldimethylsilyluridine (3) 3′-Allyl-3′-deoxy-2′-*O*-ter-butyldimethylsilyluridine **2** (0.47 g, 1.22 mmol) was co-evaporated in dry pyridine (3 × 10 mL), dissolved in dry pyridine (10 mL) and cooled to 0 ˚C under the nitrogen atmosphere. p-Methoxytritylchloride (0.452 g, 1.46 mmol) was slowly added and the reaction mixture was left stirring under the melting ice for 14 hours

[1] Rozners, E., Katkevica, D., Bizdena, E. and Strömberg, R. (2003) Synthesis and Properties of RNA Analogs Having Amides as Interuridyl Linkages at Selected Positions. *J. Am. Chem. Soc.*, **125**, 12125-12136; Selvam, C., Thomas, S., Abbott, J., Kennedy, S.D. and Rozners, E. (2011) Amides as Excellent Mimics of Phosphate Linkages in RNA. *Angew. Chem., Int. Ed.*, **50**, 2068-2070.

until the reaction was complete as judged by TLC. The reaction mixture was quenched with methanol (1 mL), evaporated to near dryness, dissolved in dichloromethane (30 mL) and extracted with saturated $NaHCO_3$ (20 mL). The organic layer was separated, dried with anhydrous Na_2SO_4, and concentrated under reduced pressure. The crude residue was purified using flash silica gel chromatography (0-3% methanol in dichloromethane) affording compound **3**, as white foam. Yield: 0.679 g, 85%. R_f = 0.3 (20% ethyl acetate in dichloromethane). ^{1}H NMR ($CDCl_3$, 600 MHz) δ: 8.60 (s, 1H), 8.23 (d, *J* = 8.4 Hz, 1H), 7.38-7.23 (m, 12H), 6.83 (d, *J* = 6.6 Hz, 2H), 5.69-5.62 (m, 1H), 5.66 (s, 1H), 5.20 (d, *J* = 8.4 Hz, 1H), 5.00 (dd, *J* = 1.8, 16.8 Hz, 1H), 4.90 (dd, *J* = 1.2, 10.2 Hz, 1H), 4.25 (d, *J* = 3 Hz, 1H), 4.08 (m, 1H), 3.77 (s, 3H), 3.68 (dd, *J* = 1.8, 11.4 Hz, 1H), 3.30 (dd, *J* = 3.0, 11.4 Hz, 1H), 2.28-2.24 (m, 2H), 1.87 (m, 1H), 0.87 (s, 9H), 0.22 (s, 3H), and 0.09 (s, 3H). ^{13}C NMR ($CDCl_3$, 150 MHz) δ: 163.5, 159.1, 150.3, 143.8, 140.8, 135.8, 134.8, 130.7, 128.7, 128.2, 127.5, 116.8, 113.5, 101.4, 91.7, 87.4, 84.3, 78.1, 61.7, 55.5, 41.5, 28.8, 26.1, 18.3, -4.0, and -5.3.

3′-carboxymethyl-3′-deoxy-5′-*O*-methoxytrityl-2′-*O*-*ter*-butyldimethylsilyluridine (4) 3′-Allyl-3′-deoxy-5′-*O*-methoxytrityl-2′-*O*-*tert*-butyldimethylsilyluridine **3** (1.60 g, 2.44 mmol) and *N*-methyl-morpholine-*N*-oxide (0.6 mL, 2.69 mmol) were dissolved in dioxane (10 mL). Osmium tetraoxide (0.4 mL of 4% aqueous solution, 0.049 mmol) was added and the reaction mixture was protected from sunlight and stirred for 10 hours. The reaction was quenched with dichloromethane (20 mL) and extracted in saturated $NaHCO_3$ (20 mL). The organic layer was separated, dried with Na_2SO_4 and evaporated. The crude residue was re-dissolved in dioxane (10 mL), $NaIO_4$ (0.576 g, 2.69 mmol in 0.9 mL of water) was added and the reaction mixture was stirred for 12 hours. Upon the completion, the reaction was diluted with dichloromethane (20 mL) and extracted in aqueous $NaHCO_3$ (20 mL). The organic layer was separated, dried with anhydrous Na_2SO_4 and concentrated under reduced pressure. The crude residue was purified using flash silica gel chromatography (0-5% methanol in dichloromethane) to afford aldehyde **4** as white solid. Yield: 1.523 g, 95%. R_f = 0.3 (5% methanol in dichloromethane). ^{1}H NMR ($CDCl_3$, 600 MHz) δ: 9.65 (s, 1H), 8.57 (s, 1H), 8.16 (d, *J* = 8.4 Hz, 1H), 7.38-7.23 (m, 12H), 6.82 (d, *J* = 9 Hz, 2H), 5.71 (s, 1H), 5.31(dd, *J* = 2.4, 8.4 Hz, 1H), 4.43 (d, *J* = 3.6 Hz, 1H), 4.00 (m, 1H), 3.77 (s, 3H), 3.77-3.75 (m, 1H), 3.25 (dd, *J* = 3.0, 12.0 Hz, 1H), 2.65-2.59 (m, 2H), 2.00-1.98 (m, 1H), 0.83 (s, 9H), 0.20 (s, 3H), and 0.00 (s, 3H). ^{13}C NMR ($CDCl_3$, 150 MHz) δ: 199.7, 163.3, 159.1, 150.3, 143.8, 140.5, 134.7, 130.7, 128.6, 128.3, 127.6, 113.6, 101.8, 91.9, 87.7, 83.6, 77.6, 61.2, 55.5, 39.1, 36.1, 26.0, 18.3, -4.2, and -5.4.

3′-carboxylmethyl-3′-deoxy-5′-*O*-methoxytrityl-2′-*O*-*ter*-butyldimethylsilyluridine (5) 3′-carboxy-methyl-3′-deoxy-5′-*O*-methoxytrityl-2′-*O*-*ter*-butyldimethylsilyluridine **4** (0.627 g, 0.95 mmol) was

dissoleved in a mixture of *tert*-butanol and water (8.4 mL, 1:1 v/v) and a 2 M solution of 2-methylbut-2-ene in THF (2.4 mL) was added. Monobasic sodium phosphate (NaH_2PO_4, 0.287 g, 2.39 mmol) was added followed by sodium chlorite ($NaClO_2$, 0.301 g, 3.33 mmol). The reaction mixture was stirred at room temperature for 1 hour after which, it was quenched with saturated aqueous solution of sodium thiosulfate ($Na_2S_2O_2$, 5 mL) and the mixture was concentrated by freeze-drying overnight. The crude residue was purified using flash silica gel chromatography (0-5 % methanol in dichloromethane) affording carboxylic acid **5** as white powder. Yield: 0.524 g, 82%. R_f = 0.2 (5% methanol in dichloromethane). ^1H NMR ($CDCl_3$, 600 MHz) δ: 9.44 (s, 1H), 8.16 (d, *J* = 8.4Hz, 1H), 7.36-7.17 (m, 12H), 6.79 (d, *J* = 9 Hz, 2H), 5.66 (s, 1H), 5.28 (dd, *J* = 1.2, 8.4 Hz, 1H), 4.40 (d, *J* = 3 Hz, 1H), 3.98-3.96 (m, 1H), 3.72 (s, 3H), 3.69 (m, 1H), 3.25 (dd, *J* = 3.0, 12.0 Hz, 1H), 2.50-2.45 (m, 2H), 1.92-1.90 (m, 1H), 0.81 (s, 9H), 0.17 (s, 3H), and 0.00 (s, 3H). ^{13}C NMR ($CDCl_3$, 150 MHz) δ: 176.1, 164.1, 158.9, 150.3, 143.7, 140.7, 134.5, 130.5, 128.4, 128.1, 127.4, 113.4, 101.5, 91.8, 87.5, 83.2, 77.5, 61.0, 55.3, 37.9, 28.6, 25.8, 18.1, -4.4, and -5.7. MS (ESI) calculated for $C_{35}H_{44}N_2O_8Si$ [M + Na] 695.83 found 695.5.

5′-azido-5′-deoxy-3′-*O*-triisopropylsilyloxymethyluridine (7) Diisopropylethylamine (5.5 mL, 31.8 mmol) was added to the solution of 5′-azido-5′-deoxy-uridine **6** (2.5 g, 9.3 mmol) in dichloroethane (86 mL). Bu_2SnCl_2 (3.2 g, 10.5 mmol) was added and the reaction mixture was stirred for 1 h at room temperature. To the resulting white suspension was added (triisopropylsiloxy)methyl chloride (2.6 mL, 11.2 mmol) and the mixture was heated to 80 °C and stirred at this temperature for 45 min. The reaction mixture was cooled to room temperature and concentrated under reduced pressure. The residue was dissolved in dichloromethane (50 mL) and extracted with water (40 mL). The organic layer was separated, dried over anhydrous Na_2SO_4 and concentrated under reduced pressure. The crude residue was purified using flash silica gel chromatography using 20-50% ethyl acetate in hexane (containing 1% triethylamine) as eluent to afford the desired 2′-*O*-TOM isomer **7** as white foam. Yield: 1.65 g, 39%. R_f = 0.4 (40% ethyl acetate in hexanes). ^1H NMR (CD_3CN, 600MHz) δ: 9.16 (s, 1H), 7.53 (d, *J* = 8.4 Hz, 1H), 5.88 (d, *J* = 5.4 Hz, 1H), 5.66 (d, *J* = 8.4 Hz, 1H), 5.00 (dd, *J* = 5.4, 7.2 Hz, 2H), 4.33 (t, *J* = 5.4 Hz, 1H), 4.12 (brs, 1H), 4.03-4.01 (m, 1H), 3.65 (dd, *J* = 3.6, 13.8 Hz, 2H), 3.37 (brs, 1H) and 1.11-1.05 (m, 3H), 1.03 (d, *J* = 6.6 Hz, 18H). ^{13}C NMR (CD_3CN, 150 MHz) δ: 163.9, 151.5, 141.7, 103.5, 90.7, 88.8, 83.8, 79.7, 71.0, 53.0, 18.2 and 12.8. The connectivity of the (triisopropylsiloxy)methyl unit at the 2′-oxygen was confirmed by the HMBC experiment that showed a ^3J correlation between the methylenic protons of the (triisopropylsiloxy)methyl unit at δ_H 5.00 and the C-2′ (δ_C 88.8), and the methine proton H-2′ at δ_H 4.33 and the methylenic carbon at δ_C 90.7 of the (triisopropylsiloxy)methyl unit.

5′-amino-5′-deoxy-3′-*O*-triisopropylsilyloxymethyluridine (8) Hydrogen sulfide (generated *in situ* by reaction of iron(II) sulfide with 75% sulfuric acid solution) was passed through a well-stirred solution of 5′-azido-5′-deoxy-3′-*O*-(triisopropylsilyloxy)methyluridine **7** (0.320 g, 0.70 mmol) in pyridine (36 mL) and water (9 mL) for one hour. The reaction mixture was then stirred overnight at room temperature, volatiles were removed under vacuum and the crude residue was purified using flash silica gel chromatography (3-10% methanol in dichloromethane containing 1% aqueous ammonia solution) affording amine **8** as white solid. Yield: 0.280 g, 93%. R_f = 0.2 (5 % methanol in dichloromethane). ^{1}H NMR ($CDCl_3$, 600 MHz) δ: 7.74 (d, *J* = 7.8 Hz, 1H), 5.86 (d, *J* = 3.6 Hz, 1H), 5.74 (d, *J* = 7.8 Hz, 1H), 5.17 (d, *J* = 4.8 Hz, 1H), 5.00 (d, *J* = 4.8 Hz, 1H), 4.31 (dd, *J* = 3.6, 5.4 Hz, 1H), 4.12 (t, *J* = 6.0 Hz, 1H), 3.96 (dd, *J* = 4.8, 9.0 Hz, 1H), 3.14 (dd, *J* = 3.6, 13.8 Hz, 1H), 3.00 (dd, *J* = 3.6, 13.8 Hz, 1H) and 1.00-0.87 (m, 21H). ^{13}C NMR ($CDCl_3$, 150 MHz) δ: 163.3, 150.3, 141.1, 102.7, 90.9, 89.9, 85.1, 82.2, 70.2, 42.9, 18.0 and 12.1. MS (ESI) calculated for $C_{19}H_{35}N_3O_6Si$ [M + H] 429.58, found 430.4.

Dimer 10a. Carboxylic acid **5** (0.397 g, 0.590 mmol) and 1-hydroxybenzotriazole (HOBt, 0.088 g, 0.649 mmol) were co-evaporated with dry acetonitrile (3 × 10 mL) and dissolved in dry dichloromethane (9 mL). *O*-Benzotriazole-N,N,N′,N′-tetramethyluroniumhexafluorophosphate (HBTU, 0.268 g, 0.708 mmol) and diisopropylethylamine (0.2 mL, 1.03 mmol) were added. The reaction was stirred under nitrogen for 5 min. A solution of aminouridine **8** (0.254 g, 0.590 mmol) in dichloromethane (4 mL) was added. The reaction mixture was stirred at room temperature for 12 hours, and then concentrated under reduced pressure to remove dichloromethane. The crude product was purified by silica gel column chromatography using 2-10% methanol in dichloromethane to give dimer **10a** as a colorless solid. Yield: 0.608 g, 95%. R_f = 0.3 (5% methanol in dichloromethane). ^{1}H NMR (CD_3CN, 600 MHz) δ: 9.15 (brs, 2H), 7.94 (d, *J* = 8.4 Hz, 1H), 7.45-7.26 (m, 13H), 6.89 (d, *J* = 6.0 Hz, 2H), 5.77 (d, *J* = 5.4 Hz, 1H), 5.63 (brs, 1H), 5.61 (d, *J* = 8.4 Hz, 1H), 5.16 (d, *J* = 7.8 Hz, 1H), 4.99 (s, 2H), 4.44 (d, *J* = 4.8 Hz, 1H), 4.32 (t, *J* = 5.4 Hz, 1H), 4.01-4.00 (m, 2H), 3.90-3.88 (m, 1H), 3.77 (s, 3H), 3.51 (dd, *J* = 2.4, 11.4 Hz, 1H), 3.39-3.33 (m, 3H), 2.65 (m, 1H), 2.39 (m, 1H), 2.01 (m, 1H), 1.02 (m, 21H), 0.89 (s, 9H), 0.17 (s, 3H), and 0.06 (s, 3H). ^{13}C NMR (CD_3CN, 150 MHz) δ: 172.1, 164.2, 163.9, 160.0, 151.5, 145.4, 142.4, 141.4, 136.0, 131.6, 129.5, 129.4, 129.1, 128.3, 114.3, 103.2, 101.9, 92.4, 90.7, 89.8, 88.1, 84.3, 84.0, 79.7, 78.9, 71.4, 63.3, 56.2, 42.0, 39.5, 31.6, 26.4, 18.8, 18.3, 12.8, -4.1, and -5.0. MS (ESI) calculated for $C_{56}H_{77}N_5O_{13}Si_2$ [M + Na] 1107.4, found 1106.9.

Dimer 10b. Carboxylic acid **5** (0.157 g, 0.233 mmol) and (HOBt, 0.039 g, 0.257 mmol) were co-evaporated with dry acetonitrile (3 × 8 mL) and dissolved in dry dichloromethane (4 mL). HBTU (0.11 g, 0.280 mmol) and diisopropylethylamine (0.1 mL, 0.494 mmol) were added. The reaction mixture was stirred under

nitrogen for 5 min. A solution of aminoadenosine **9**[2] (0.130 g, 0.233 mmol) in dichloromethane (2 mL) was added dropwise. The reaction mixture was stirred at room temperature for 12 hours, and then concentrated under reduced pressure to remove dichloromethane. The crude product was purified by silica gel column chromatography eluting with 2-5% methanol in dichloromethane to give dimer **10b** as a colorless solid. Yield: 0.243 g, 86 %. R_f = 0.3 (5% methanol in dichloromethane). ^{1}H NMR (CD_3CN, 600 MHz) δ: 9.45 (brs, 1H), 9.25 (brs, 1H), 8.58 (s, 1H), 8.27 (s, 1H), 8.01 (d, *J* = 7.2 Hz, 2H), 7.95 (d, *J* = 8.4 Hz, 1H), 7.74-7.10 (m, 15H), 6.85 (d, *J* = 9 Hz, 2H), 6.06 (d, *J* = 6 Hz, 1H), 5.64 (s, 1H), 5.16 (d, *J* = 7.8 Hz, 1H), 5.02 (m, 1H), 4.93 (m, 2H), 4.48 (d, *J* = 4.8 Hz, 1H), 4.29 (m, 1H), 4.15 (m, 1H), 4.04-4.01 (m, 1H), 3.75 (s, 3H), 3.63 (m, 1H), 3.54 (dd, *J* = 1.8, 11.4 Hz, 1H), 3.41 (m, 1H), 3.33 (dd, *J* = 3.6, 11.4 Hz, 1H), 2.66-2.64 (m, 1H), 2.48-2.44 (m, 1H), 2.00 (m, 1H), 0.88 (m, 21H), 0.85 (s, 9H), 0.16 (s, 3H) and 0.03 (s, 3H). ^{13}C NMR (CD_3CN, 150 MHz) δ: 172.0, 164.2, 163.3, 160.0, 153.1, 151.5, 145.4, 145.3, 144.5, 141.4, 135.9, 133.7, 131.6, 129.8, 129.5, 129.4, 129.3, 129.1, 128.3, 128.2, 126.3, 114.3, 102.0, 92.4, 90.7, 89.0, 88.1, 85.3, 84.3, 79.6, 78.9, 71.7, 63.2, 56.1, 42.1, 39.5, 31.7, 26.3, 18.8, 18.2, 12.7, -4.2 and -4.9. MS (ESI) calculated for $C_{64}H_{82}N_8O_{12}Si_2$ [M + H] 1212.6, found 1212.0.

Phosphoramidite 11a. Dimer **10a** (0.400 g, 0.37 mmol) was coevaporated with dry acetonitrile (3 × 8 mL) and dissolved in dry dichloromethane (5 mL). Diisopropylethylamine (0.18 mL, 1.11 mmol) and chloro-2-cyanoethyl-*N*,*N*-diisopropylphosphoramidite (0.12 mL, 0.55 mmol) were carefully added to the reaction mixture. After stirring for 7 hours at room temperature, the reaction mixture was concentrated under reduced pressure. The residue was applied onto a silica gel column (prepared in hexane/ethyl, 1:1, v/v, containing 3% triethylamine) and eluted with 0-7% methanol in hexane/ethyl acetate mixture (1:1, v/v) containing 2% triethylamine to afford phosphoramidite dimer **11a** as white foam. Yield: 0.337 g, 71%. R_f = 0.3 (3 % methanol in ethyl acetate/hexane, v/v; 1:1). ^{1}H NMR (CD_3CN, 600 MHz) δ: 7.91 (d, *J* = 8.4 Hz, 1H), 7.44-7.26 (m, 13H), 6.88 (d, *J* = 9.0 Hz, 2H), 5.85 (d, *J* = 4.2 Hz, 1H), 5.64-5.60 (m, 2H), 5.17 (d, *J* = 8.4 Hz, 1H), 5.00 (dd, *J* = 4.8, 15.0 Hz, 1H), 4.90 (dd, *J* = 4.8, 15.0 Hz, 1H), 4.47-4.42 (m, 2H), 4.27 (m, 1H), 4.01-3.99 (m, 2H), 3.84-3.80 (m, 2H), 3.77 (s, 3H), 3.64-3.61 (m, 2H), 3.51-3.45 (m, 2H), 3.42-3.25 (m, 2H), 2.72-2.61 (m, 3H), 2.45-2.33 (m, 1H), 2.10-1.99 (m, 1H), 1.19-1.14 (m, 12H), 1.02-0.99 (m, 21H), 0.89 (s, 9H), 0.17 (s, 3H) and 0.06 (s, 3H). ^{31}P NMR (CD_3CN) δ: 150.1 and 149.7. MS (ESI) calculated for $C_{65}H_{94}N_7O_{14}PSi_2$ [M] 1284.6, found 1284.5.

[2] Selvam, C., Thomas, S., Abbott, J., Kennedy, S.D. and Rozners, E. (2011) Amides as Excellent Mimics of Phosphate Linkages in RNA. *Angew. Chem., Int. Ed.*, **50**, 2068-2070.

Phosphoramidite 11b. Dimer **10b** (0.270 g, 0.222 mmol) was coevaporated with dry acetonitrile (3 × 8 mL) and dissolved in dry dichloromethane (3 mL). Diisopropylethylamine (0.12 mL, 0.67 mmol) and chloro-2-cyanoethyl-*N*,*N*-diisopropylphosphoramidite (0.074 mL, 0.33 mmol) were carefully added to the reaction mixture. After stirring for 7 hours at room temperature and under nitrogen atmosphere, the reaction mixture was concentrated under reduced pressure. The crude residue was applied onto a silica gel column (prepared in hexane/ethyl, 1:1, v/v, containing 3% triethylamine) and eluted with 0-7% methanol in hexane/ethyl acetate (1:1, v/v) containing 2% triethylamine to afford phosphoramidite dimer **11b** as a white foam. Yield: 0.188 g, 60%. R_f = 0.25 (4 % methanol in ethyl acetate/hexane (v/v; 1:1). ^{1}H NMR (CD_3CN, 600 MHz) δ: 8.56 (s, 1H), 8.27 (s, 1H), 8.02-7.94 (m, 3H), 7.64 (m, 1H), 7.54 (m, 2H), 7.42 (m, 3H), 7.30-7.21 (m, 9H), 6.84 (m, 2H), 6.07 (d, *J* = 7.2 Hz, 1H), 5.64 (s, 1H), 5.28-5.26 (m, 1H), 5.15 (m, 1H), 4.97-4.93 (m, 1H), 4.86 (d, J = 4.8 Hz, 1H), 4.51 (m, 2H), 4.02 (m, 1H), 3.94-3.83 (m, 2H), 3.70-3.63 (m, 6H), 3.55-3.47 (m, 1H), 3.34-3.31 (m, 1H), 2.77 (m, 1H), 2.71-2.64 (m, 3H), 2.48-2.46 (m, 1H), 1.97-1.93 (m, 1H), 1.25 (m, 12H), 0.86 (s, 9H), 0.82-0.80 (m, 21H), 0.16 (s, 3H) and 0.05 (s, 3H). ^{31}P NMR (CD_3CN) δ: 150.5 and 149.8. MS (ESI) calculated for $C_{73}H_{99}N_{10}O_{13}PSi_2$, [M + H_2O] 1429.7, found 1429.7.

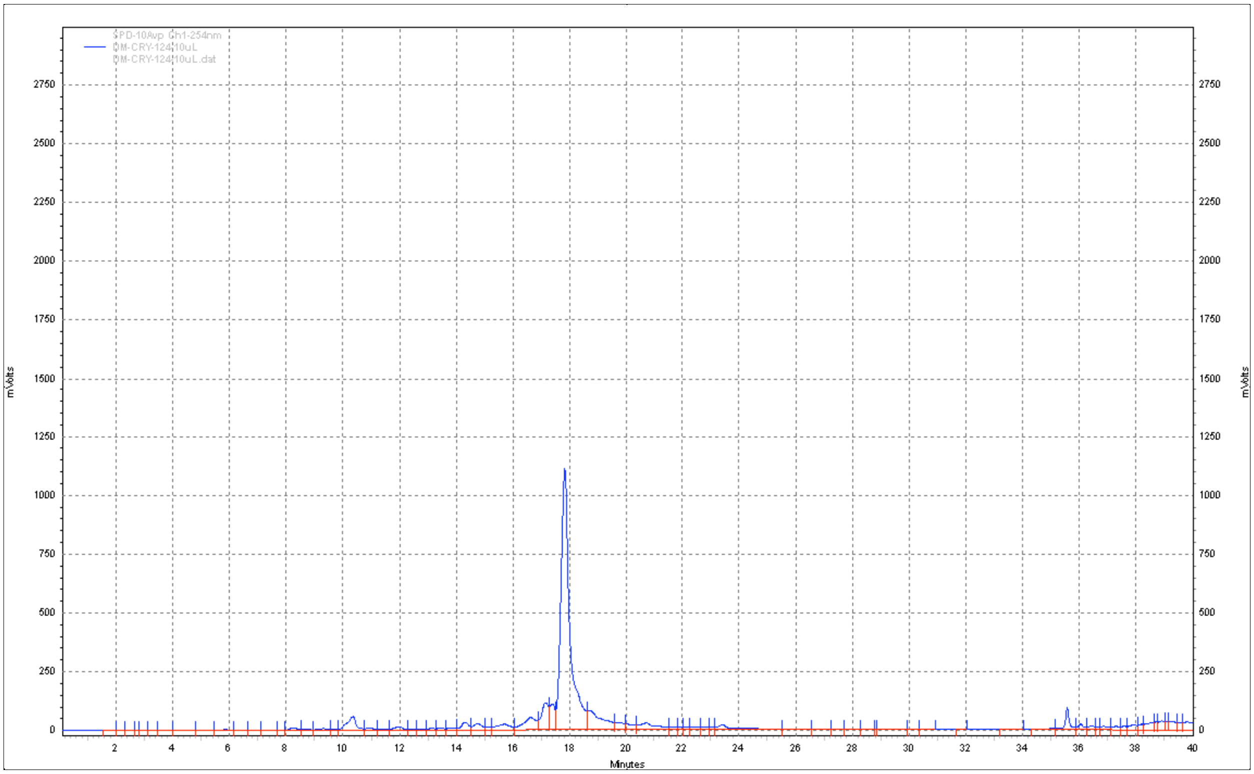

Figure S1. RP HPLC trace (XBridge™ C18 column, 4.6 × 150 mm) of crude **RNA13**; 388 nmols (yield 39 %) were isolated from the major peak.

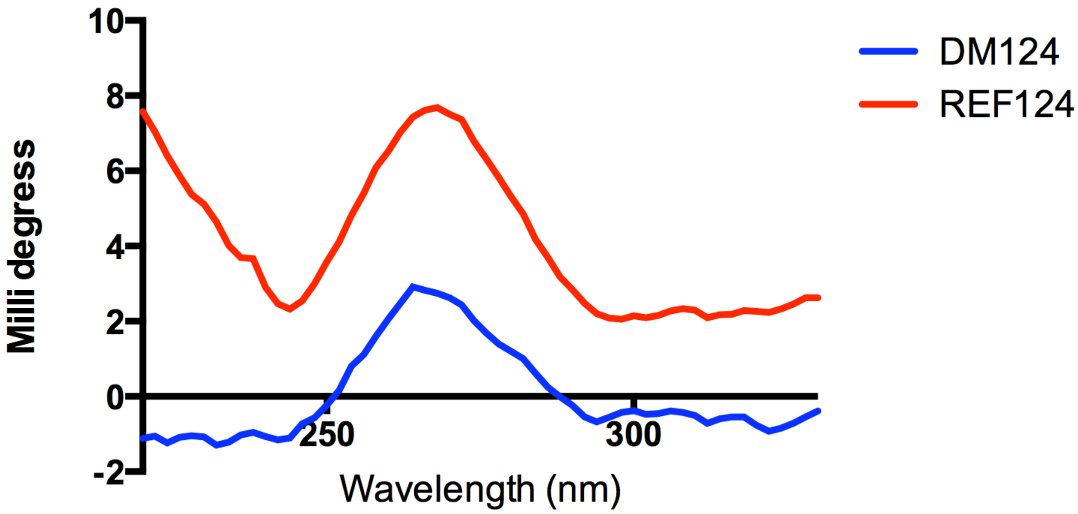

Figure S2. CD spectra of **RNA13** (blue line) and the unmodified RNA (red line). CD measurements were performed at 2 µM in oligonucleotides in a buffer containing 15 mM sodium phosphate (pH 7.2), 25 mM NaCl and 1 mM EDTA

Table S1. Selected Crystal and Refinement Data.

**Oligoribonucleotide**	5′-r(UpGpApGpCp**UaU**pCpGpGpCpUC)-3′
Space group	P1
Unit cell constants *a*, *b*, *c* [Å]; α, β, γ [°]	22.36, 34.33, 43.30; 110.03, 94.24, 95.83
Resolution [Å]	1.20
Outer shell [Å]	1.40-1.20
No. of unique reflections	35886
Completeness (outer shell) [%]	94.8 (add outer shell)
R-merge (outer shell) [%]	0.082 (0.32)
R-work [%]	0.171
R-free [%]	0.235
No. of RNA atoms	1057
No. of ions (Sr^{2+})/waters	2/269
R.m.s. deviations bonds [Å]	0.018
R.m.s. deviations angles [Å]	0.03
Avg. B-factor, DNA atoms [$Å^2$]	9
Avg. B-factor, solvent [$Å^2$]	22
PDB entry code	123X

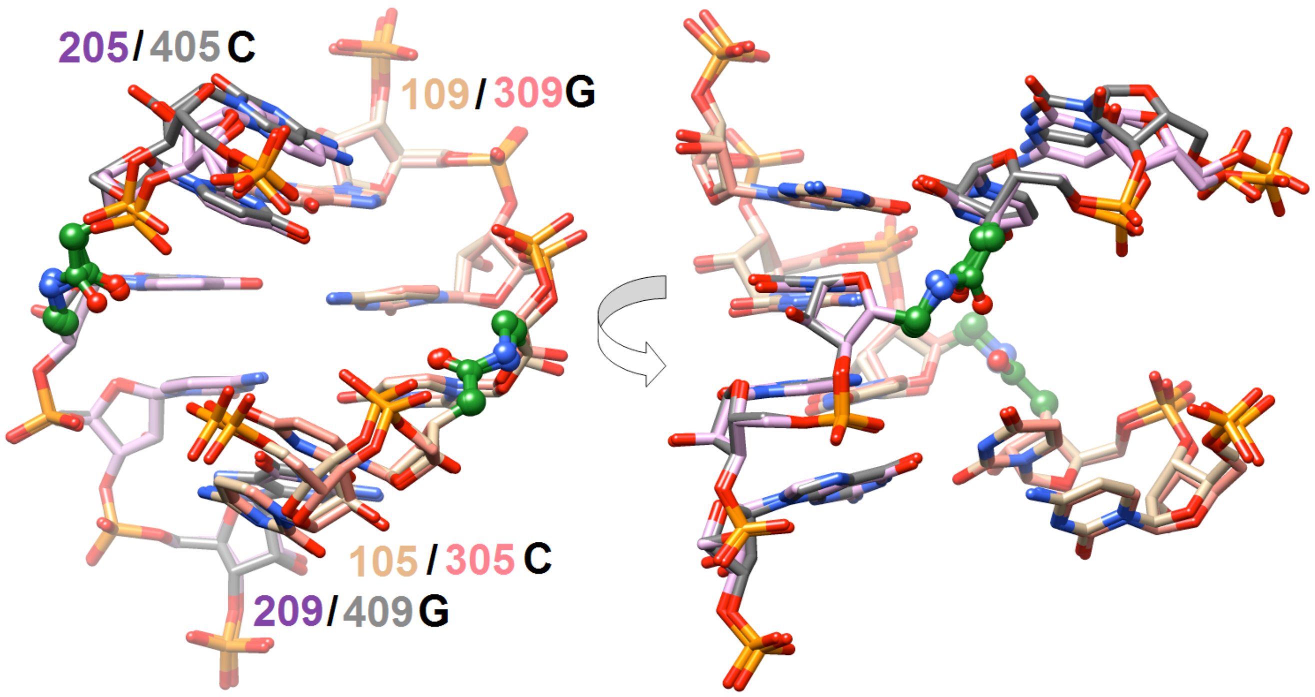

Figure S3. Comparison of the conformations of the central portions of the **RNA1** duplexes.

Resazurin (Alamar Blue) cell viability assay[3]: Resazurin (Acros Organics cat#189900010) at a final concentration of 125ug/mL was sterile filter with a Corning 0.2uM filter. 25 µL of Resazurin was then added to cell culture wells containing cells and 100 µL of cell media. Plates were incubate at 37ºC, 5% CO2 for 1-2 hours. The resulting fluorescence is read on a Wallac plate reader. Finally, results are analyzed by plotting fluorescence intensity (or absorbance) versus compound concentration.

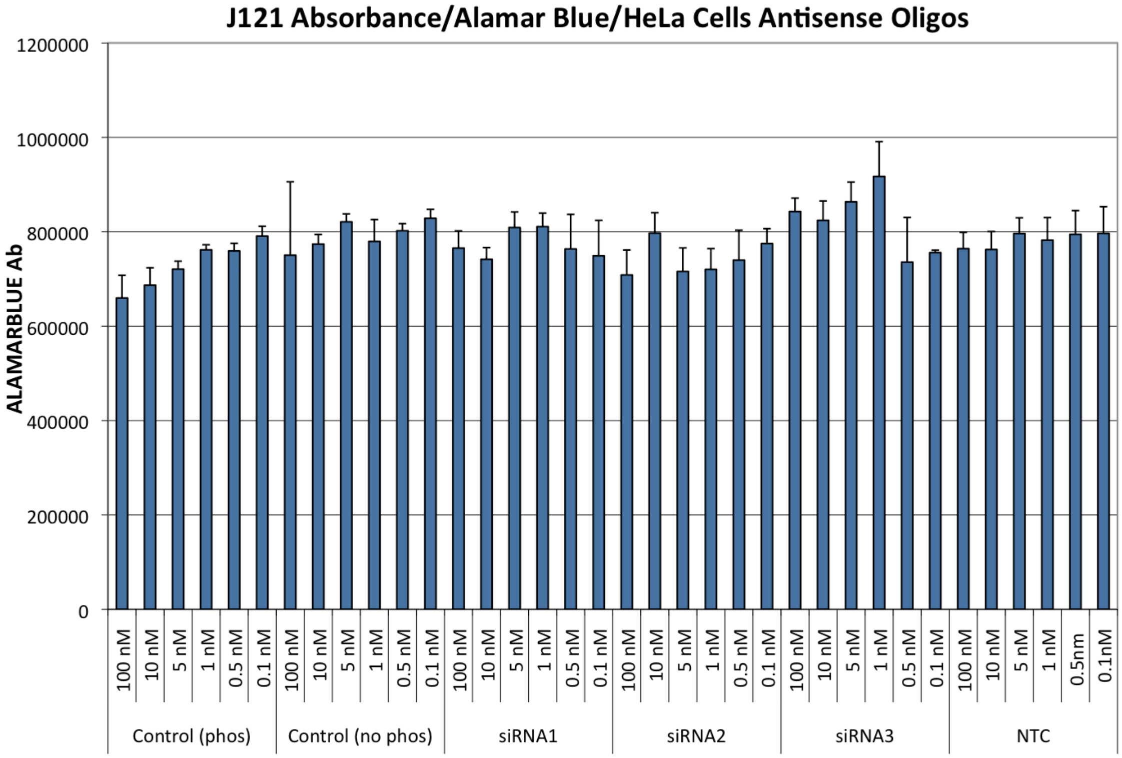

Figure S4. Cell viability for experiments shown in Figure 5 determined using a Resazurin assay.

[3] O'Brien, J., Wilson, I., Orton, T. and Pognan, F. (2000), Investigation of the Alamar Blue (resazurin) fluorescent dye for the assessment of mammalian cell cytotoxicity. *Eur. J. Biochem.*, **267**, 5421–5426.

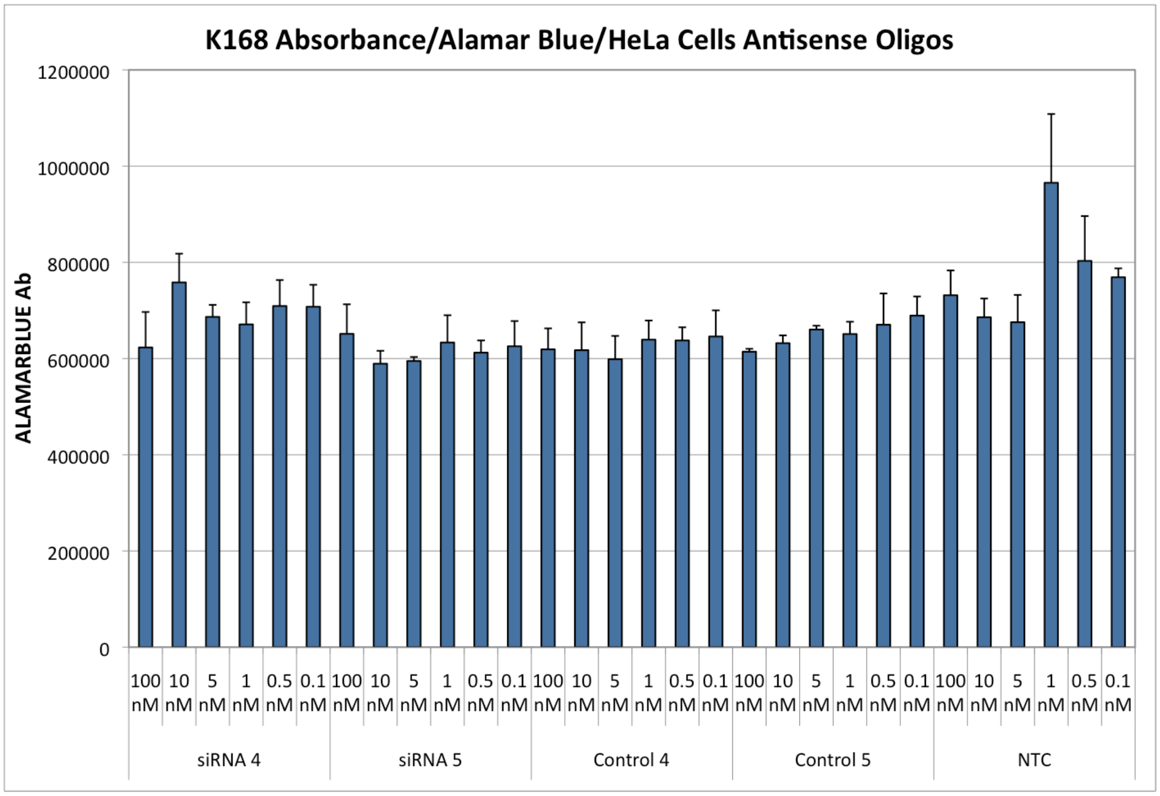

Figure S5. Cell viability for experiments shown in Figure 6 determined using a Resazurin assay.

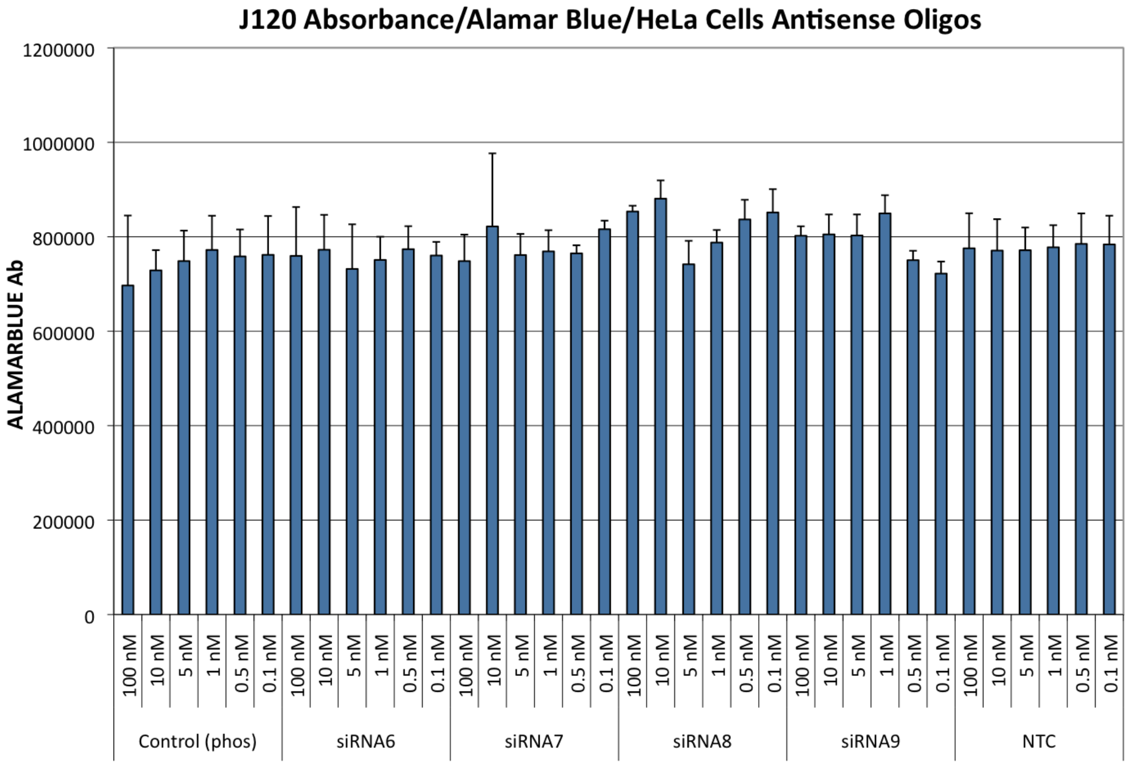

Figure S6. Cell viability for experiments shown in Figure 7 determined using a Resazurin assay.

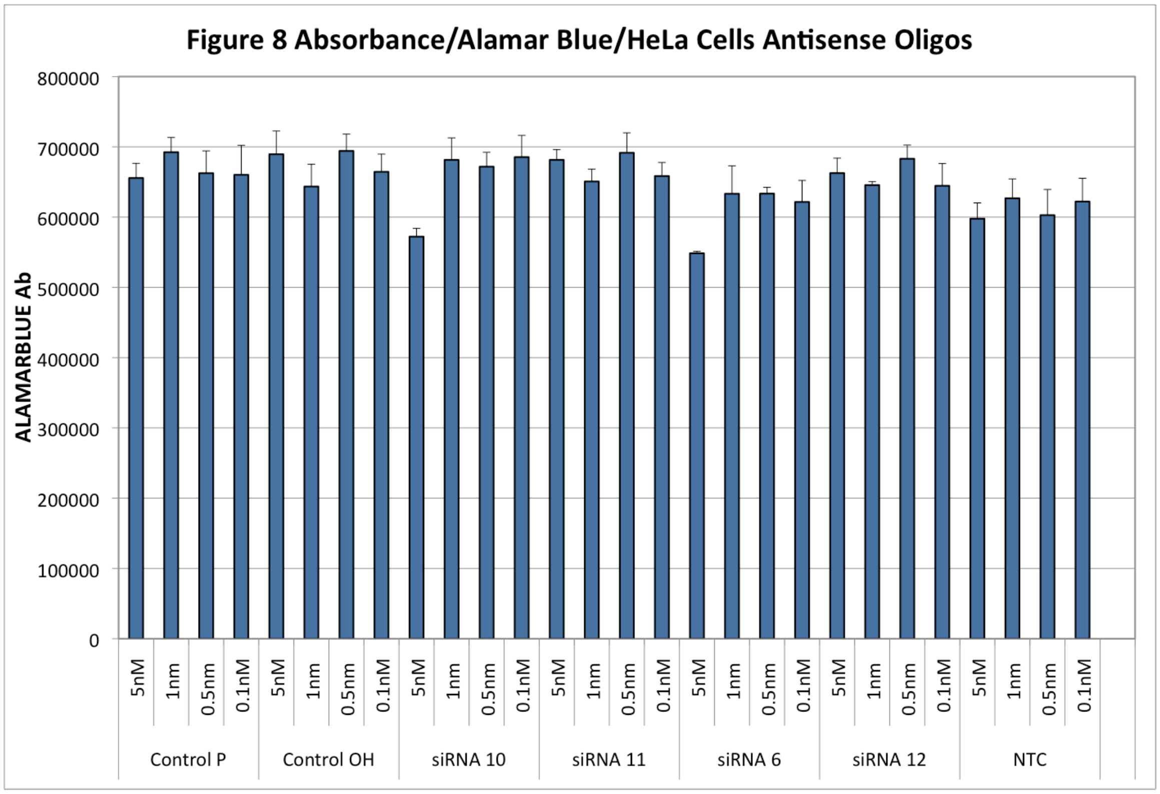

Figure S7. Cell viability for experiments shown in Figure 8 determined using a Resazurin assay.

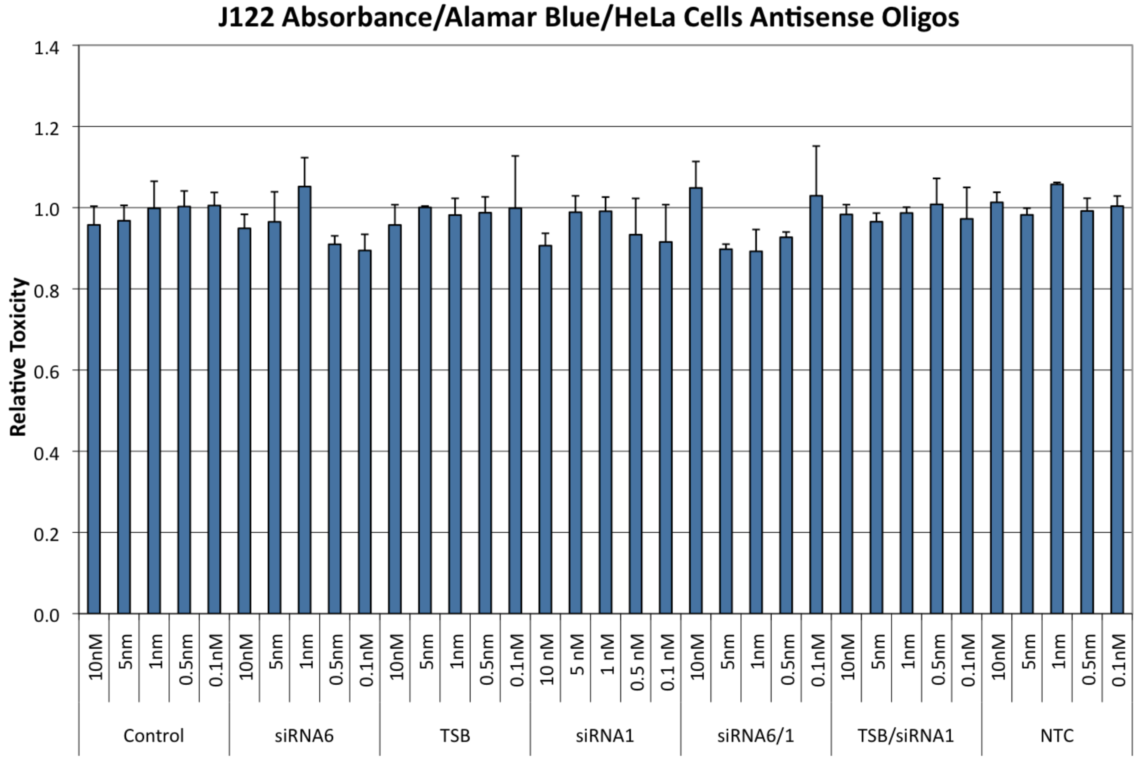

Figure S8. Cell viability for experiments shown in Figure 9 determined using an a Resazurin assay. The data are normalized from different plates.

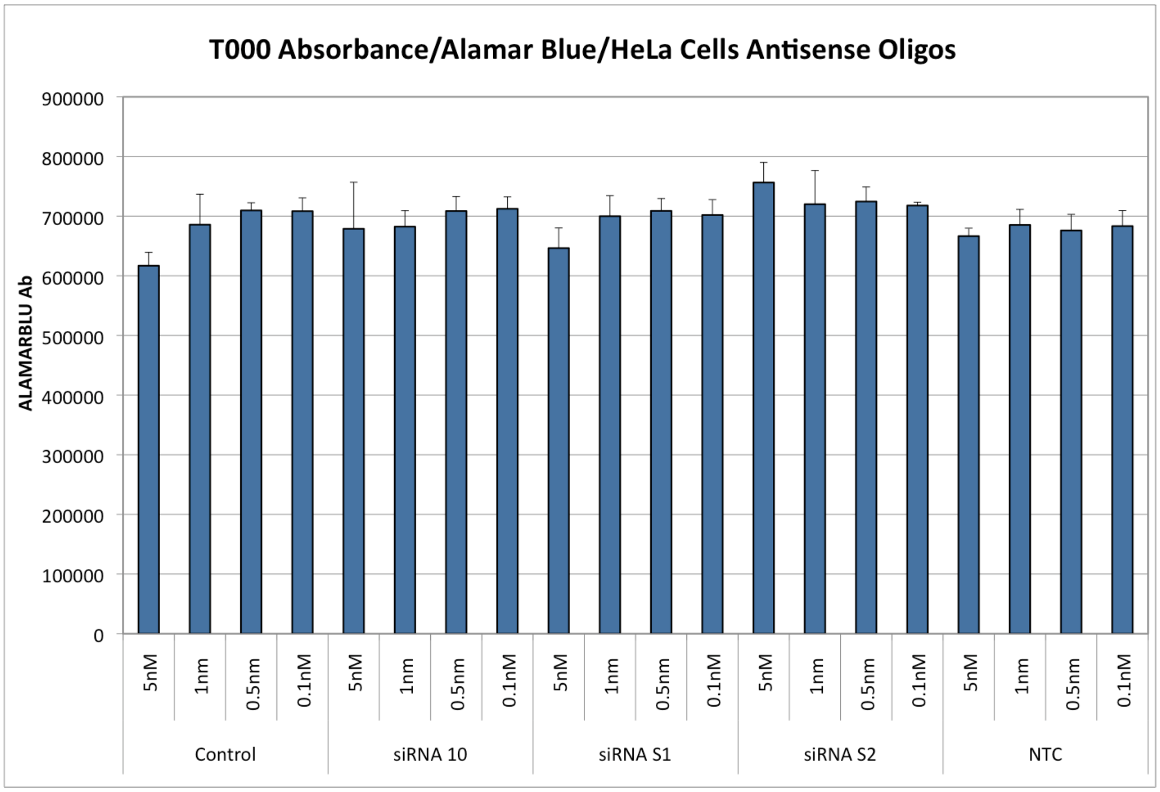

Figure S9. Cell viability for experiments shown in Figure S10A below determined using a Resazurin assay.

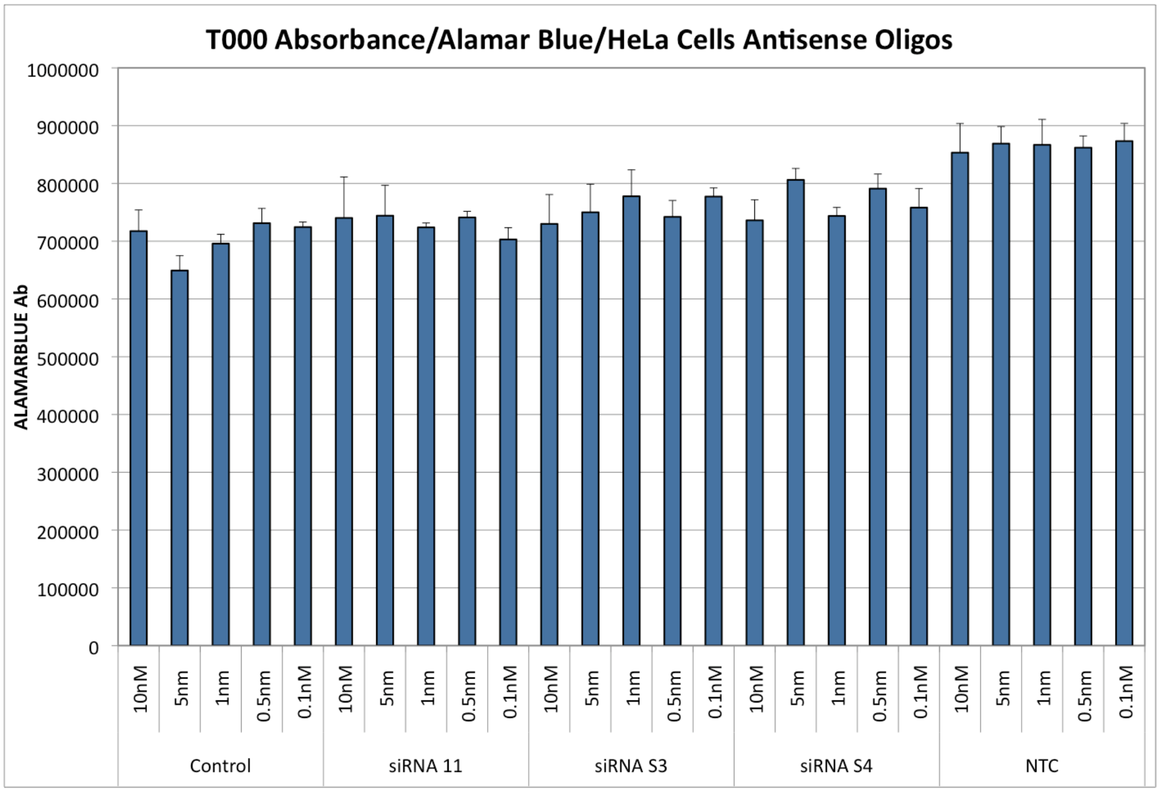

Figure S10. Cell viability for experiments shown in Figure S10B below determined using a Resazurin assay.

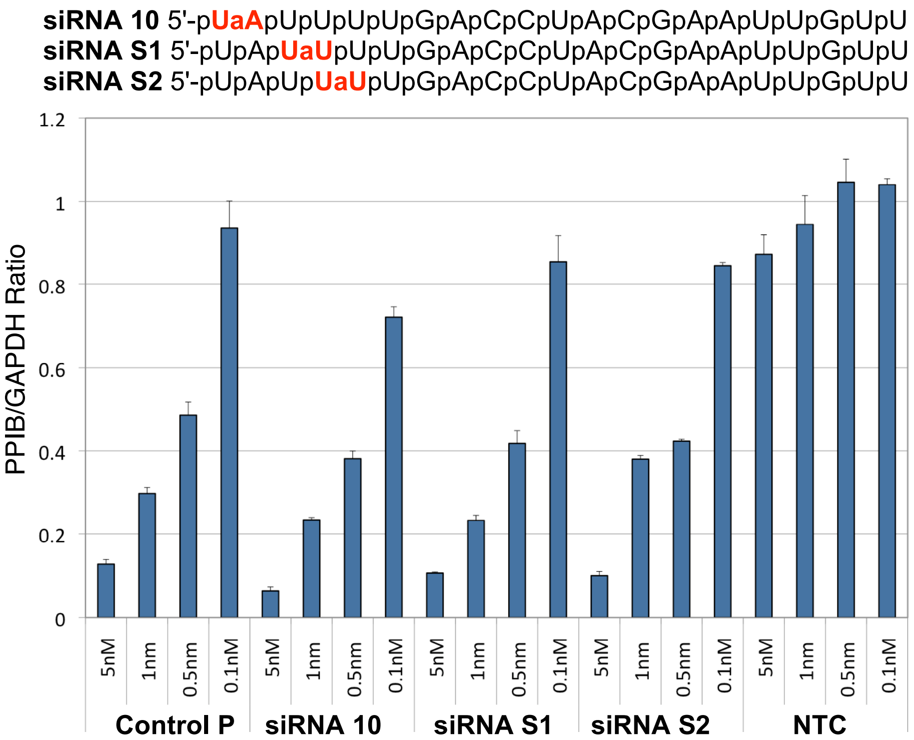

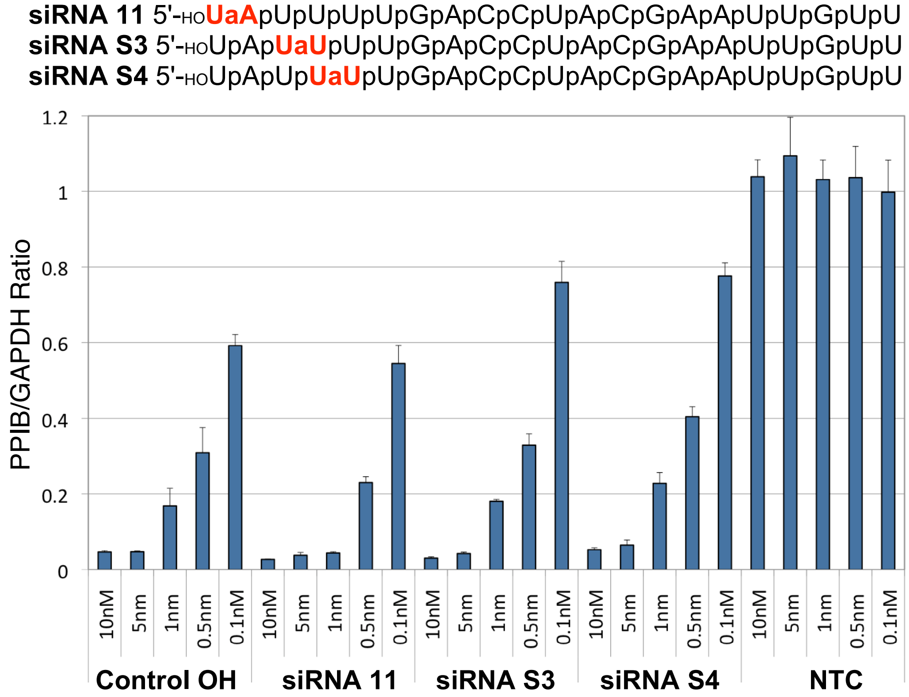

Figure S11. Comparison of siRNAs (PPIBHFS308) having individual 5′-end modifications in the passenger strand; upper panel - 5′-phosphorylated and lower panel 5′-OH. The results are expressed as ratios of target RNA (PPIB) and housekeeping RNA (GABDH) and are averages of the three replicates; the standard deviations are shown in the length of the error bars. **Control P** and **Control OH** are unmodified siRNAs with 5´-phosphate or with 5´-OH, respectively. **NTC** is a negative control of non-target siRNA.

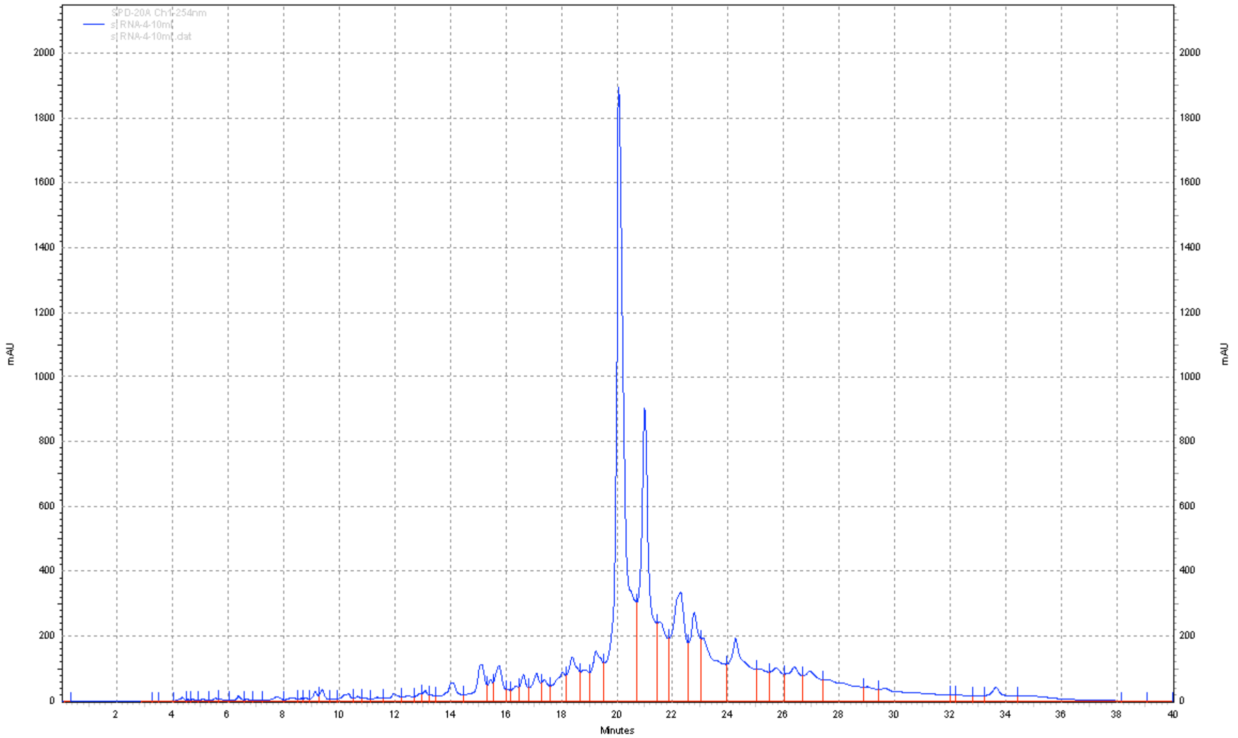

Figure S12. RP HPLC trace (XBridge™ C18 column, 4.6 × 150 mm) of crude **siRNA 1**; 92 nmols (yield 9 %) were isolated from the major peak; ESI MS calculated 6629.2; found 6628.6. **siRNA 1** was made using the 2´-OAc protected r($U_{\mathbf{AM1}}$U) and r($U_{\mathbf{AM1}}$A) phosphoramidites as described by us previously.[4]

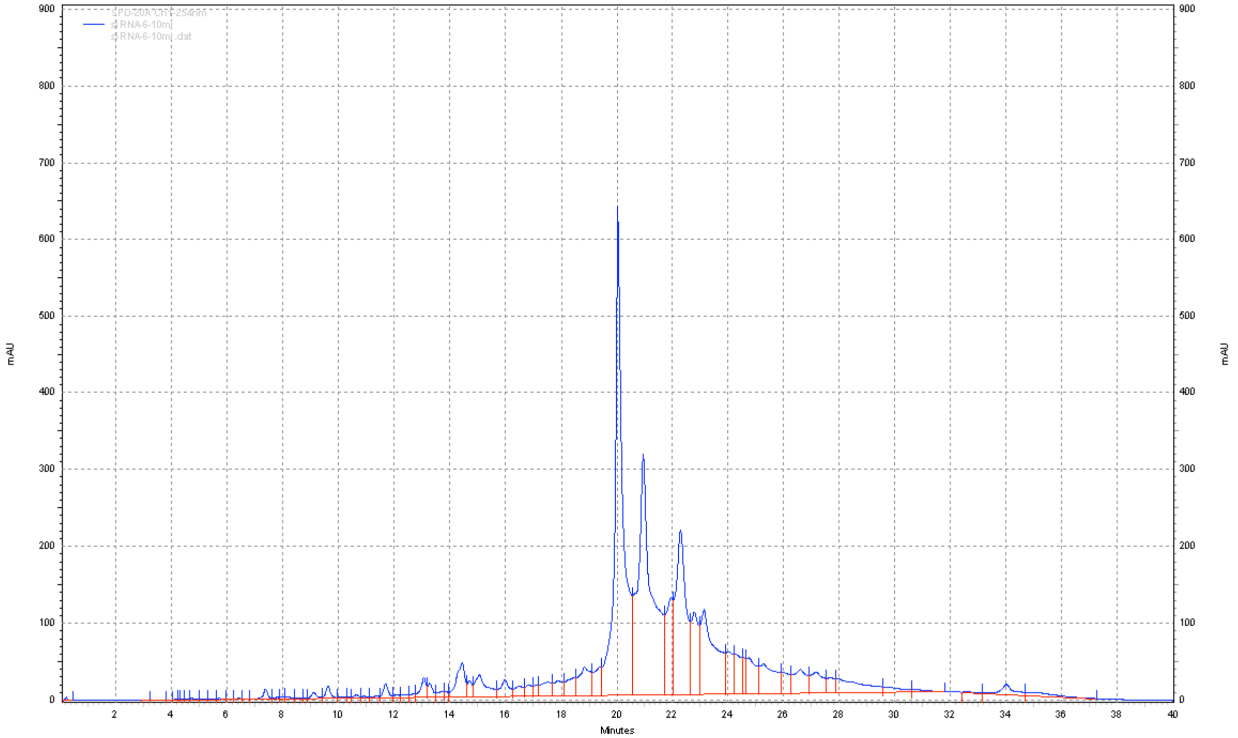

[4] Selvam, C., Thomas, S., Abbott, J., Kennedy, S.D. and Rozners, E. (2011) Amides as Excellent Mimics of Phosphate Linkages in RNA. *Angew. Chem., Int. Ed.*, **50**, 2068-2070.

Figure S13. RP HPLC trace (XBridge™ C18 column, 4.6 × 150 mm) of crude **siRNA 2**; 80 nmols (yield 8 %) were isolated from the major peak; ESI MS calculated 6707.1; found 6706.9. **siRNA 2** was made using the 2´-OAc protected r(U_{AM1}A) phosphoramidite as described by us previously.[4.]

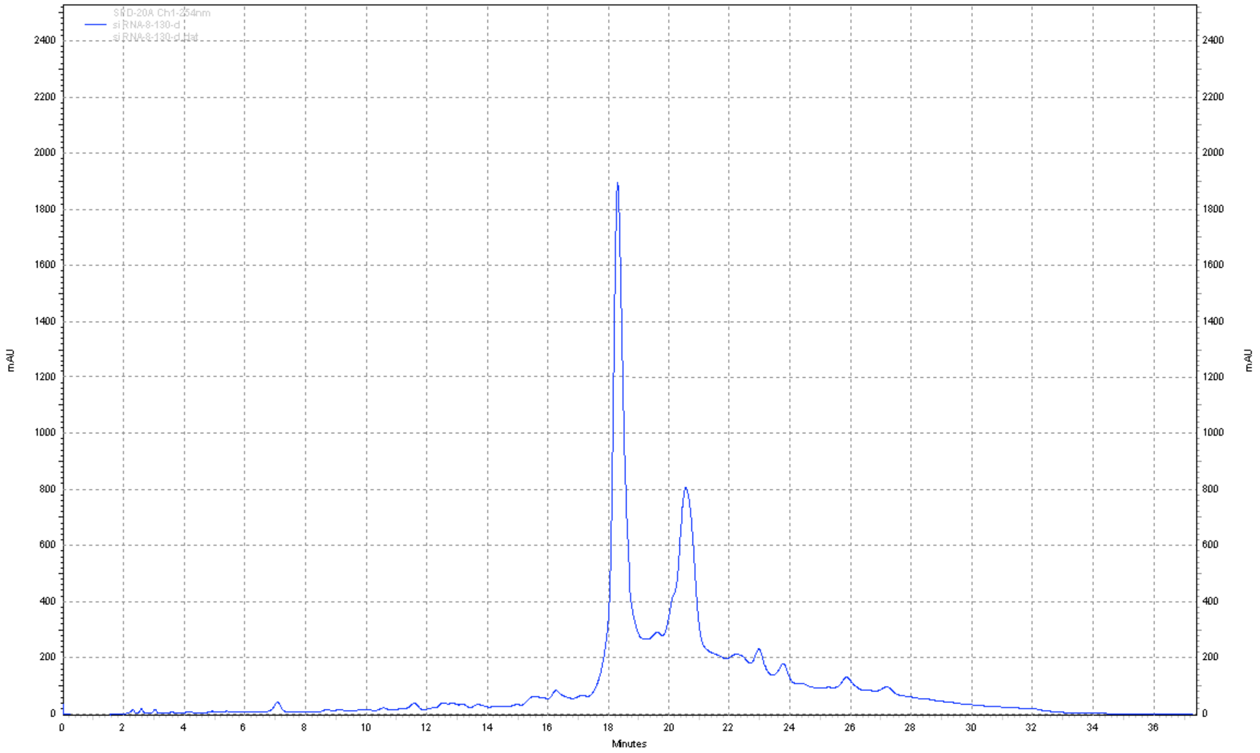

Figure S14. RP HPLC trace (XBridge™ C18 column, 4.6 × 150 mm) of crude **siRNA 3**; 65 nmols (yield 7 %) were isolated from the major peak; ESI MS calculated 6627.1; found 6626.8. **siRNA 3** was made using the 2´-OAc protected r(U_{AM1}A) phosphoramidite as described by us previously.[4]

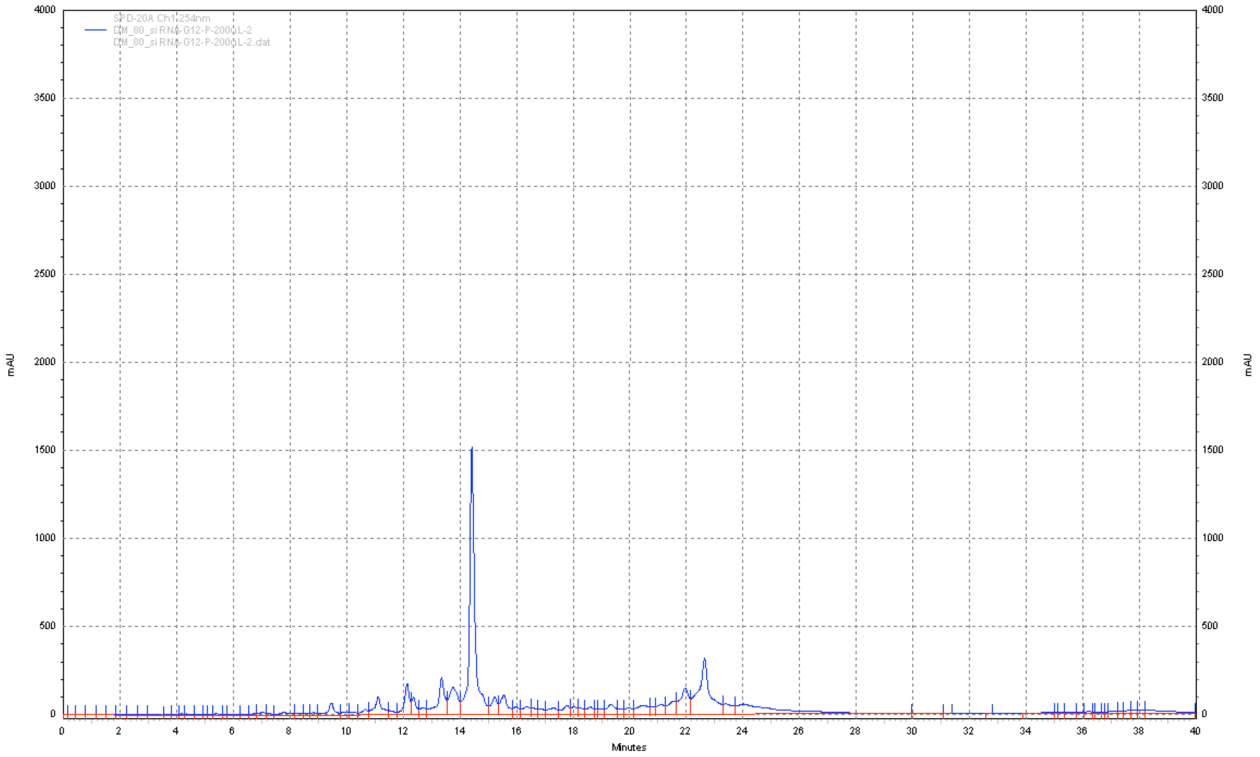

Figure S15. RP HPLC trace (XBridge™ C18 column, 10 × 150 mm) of crude **siRNA 4**; 8 nmols (yield 2 %) were isolated from the major peak; ESI MS calculated 6716.0; found 6718.3.

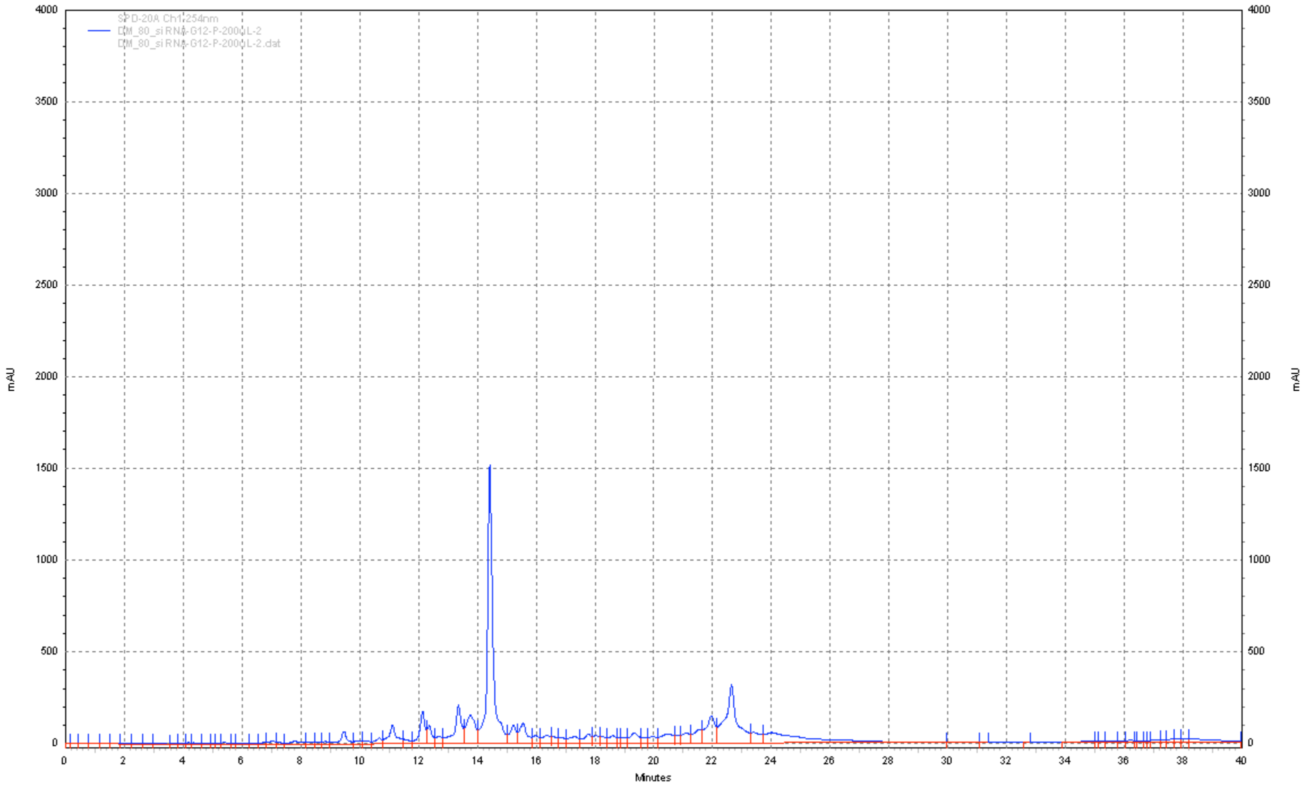

Figure S16. RP HPLC trace (XBridge™ C18 column, 10 × 150 mm) of crude **siRNA 5**; 6 nmols (yield 1 %) were isolated from the major peak; ESI MS calculated 6613.9; found 6616.6.

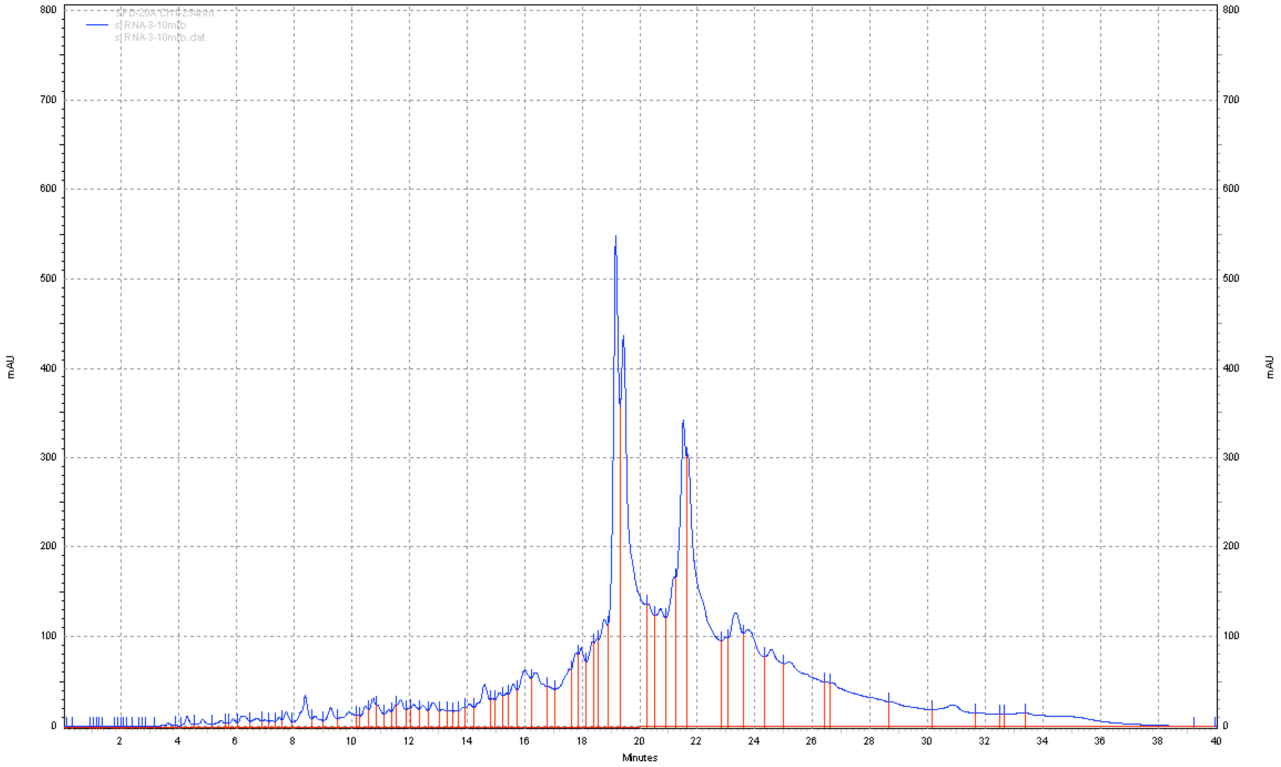

Figure S17. RP HPLC trace (XBridge™ C18 column, 4.6 × 150 mm) of crude **siRNA 6**; 51 nmols (yield 5 %) were isolated from the major peak; ESI MS calculated 6482.2; found 6482.0. **siRNA 6** was made using the 2′-OAc protected r($U_{\mathbf{AM1}}$U) and r($U_{\mathbf{AM1}}$A) phosphoramidites as described by us previously.[4]

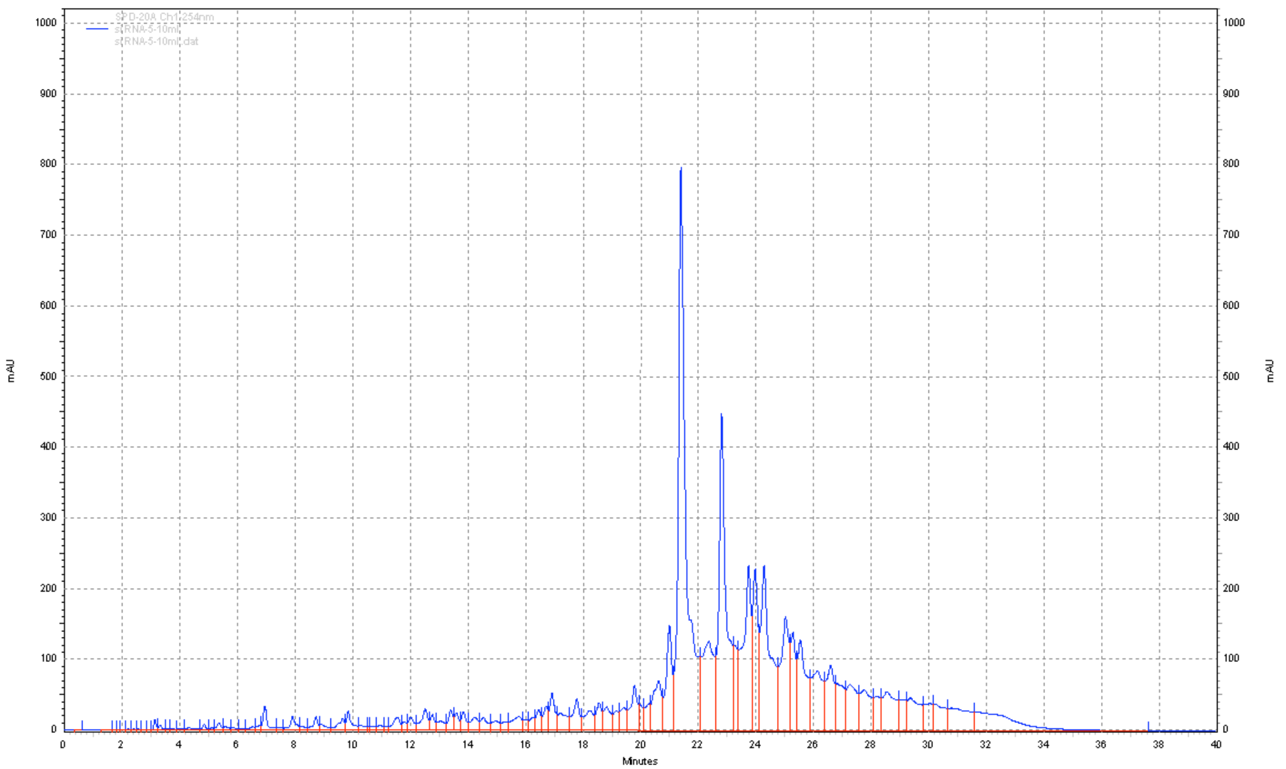

Figure S18. RP HPLC trace (XBridge™ C18 column, 4.6 × 150 mm) of crude **siRNA 7**; 75 nmols (yield 8 %) were isolated from the major peak; ESI MS calculated 6560.0; found 6559.4. **siRNA 7** was made using the 2′-OAc protected r($\mathrm{U_{AM1}}$U) and r($\mathrm{U_{AM1}}$A) phosphoramidites as described by us previously.[4]

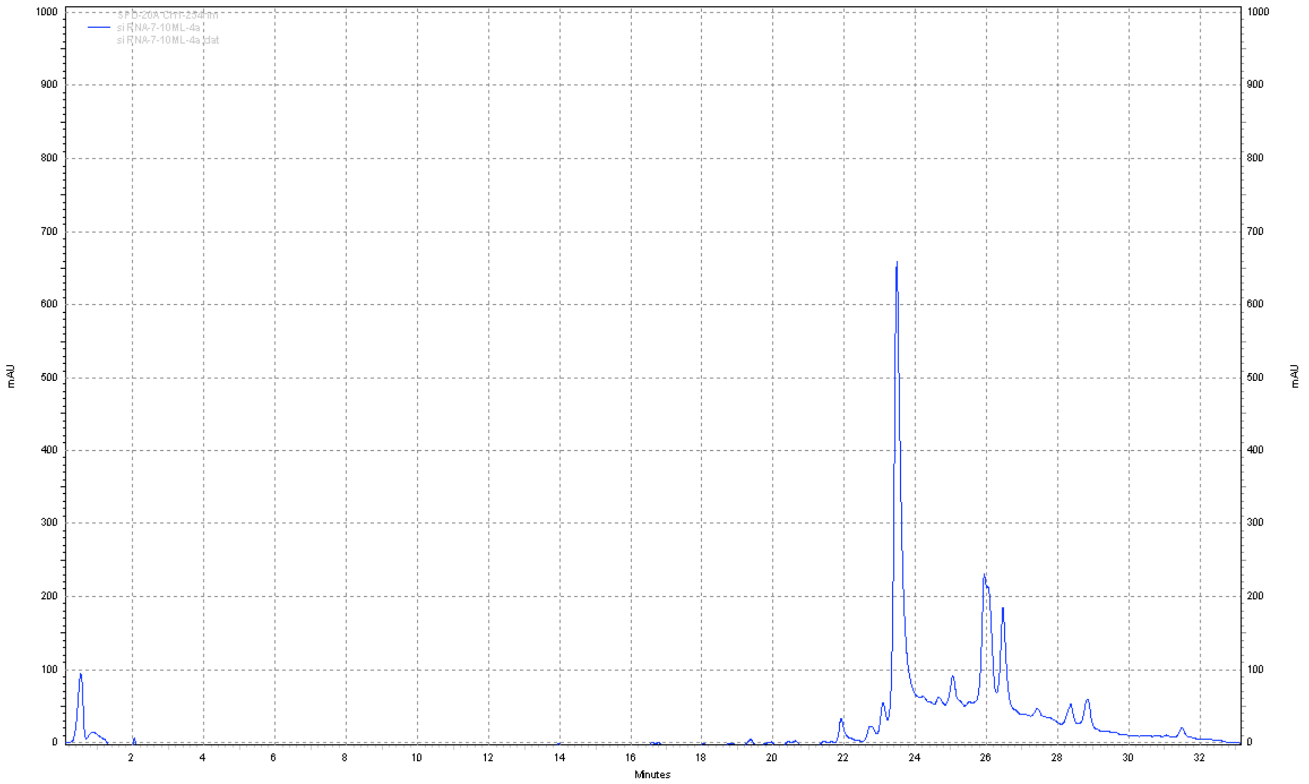

Figure S19. RP HPLC trace (XBridge™ C18 column, 4.6 × 150 mm) of crude **siRNA 8**; 113 nmols (yield 11 %) were isolated from the major peak; ESI MS calculated 6558.0; found 6557.5. **siRNA 8** was made using the 2′-OAc protected r($\mathrm{U_{AM1}}$U) phosphoramidite as described by us previously.[4]

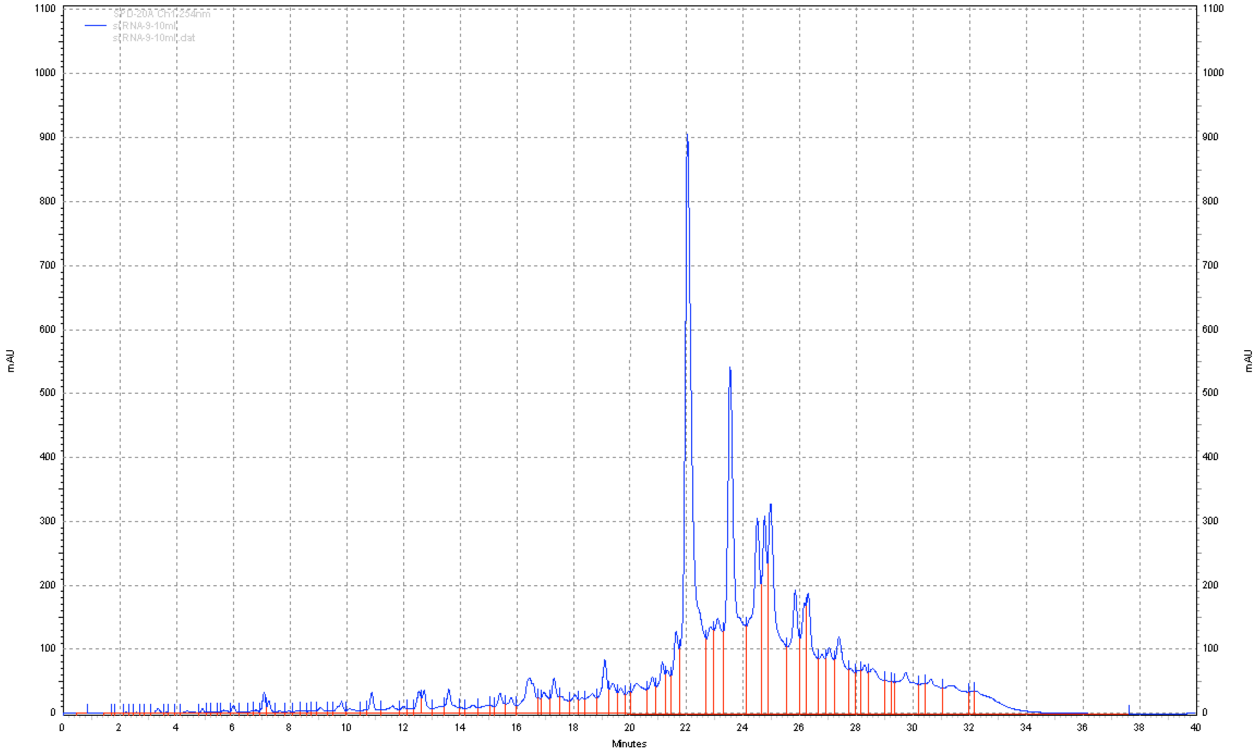

Figure S20. RP HPLC trace (XBridge™ C18 column, 4.6 × 150 mm) of crude **siRNA 9**; 63 nmols (yield 6 %) were isolated from the major peak; ESI MS calculated 6637.9; found 6638.2. **siRNA 9** was made using the 2´-OAc protected r(U_{AM1}U) phosphoramidite as described by us previously.[4]

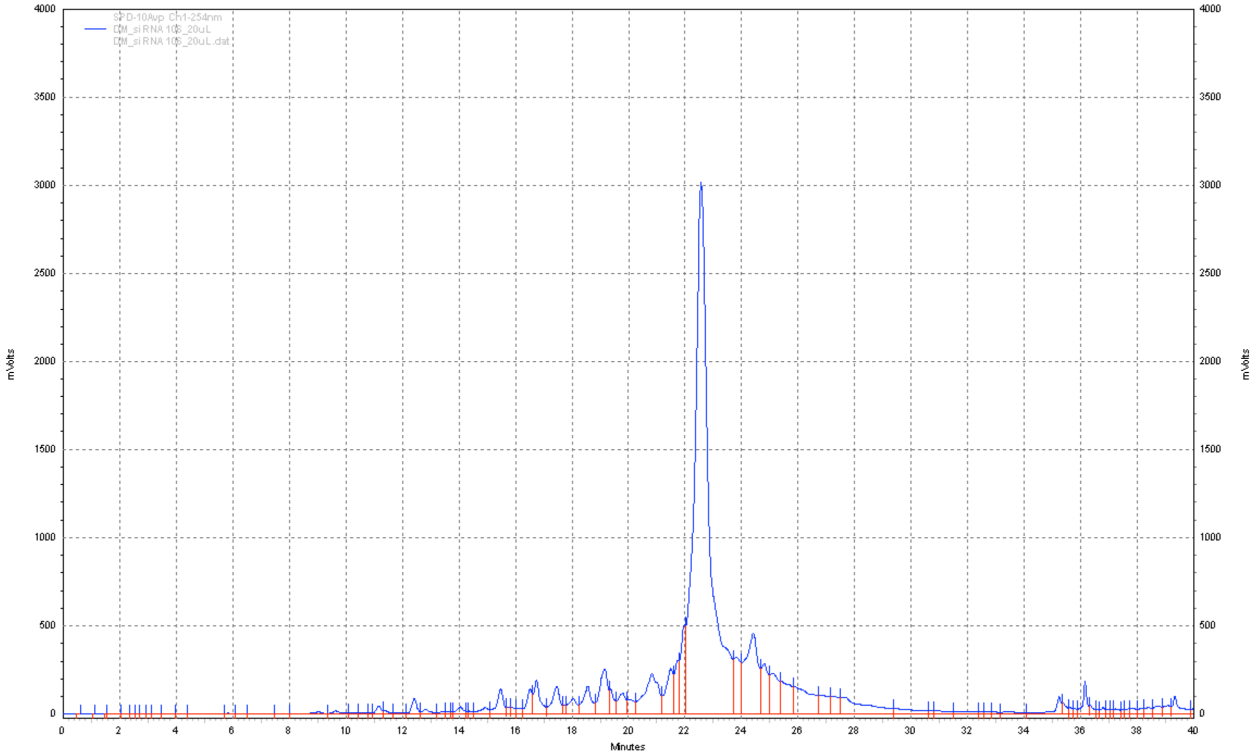

Figure S21. RP HPLC trace (XBridge™ C18 column, 4.6 × 150 mm) of crude **siRNA 10**; 160 nmols (yield 16 %) were isolated from the major peak; ESI MS calculated 6639.0; found 6640.3.

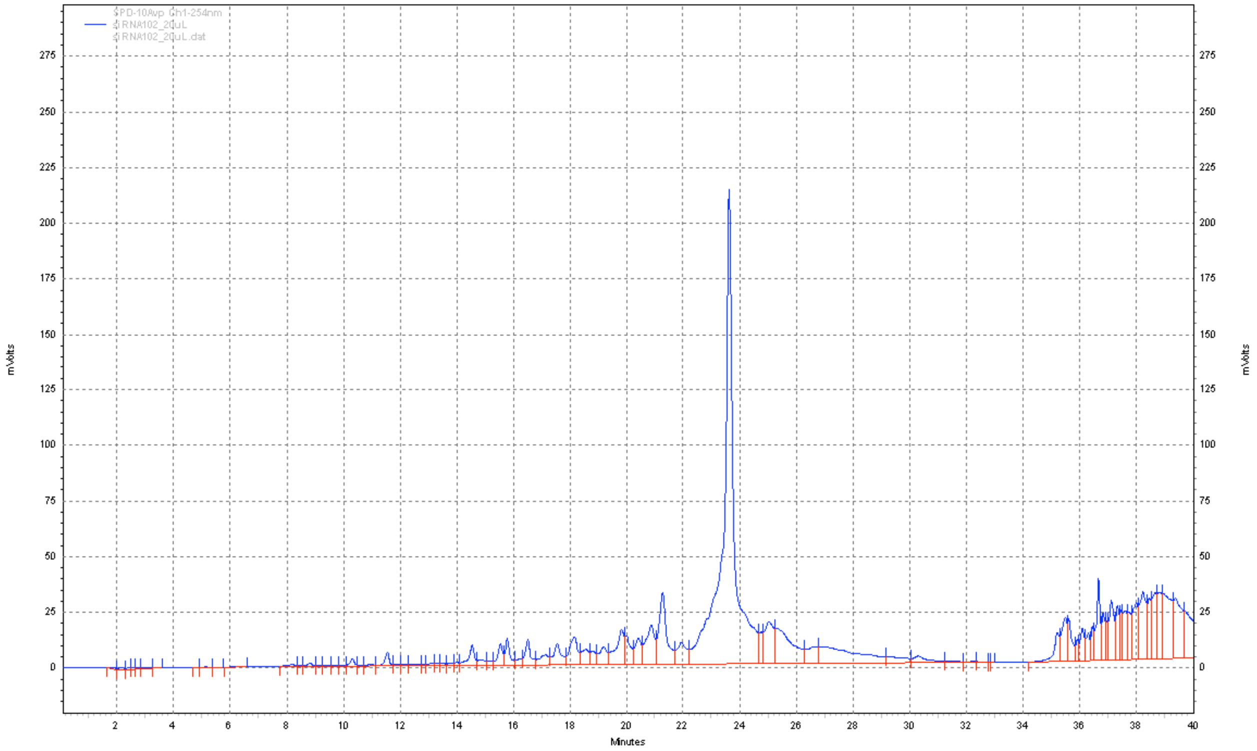

Figure S22. RP HPLC trace (XBridge™ C18 column, 4.6 × 150 mm) of crude **siRNA 11**; 44 nmols (yield 4 %) were isolated from the major peak; ESI MS calculated 6559.0; found 6563.1.

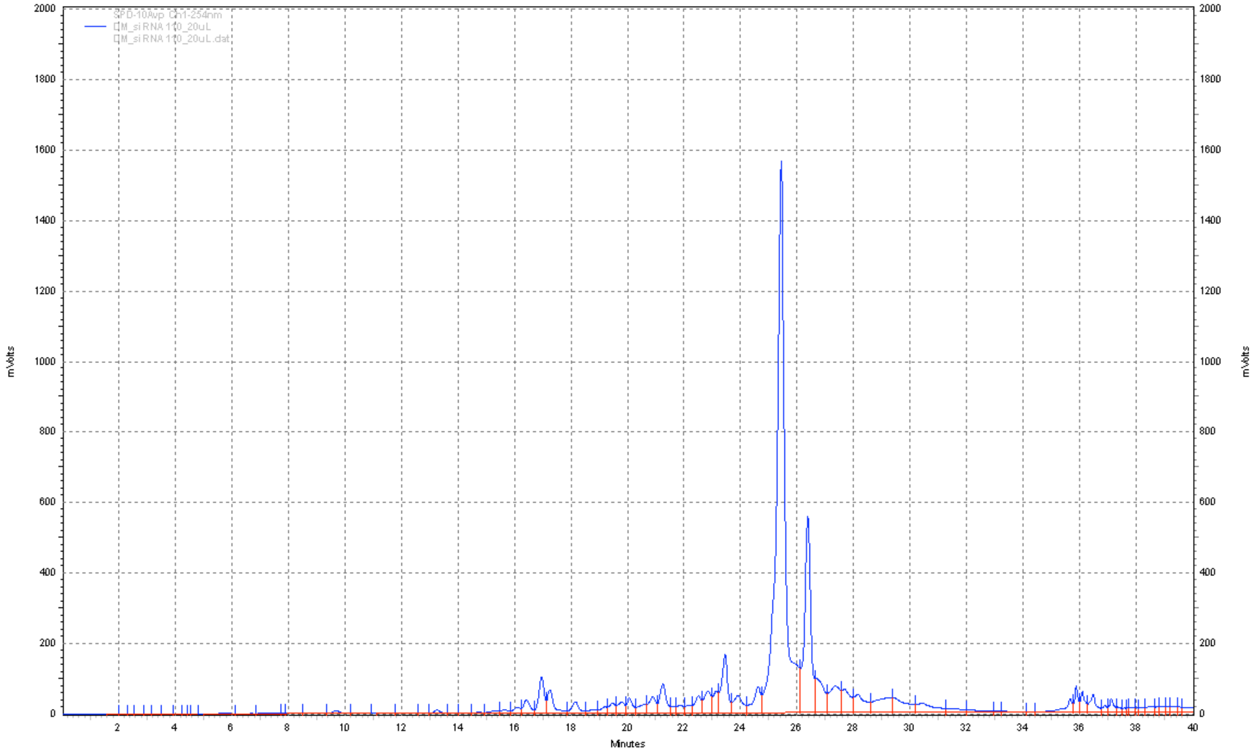

Figure S23. RP HPLC trace (XBridge™ C18 column, 4.6 × 150 mm) of crude **siRNA 12**; 139 nmols (yield 14 %) were isolated from the major peak; ESI MS calculated 6403.3; found 6406.2.

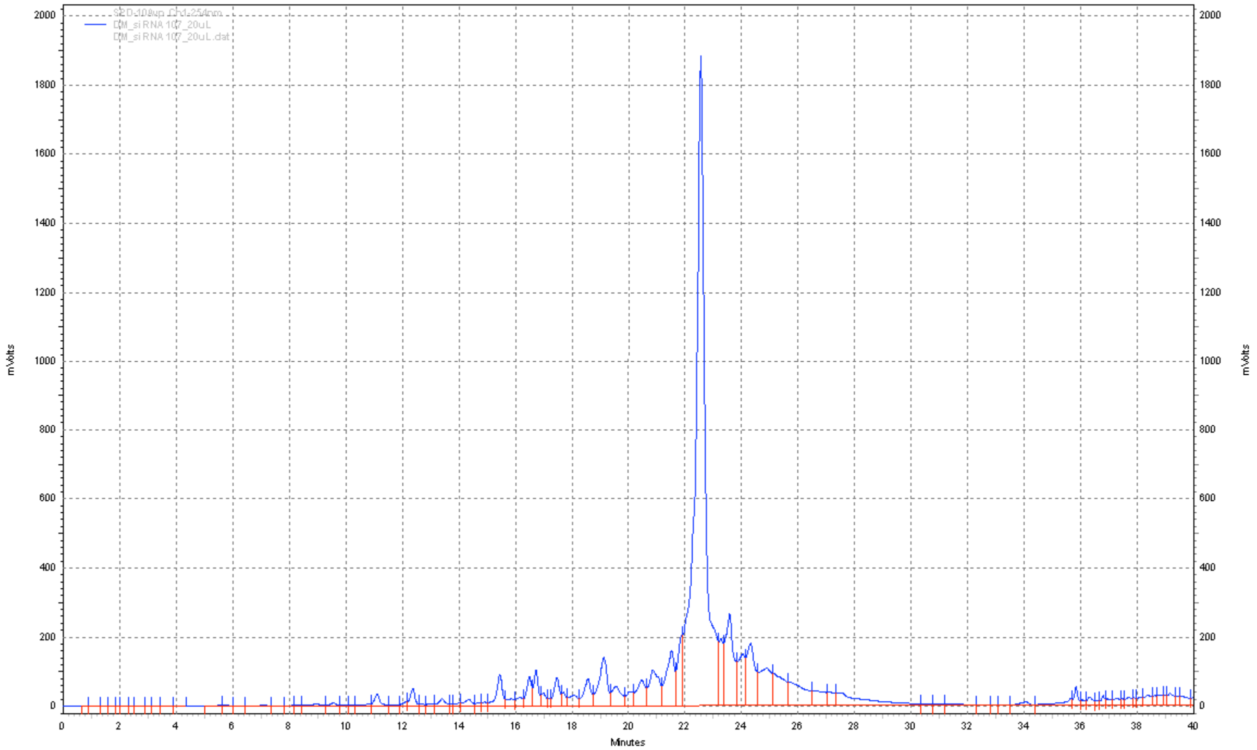

Figure S24. RP HPLC trace (XBridge™ C18 column, 4.6 × 150 mm) of crude **siRNA S1**; 101 nmols (yield 20 %) were isolated from the major peak; ESI MS calculated 6639.0; found 6640.3.

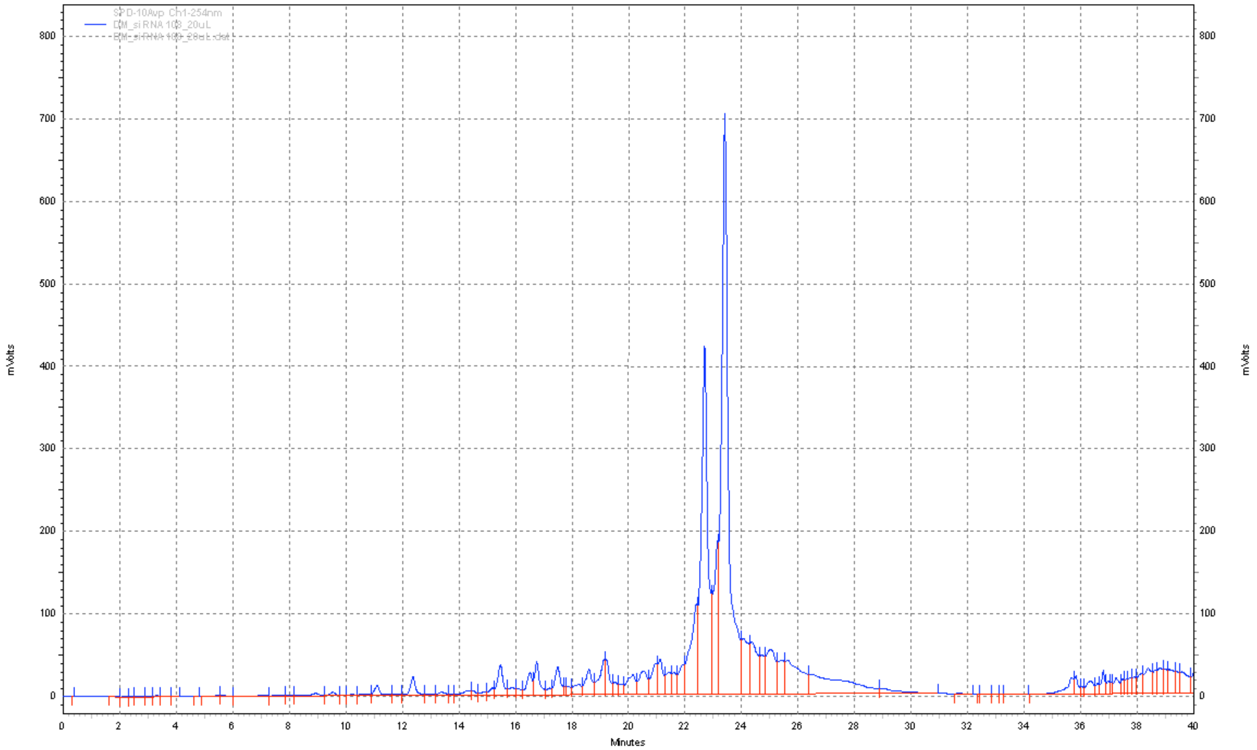

Figure S25. RP HPLC trace (XBridge™ C18 column, 4.6 × 150 mm) of crude **siRNA S2**; 44 nmols (yield 9 %) were isolated from the major peak; ESI MS calculated 6639.0; found 6640.3.

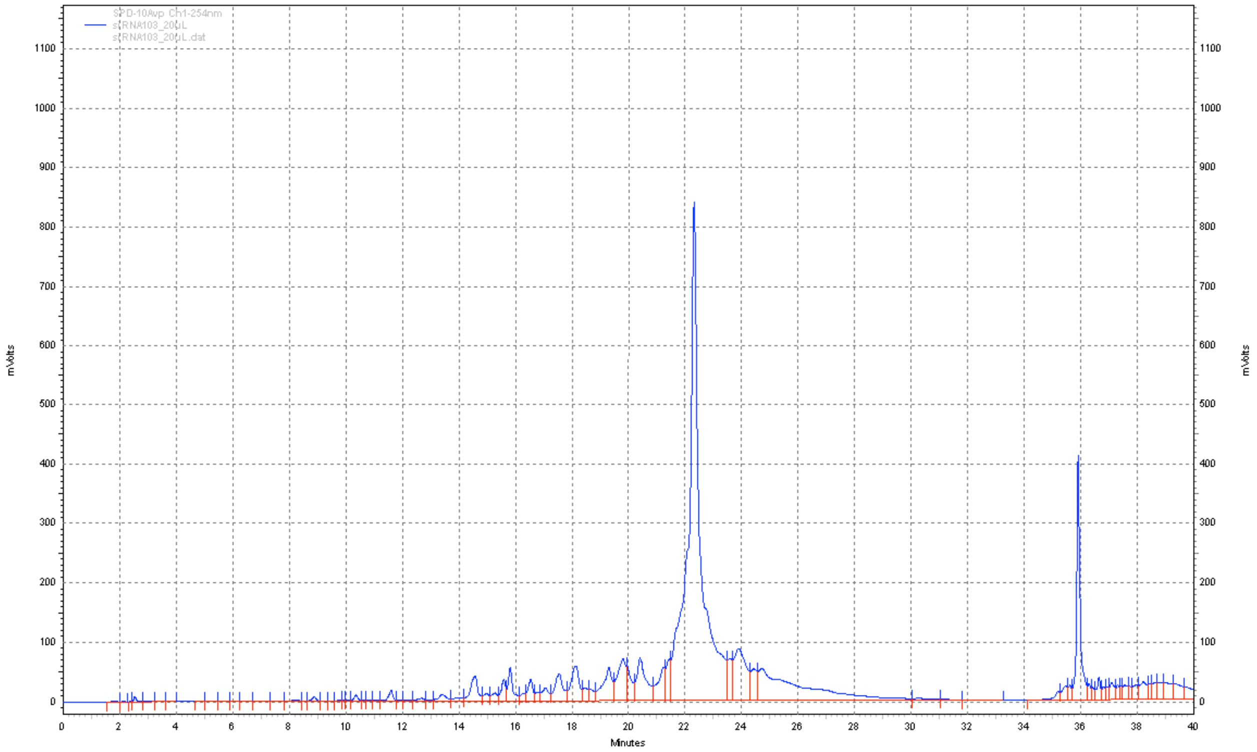

Figure S26. RP HPLC trace (XBridge™ C18 column, 4.6 × 150 mm) of crude **siRNA S3**; 90 nmols (yield 18 %) were isolated from the major peak; ESI MS calculated 6559.0; found 6559.3.

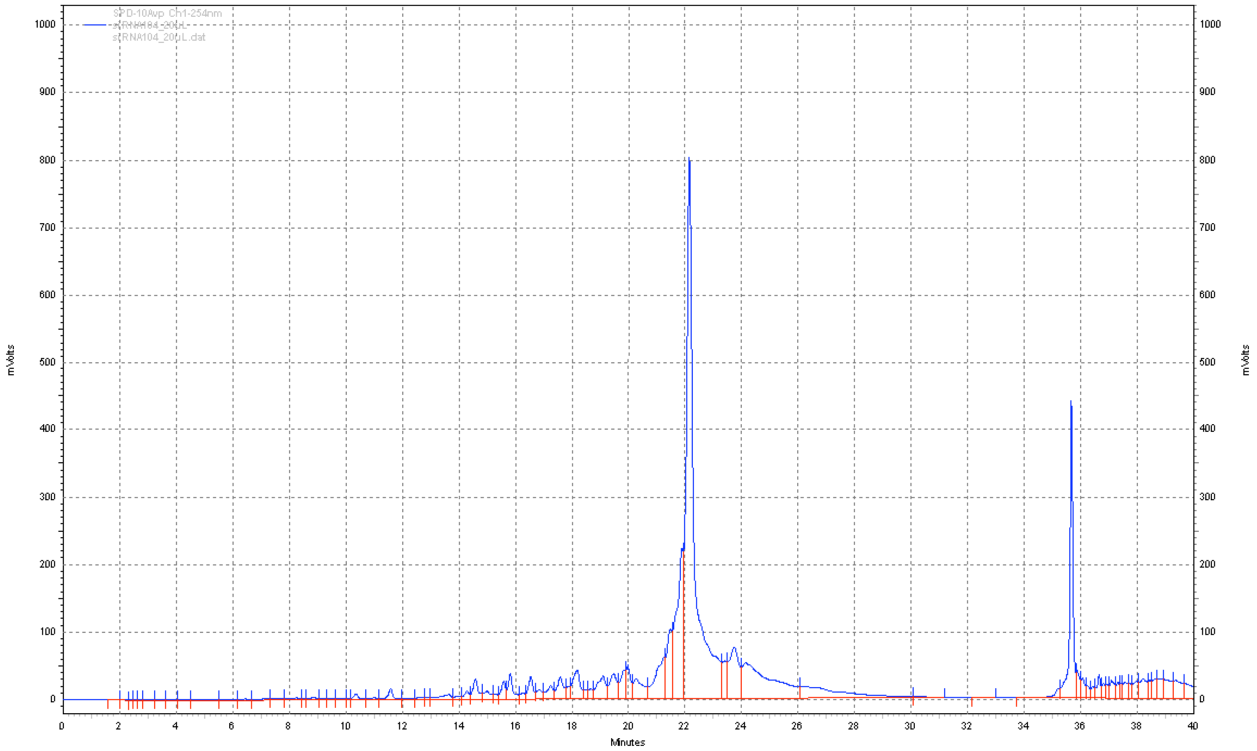

Figure S27. RP HPLC trace (XBridge™ C18 column, 4.6 × 150 mm) of crude **siRNA S4**; 100 nmols (yield 20 %) were isolated from the major peak; ESI MS calculated 6559.0; found 6563.1.

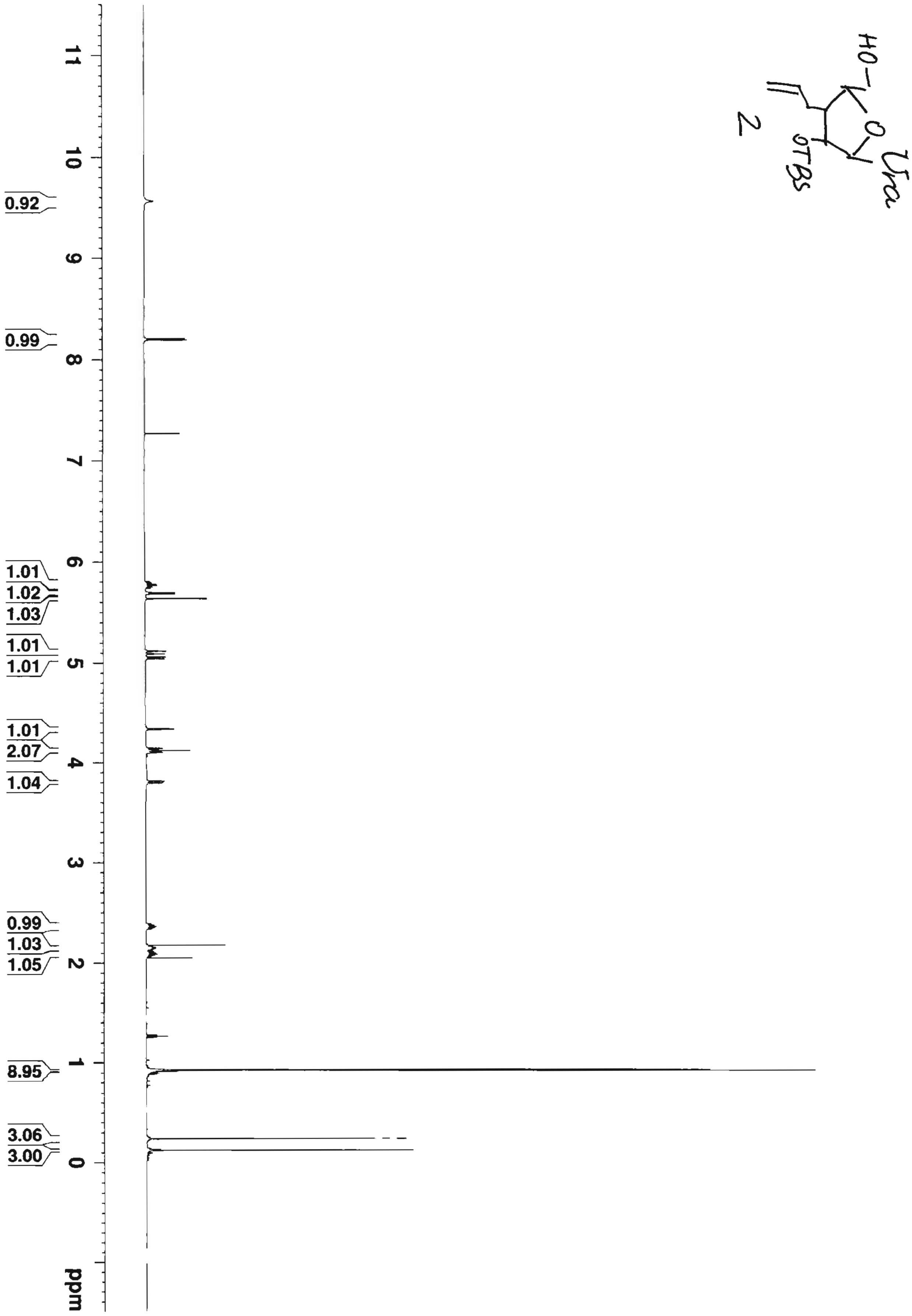

HO
OTBS
Ura
2
0.92
0.99
1.01
1.02
1.03
1.01
1.01
1.01
2.07
1.04
0.99
1.03
1.05
8.95
3.06
3.00
11
10
9
8
7
6
5
4
3
2
1
0
ppm

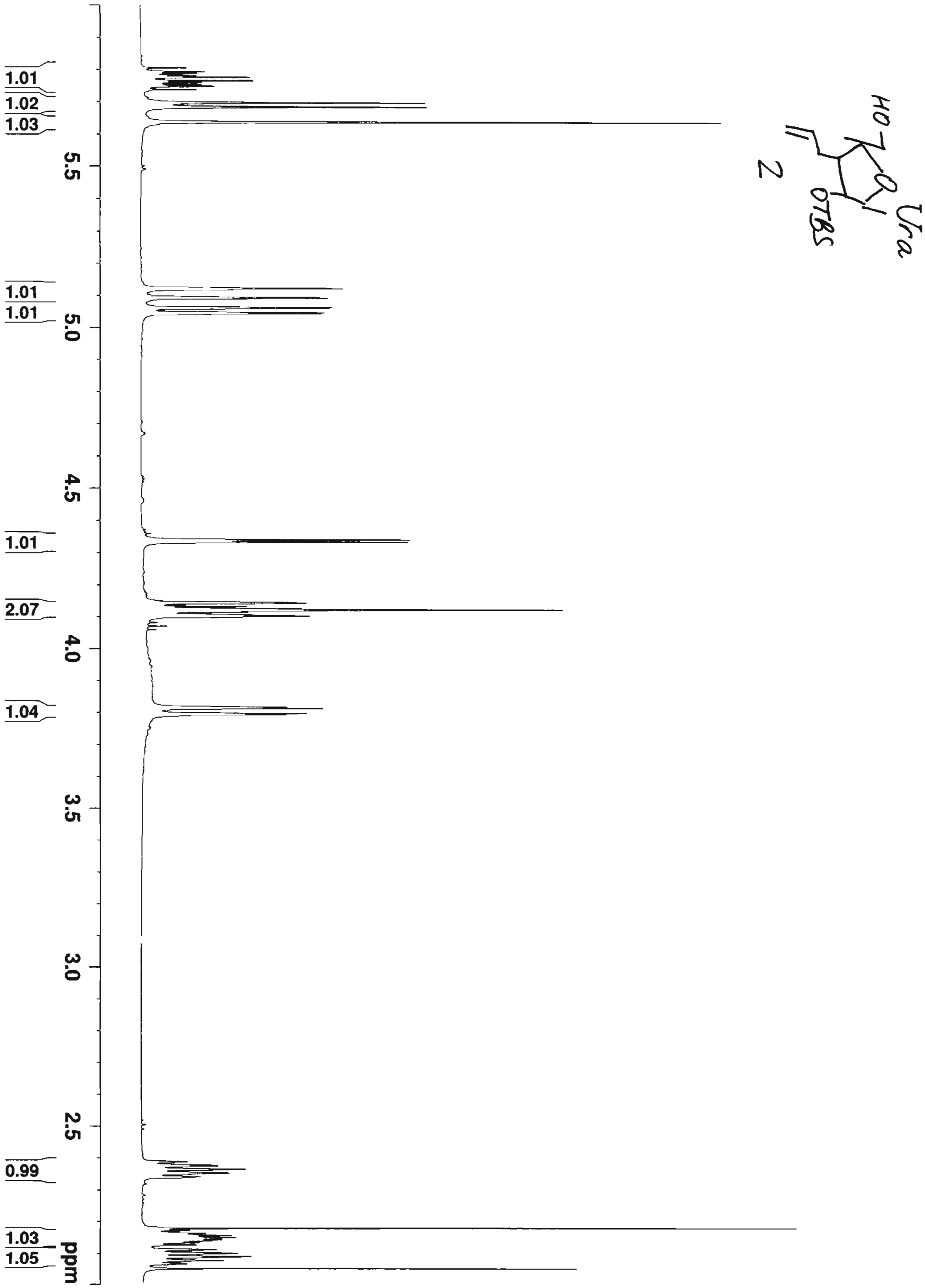
HO
O
Ura
OTBS
2
1.01
1.02
1.03
5.5
1.01
1.01
5.0
4.5
1.01
2.07
4.0
1.04
3.5
3.0
2.5
0.99
1.03
1.05
ppm

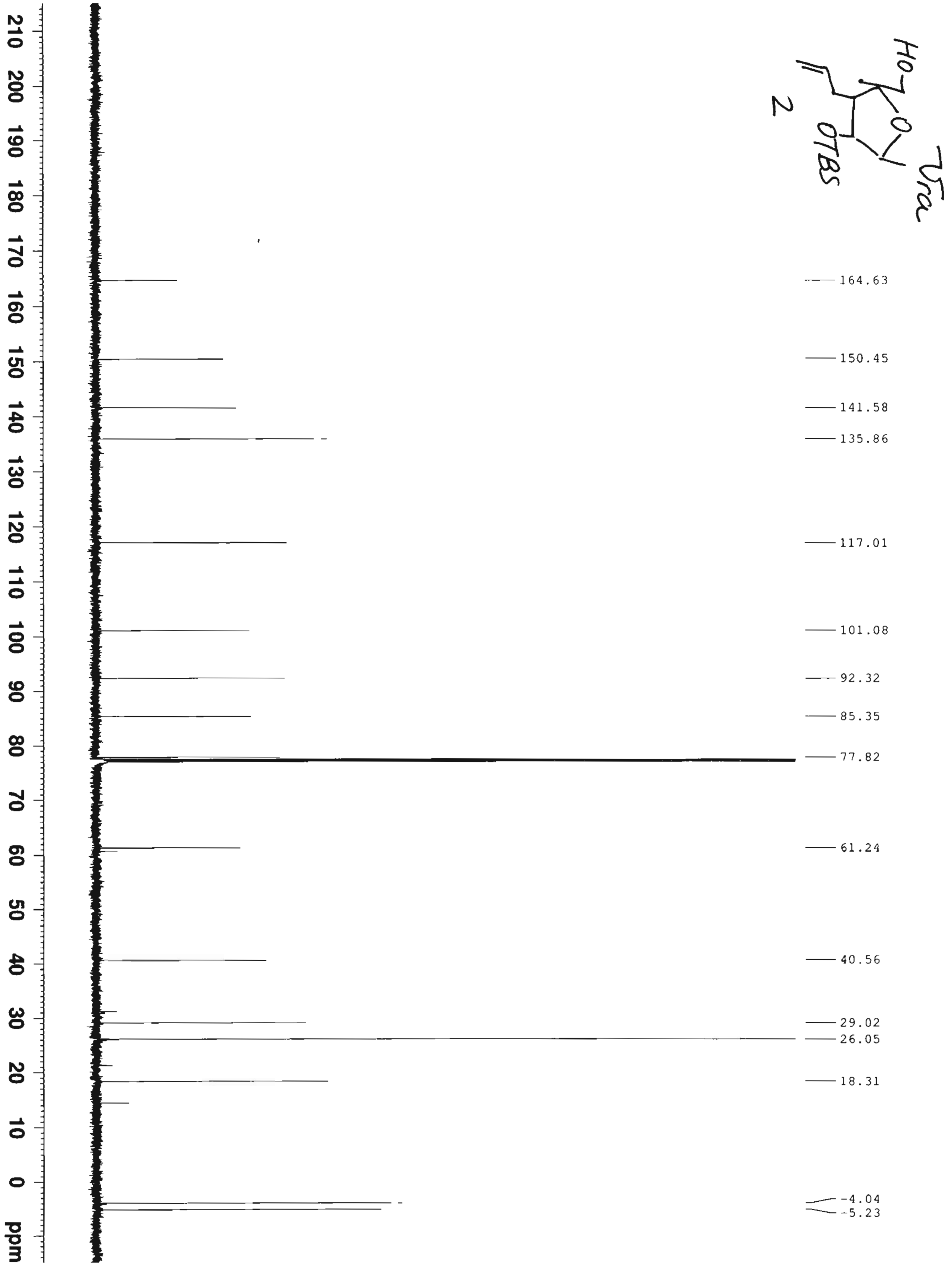

164.63
150.45
141.58
135.86
117.01
101.08
92.32
85.35
77.82
61.24
40.56
29.02
26.05
18.31
-4.04
-5.23
210 200 190 180 170 160 150 140 130 120 110 100 90 80 70 60 50 40 30 20 10 0 ppm

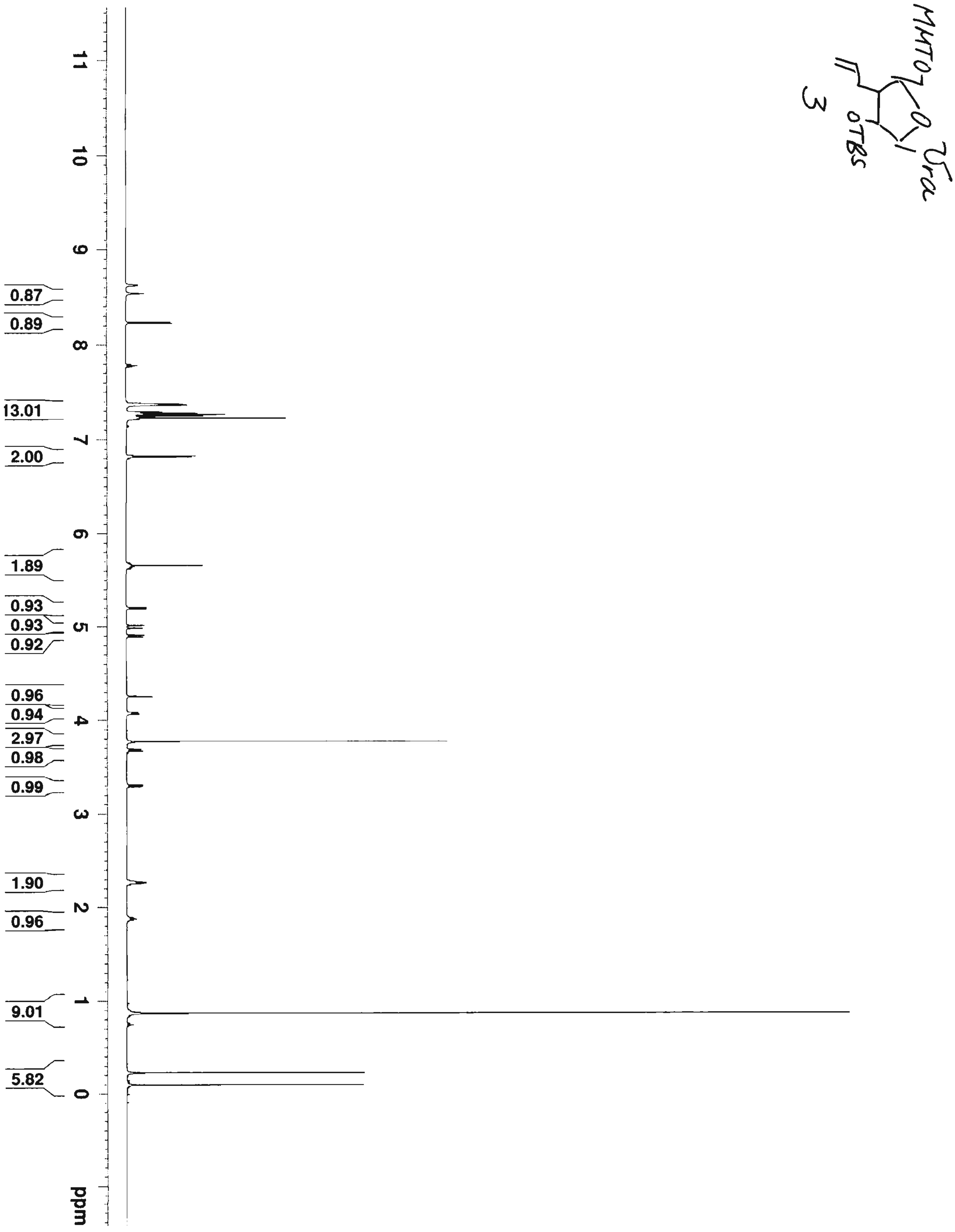
MMTO
Ura
OTBS
3
11
10
9
8
7
6
5
4
3
2
1
0
ppm
0.87
0.89
13.01
2.00
1.89
0.93
0.93
0.92
0.96
0.94
2.97
0.98
0.99
1.90
0.96
9.01
5.82

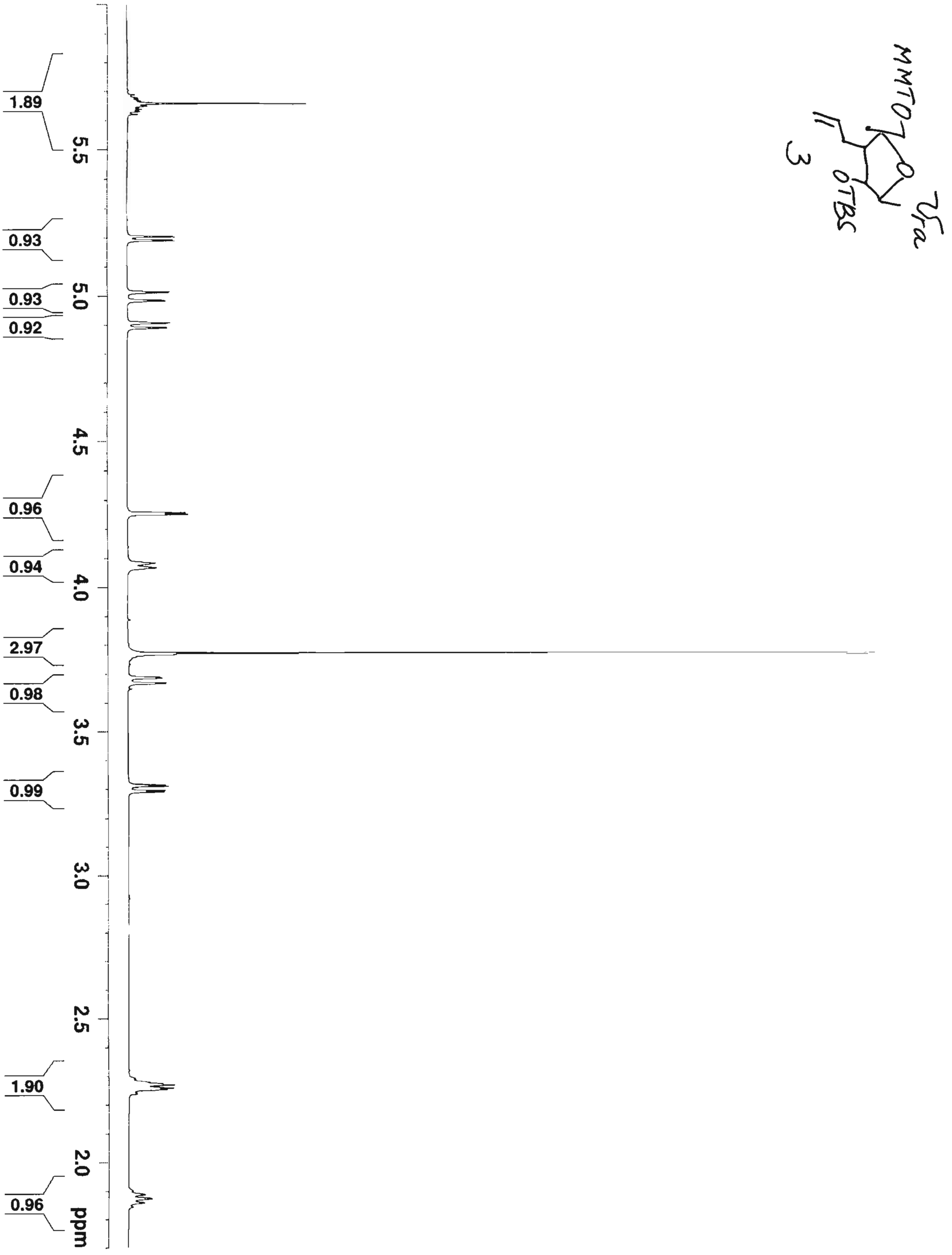

MMTO
Ura
OTBS
3
1.89
0.93
0.93
0.92
0.96
0.94
2.97
0.98
0.99
1.90
0.96
5.5
5.0
4.5
4.0
3.5
3.0
2.5
2.0
ppm

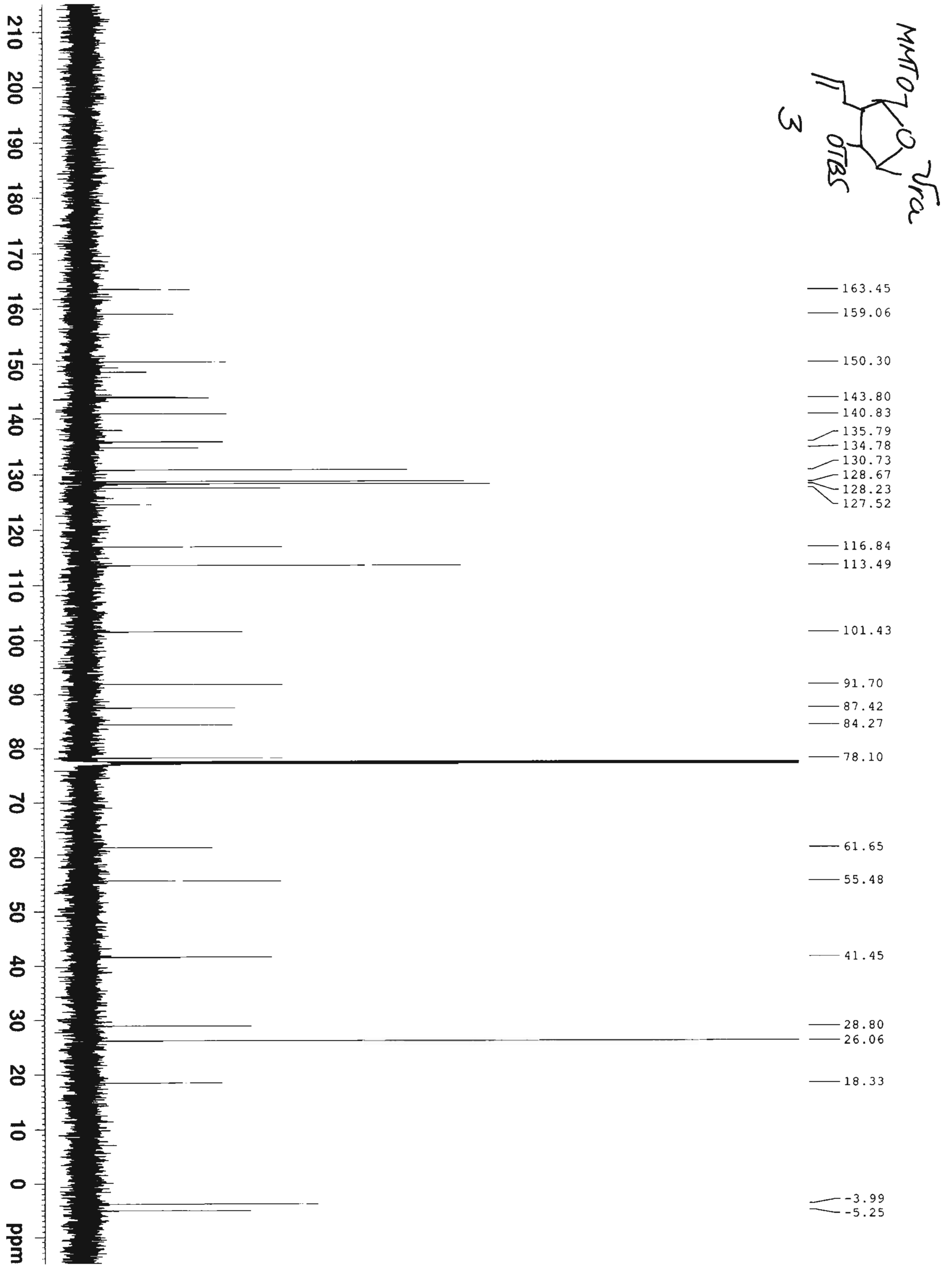
MMTrO
Ura
OTBS
3
163.45
159.06
150.30
143.80
140.83
135.79
134.78
130.73
128.67
128.23
127.52
116.84
113.49
101.43
91.70
87.42
84.27
78.10
61.65
55.48
41.45
28.80
26.06
18.33
-3.99
-5.25
210 200 190 180 170 160 150 140 130 120 110 100 90 80 70 60 50 40 30 20 10 0 ppm

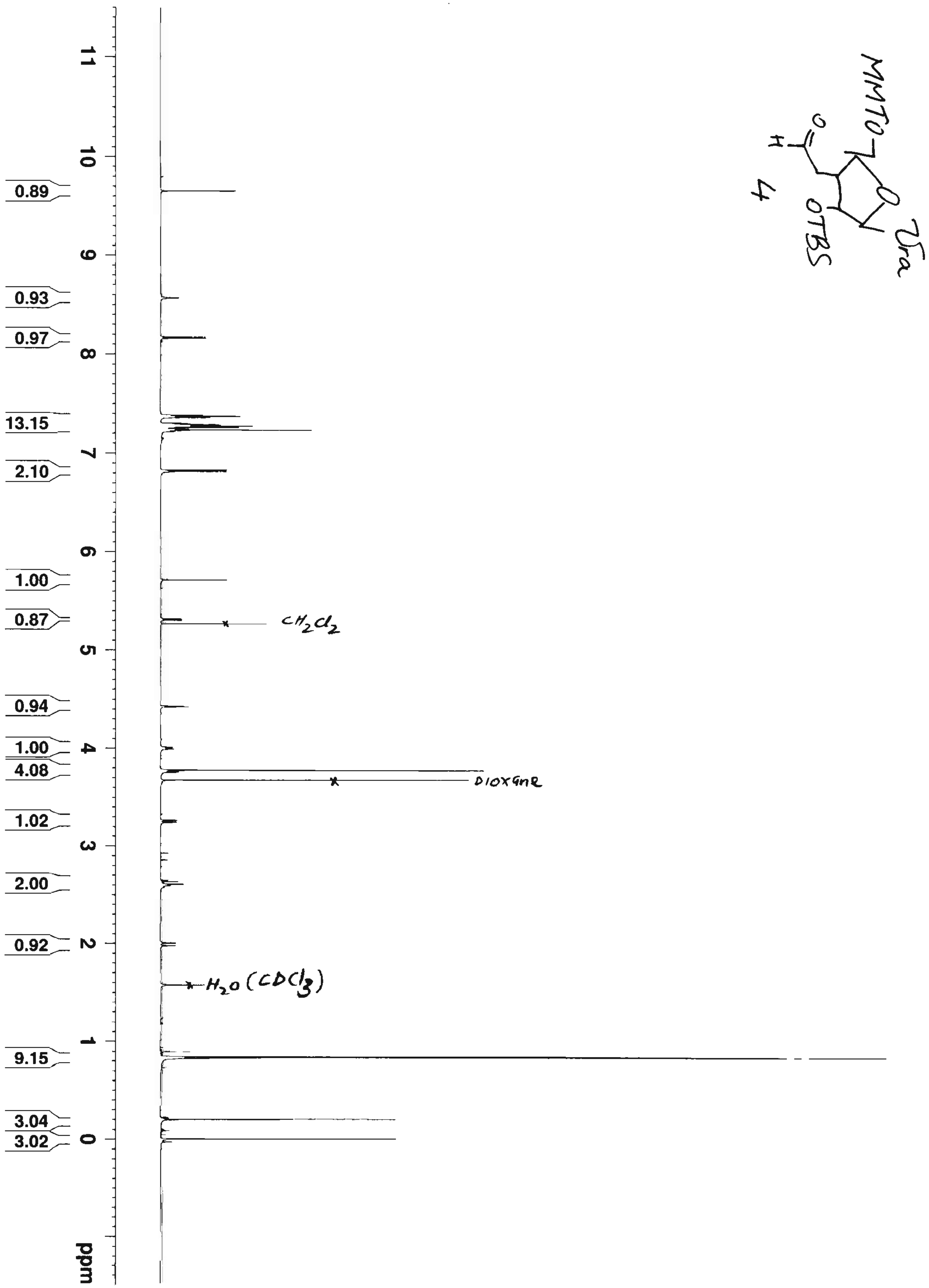

MMTO
Ura
OTBS
H
O
4
0.89
0.93
0.97
13.15
2.10
1.00
0.87
0.94
1.00
4.08
1.02
2.00
0.92
9.15
3.04
3.02
11
10
9
8
7
6
5
4
3
2
1
0
ppm
CH2Cl2
DIOXANE
H2O (CDCl3)

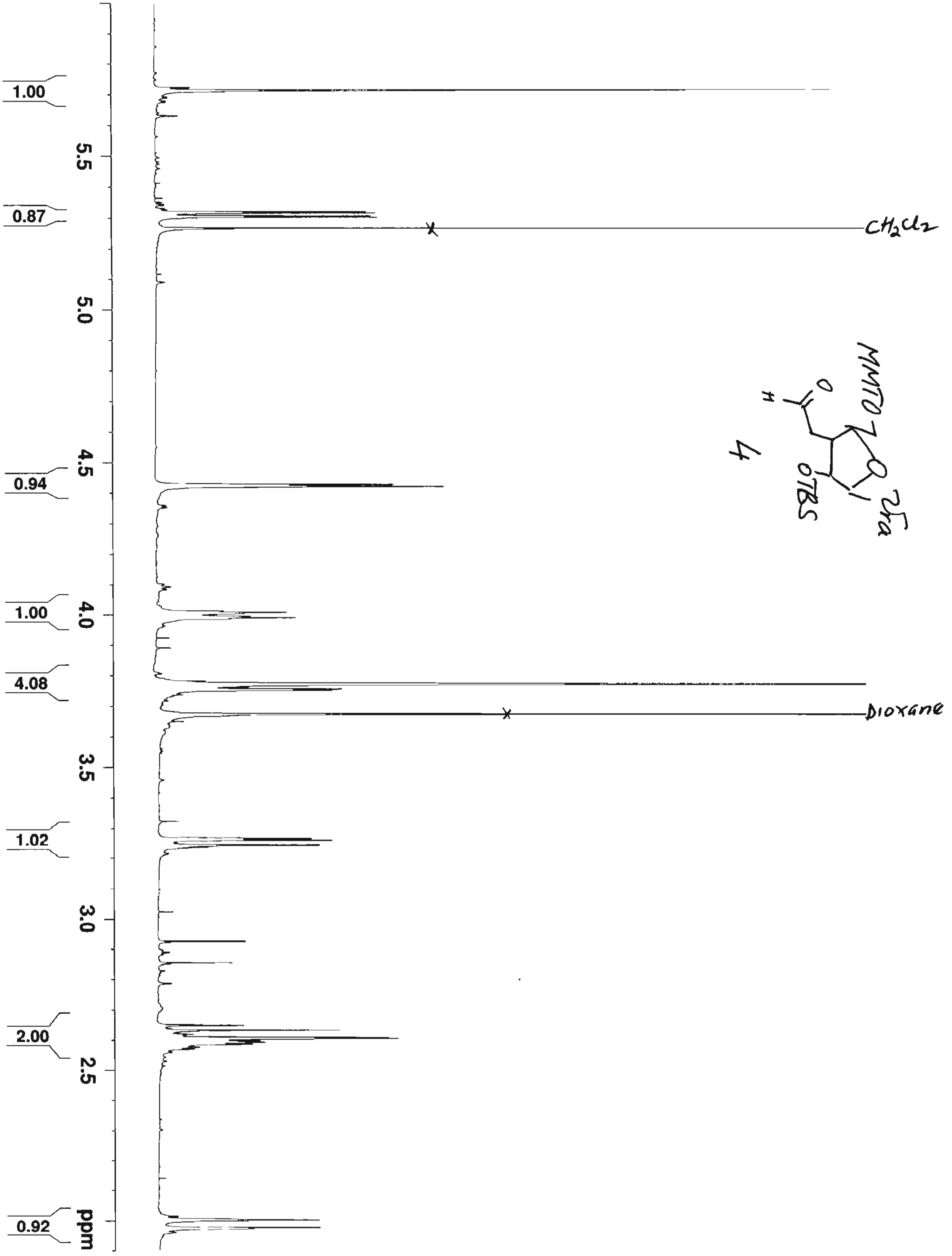
1.00
0.87
0.94
1.00
4.08
1.02
2.00
0.92
5.5
5.0
4.5
4.0
3.5
3.0
2.5
ppm
CH2Cl2
Dioxane
MMTO
OTBS
25a
4

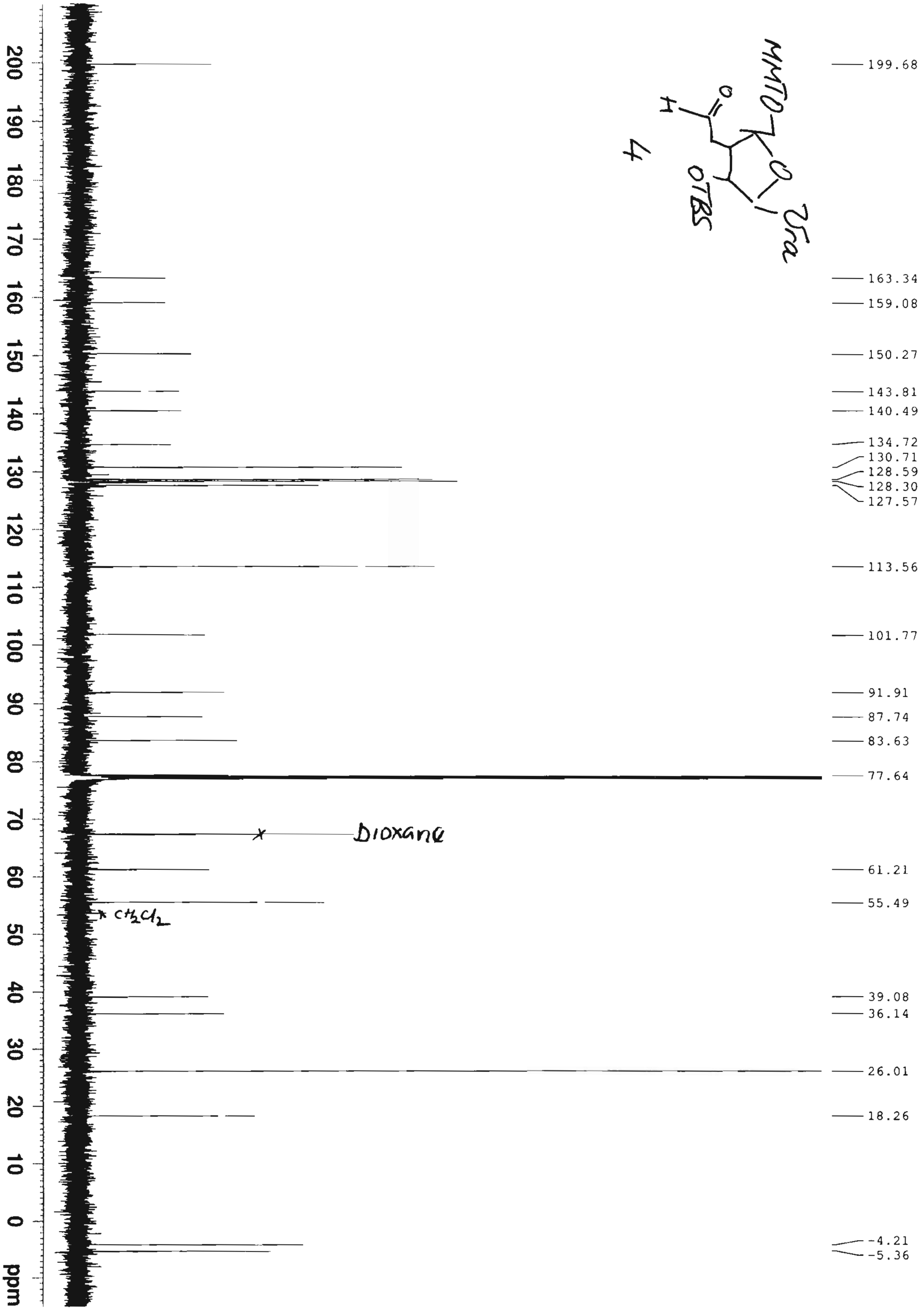
199.68
163.34
159.08
150.27
143.81
140.49
134.72
130.71
128.59
128.30
127.57
113.56
101.77
91.91
87.74
83.63
77.64
61.21
55.49
39.08
36.14
26.01
18.26
-4.21
-5.36
Dioxane
CH2Cl2
ppm
OTBS
4

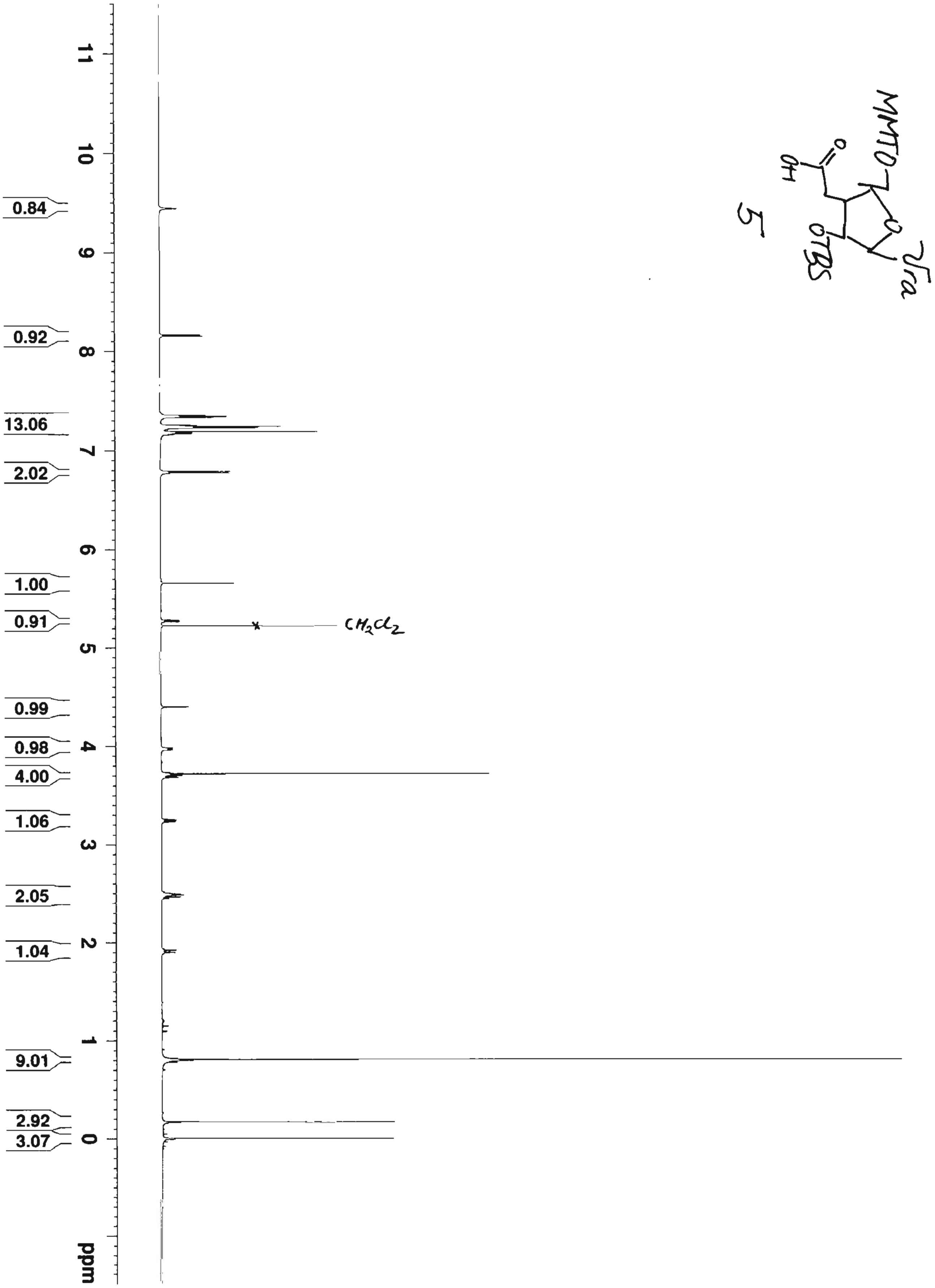

MMTO
Ura
OTBS
OH
5
CH2Cl2
0.84
0.92
13.06
2.02
1.00
0.91
0.99
0.98
4.00
1.06
2.05
1.04
9.01
2.92
3.07
ppm

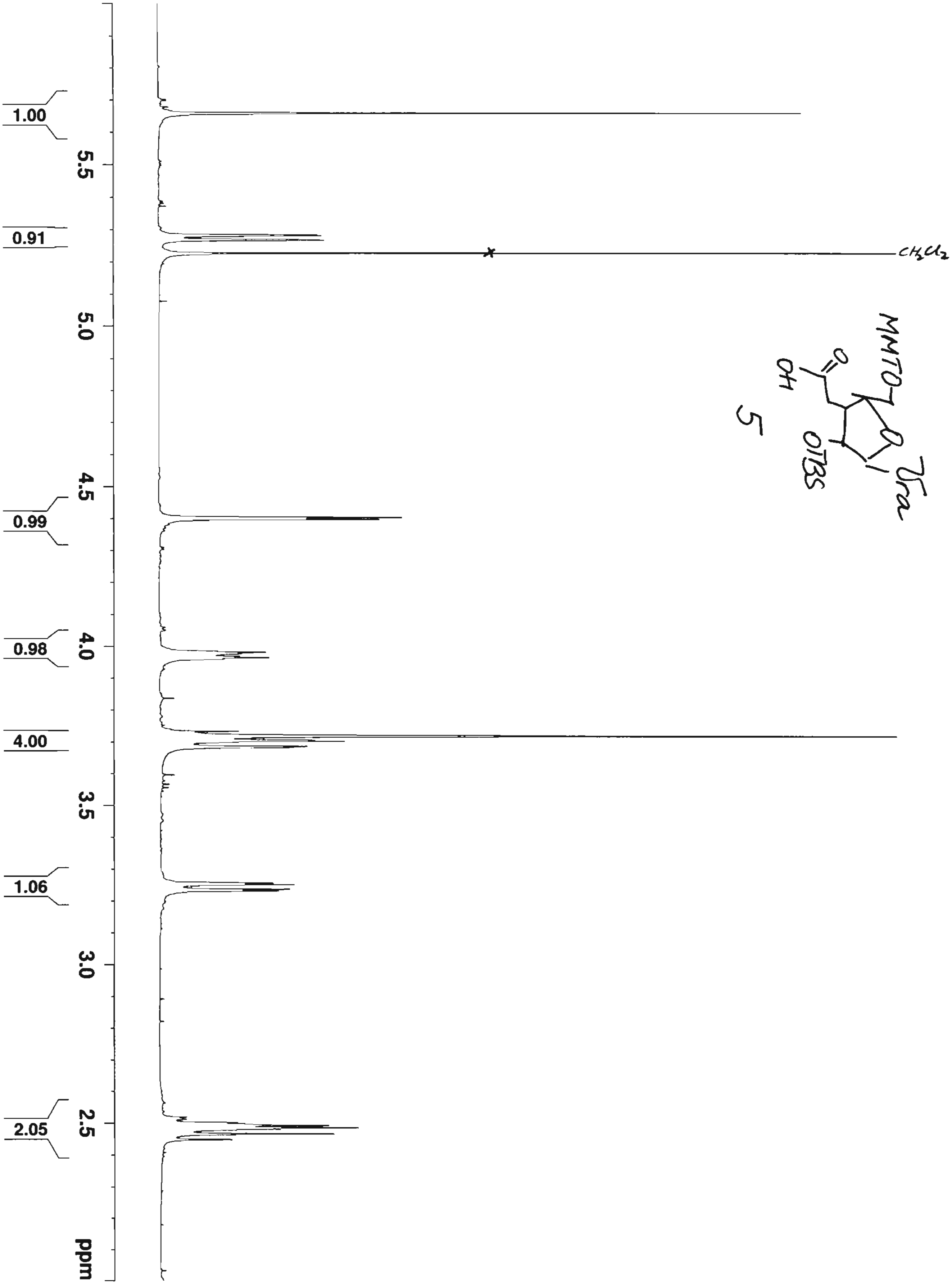

1.00
0.91
0.99
0.98
4.00
1.06
2.05
5.5
5.0
4.5
4.0
3.5
3.0
2.5
ppm
CH_2Cl_2
MMTO
OTBS
OH
Ura
5

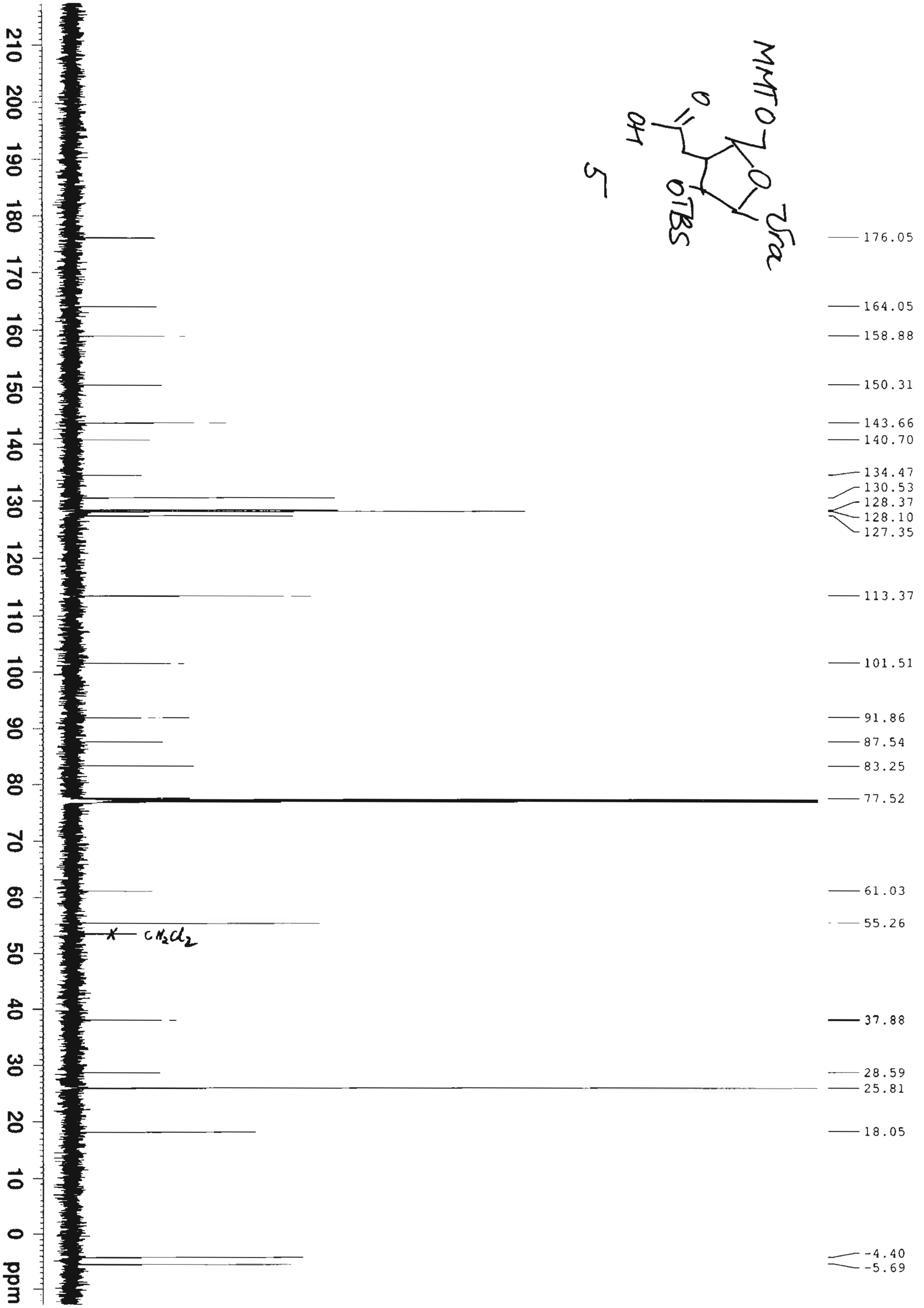
176.05
164.05
158.88
150.31
143.66
140.70
134.47
130.53
128.37
128.10
127.35
113.37
101.51
91.86
87.54
83.25
77.52
61.03
55.26
37.88
28.59
25.81
18.05
-4.40
-5.69
210 200 190 180 170 160 150 140 130 120 110 100 90 80 70 60 50 40 30 20 10 0 ppm
MMTO
Ura
OTBS
OH
5
CH2Cl2

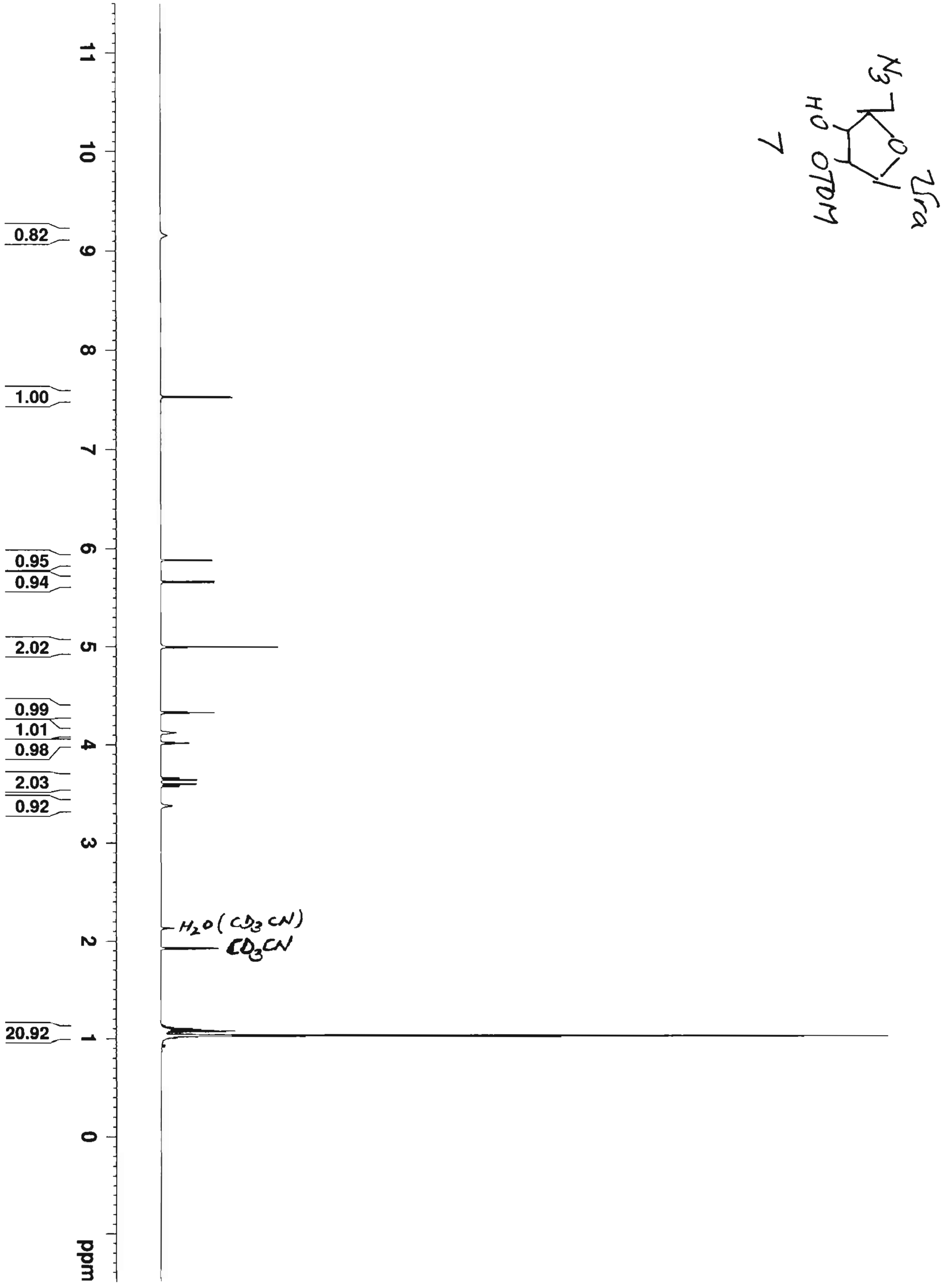
N3
HO
OTDM
Ura
7
0.82
1.00
0.95
0.94
2.02
0.99
1.01
0.98
2.03
0.92
20.92
H2O (CD3CN)
CD3CN
11
10
9
8
7
6
5
4
3
2
1
0
ppm

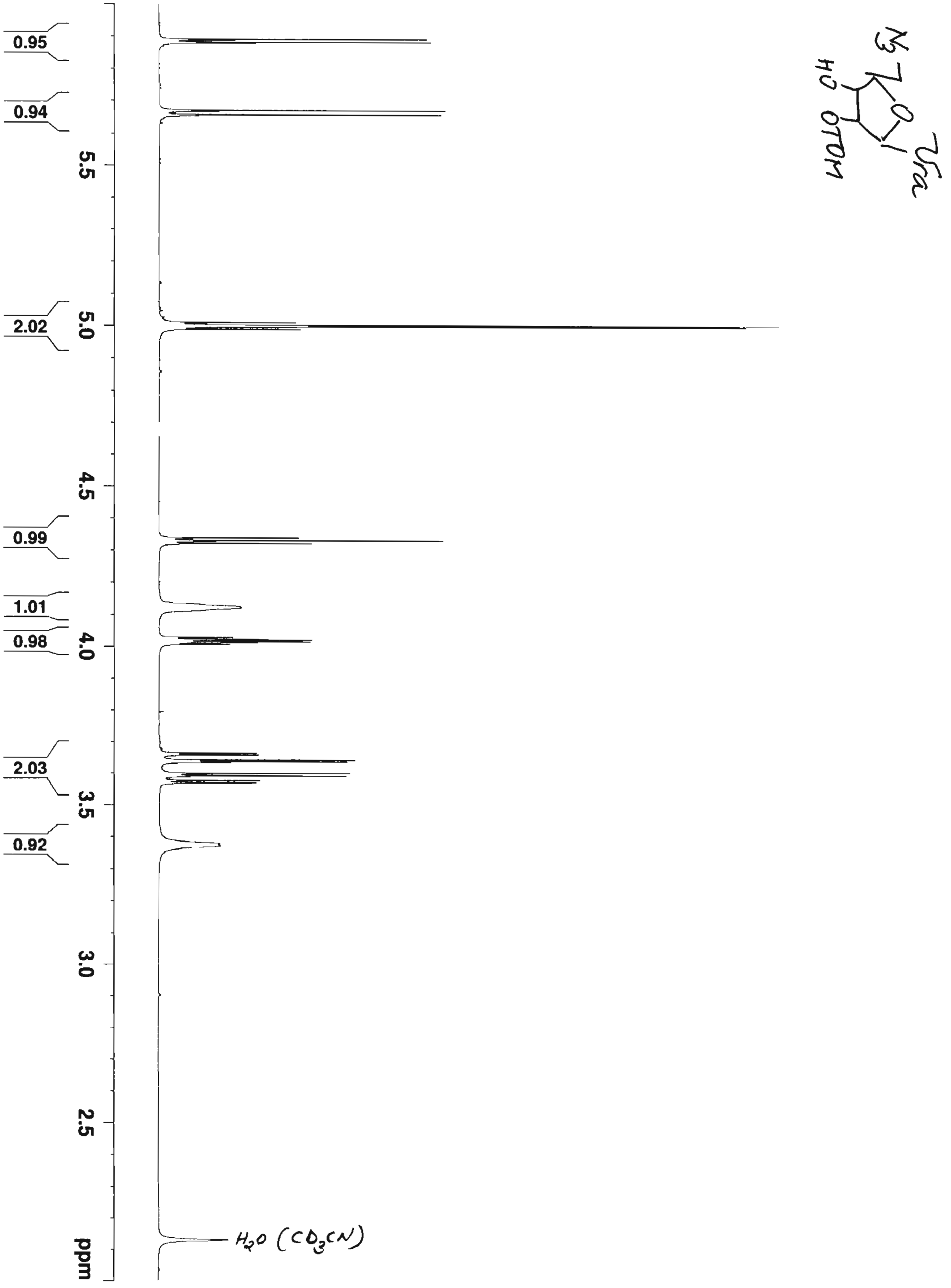
0.95
0.94
2.02
0.99
1.01
0.98
2.03
0.92
5.5
5.0
4.5
4.0
3.5
3.0
2.5
ppm
H2O (CD3CN)

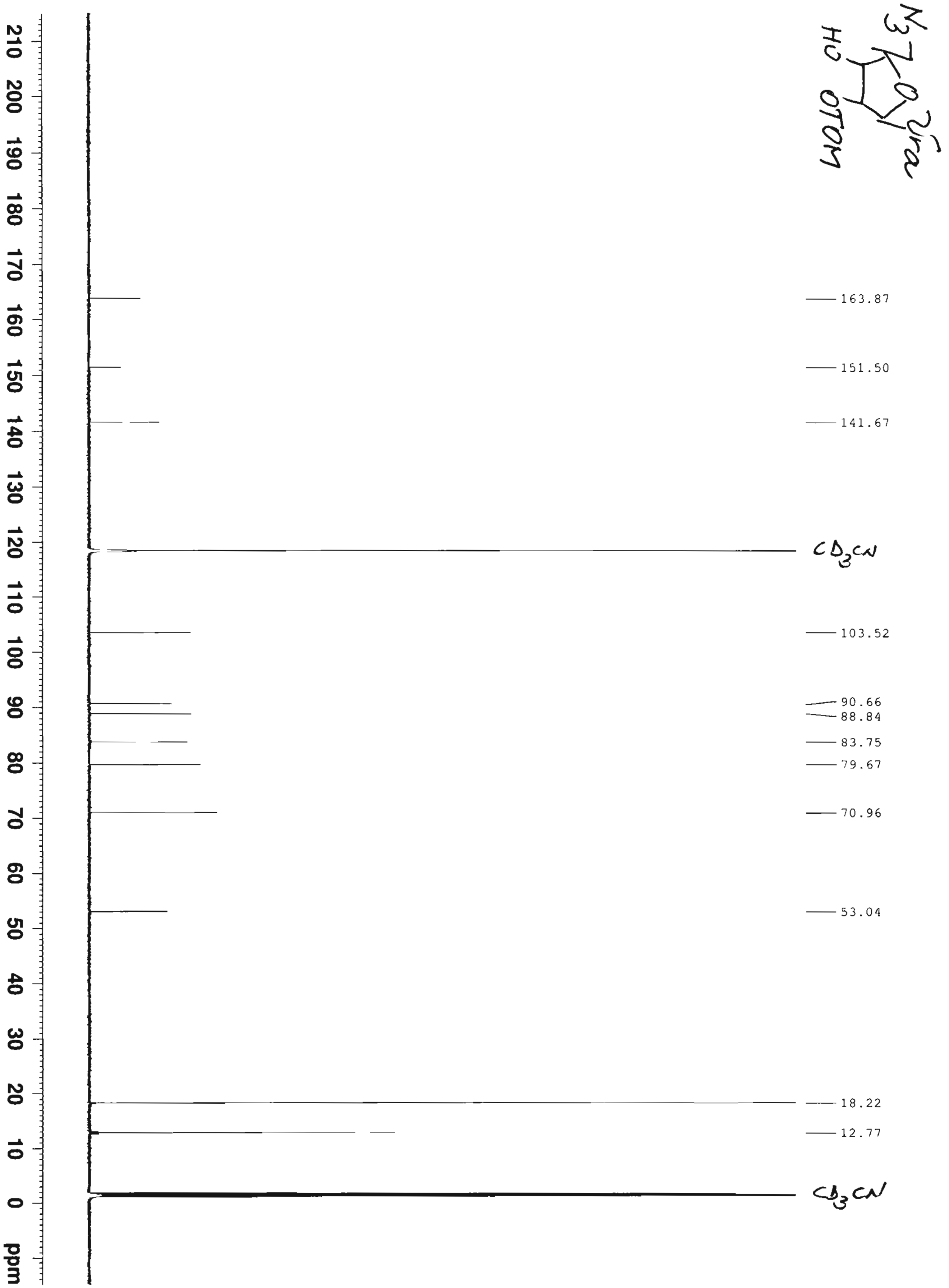

N3
O
Ura
HO
OTOM
163.87
151.50
141.67
CD3CN
103.52
90.66
88.84
83.75
79.67
70.96
53.04
18.22
12.77
CD3CN
210
200
190
180
170
160
150
140
130
120
110
100
90
80
70
60
50
40
30
20
10
0
ppm

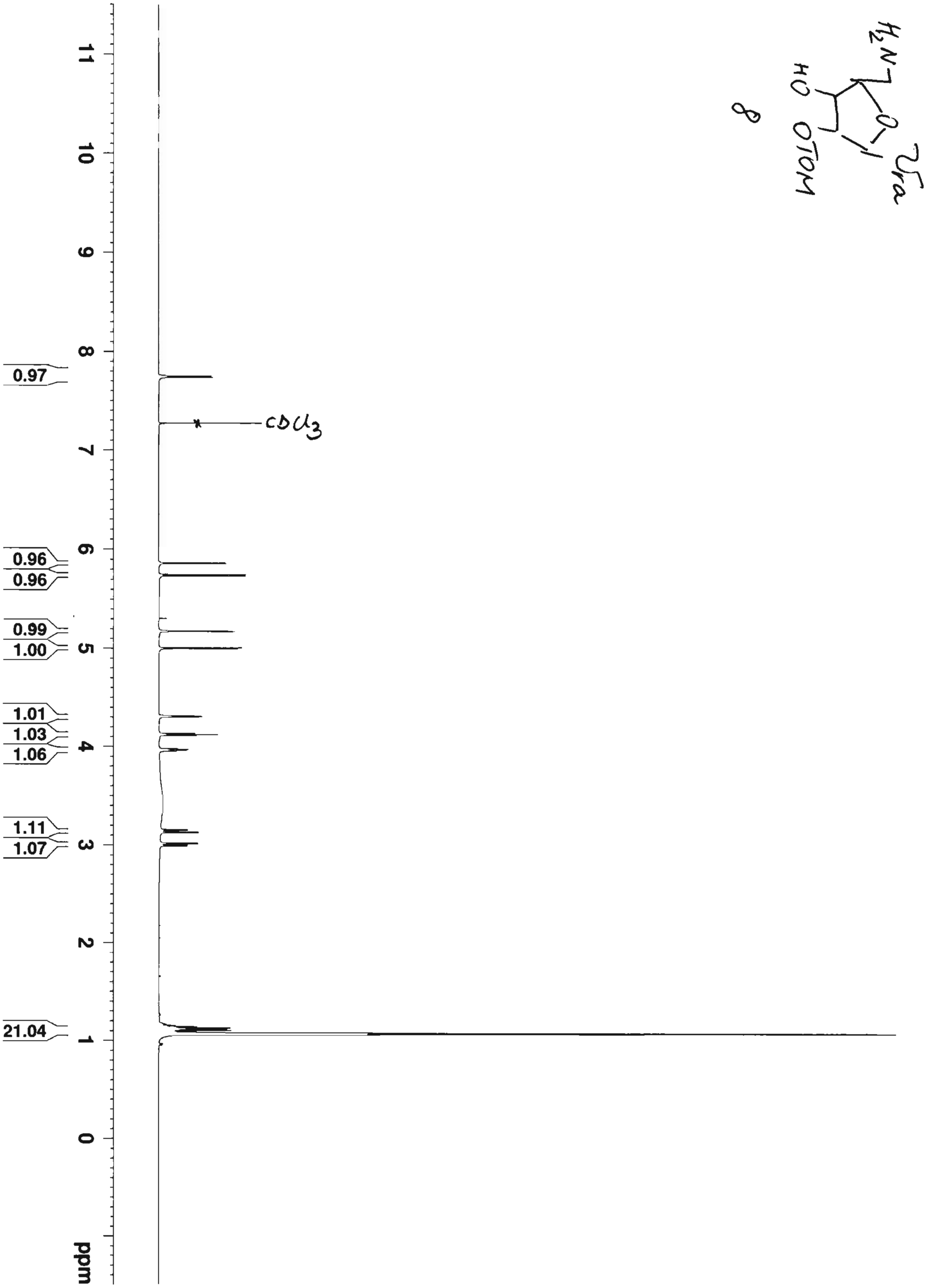

ppm
0.97
0.96
0.96
0.99
1.00
1.01
1.03
1.06
1.11
1.07
21.04
CDCl3
8

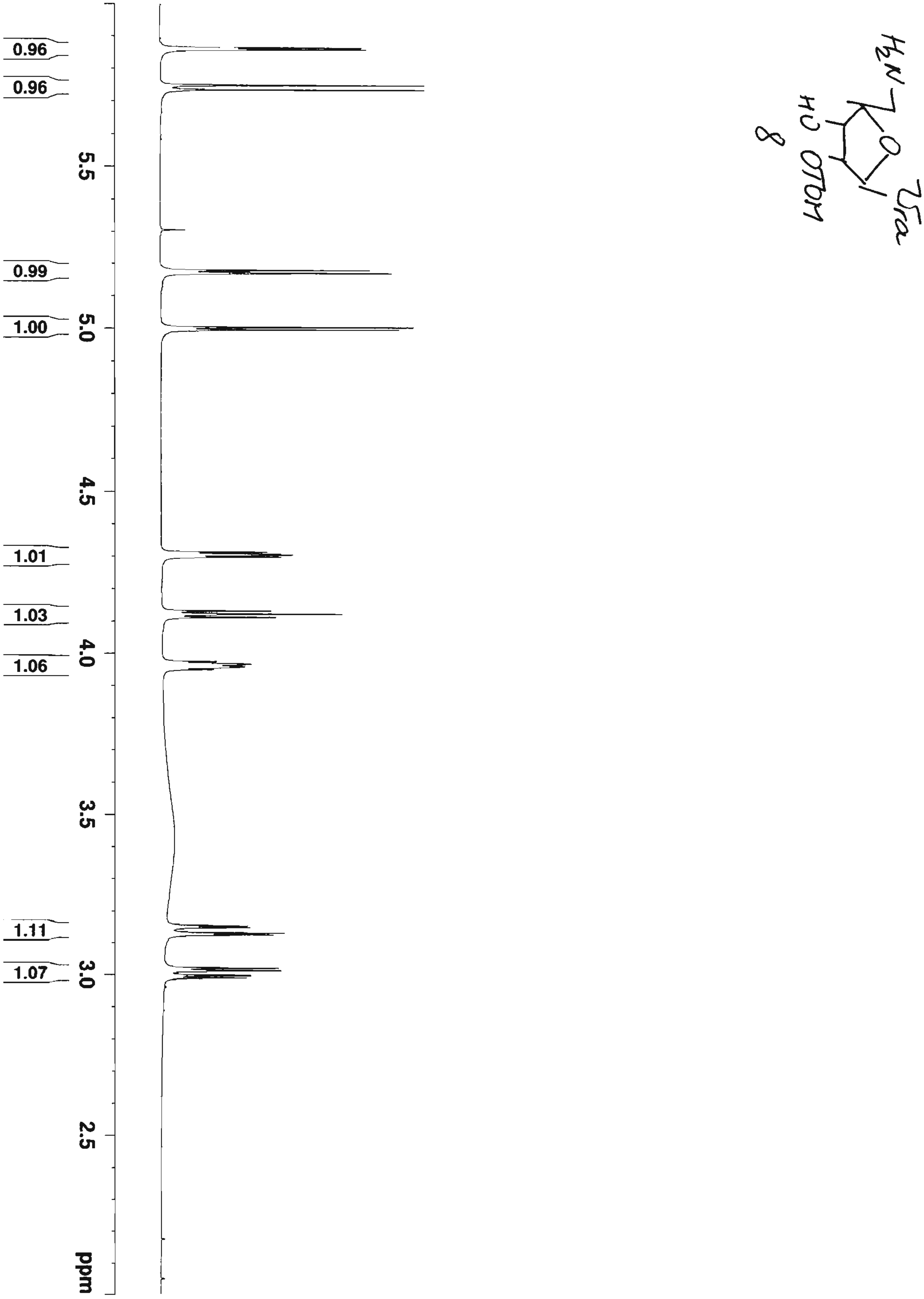

0.96
0.96
0.99
1.00
1.01
1.03
1.06
1.11
1.07
5.5
5.0
4.5
4.0
3.5
3.0
2.5
ppm

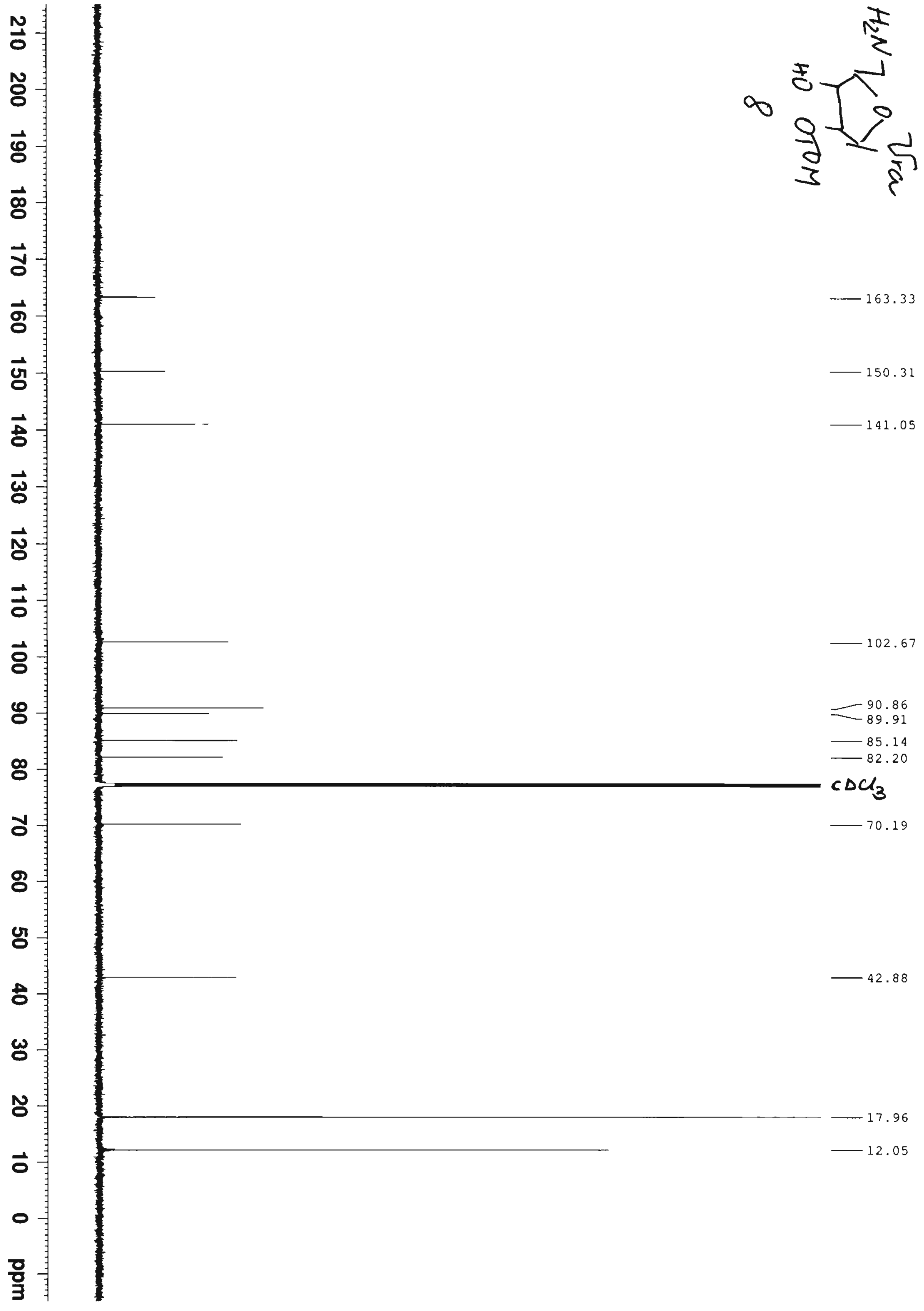

163.33
150.31
141.05
102.67
90.86
89.91
85.14
82.20
CDCl3
70.19
42.88
17.96
12.05
210 200 190 180 170 160 150 140 130 120 110 100 90 80 70 60 50 40 30 20 10 0 ppm
8

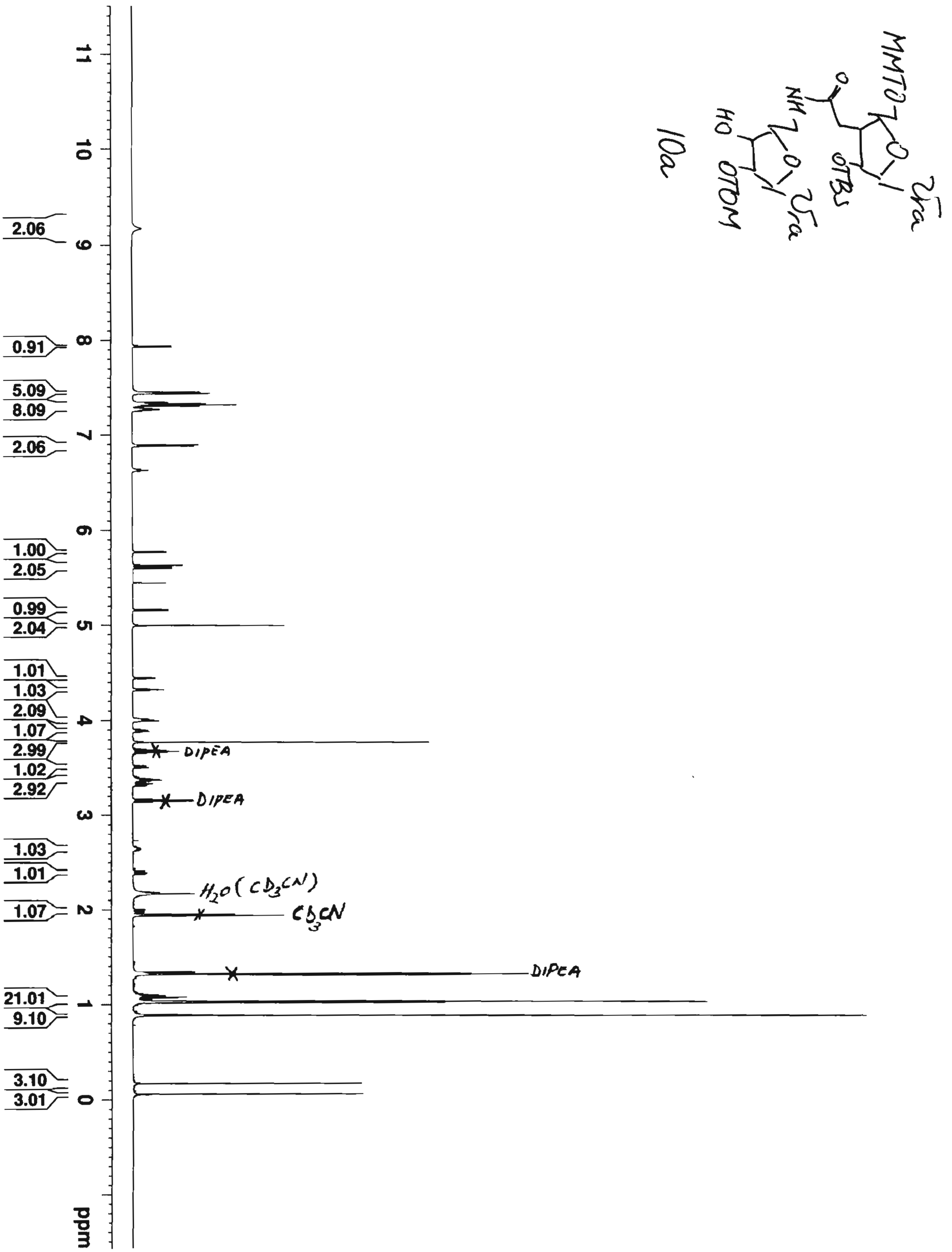

10a
MMTrO
OTBS
OTOM
HO
NH
Ura
DIPEA
DIPEA
H2O (CD3CN)
CD3CN
DIPEA
ppm
2.06
0.91
5.09
8.09
2.06
1.00
2.05
0.99
2.04
1.01
1.03
2.09
1.07
2.99
1.02
2.92
1.03
1.01
1.07
21.01
9.10
3.10
3.01

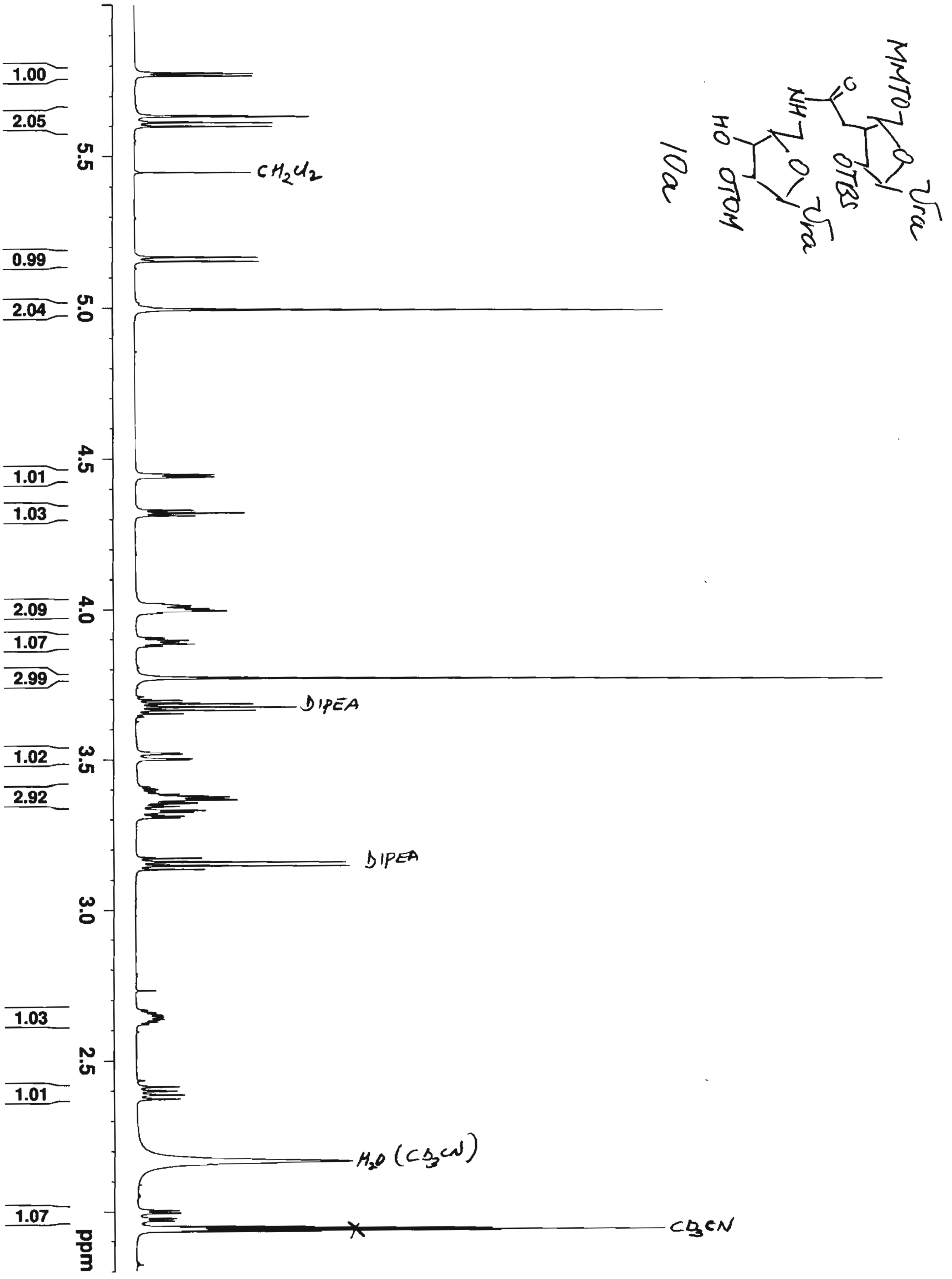

1.00
2.05
0.99
2.04
1.01
1.03
2.09
1.07
2.99
1.02
2.92
1.03
1.01
1.07
5.5
5.0
4.5
4.0
3.5
3.0
2.5
ppm
CH2Cl2
DIPEA
DIPEA
H2O (CD3CN)
CD3CN
MMTO
Ura
OTBS
NH
HO
OTOM
10a

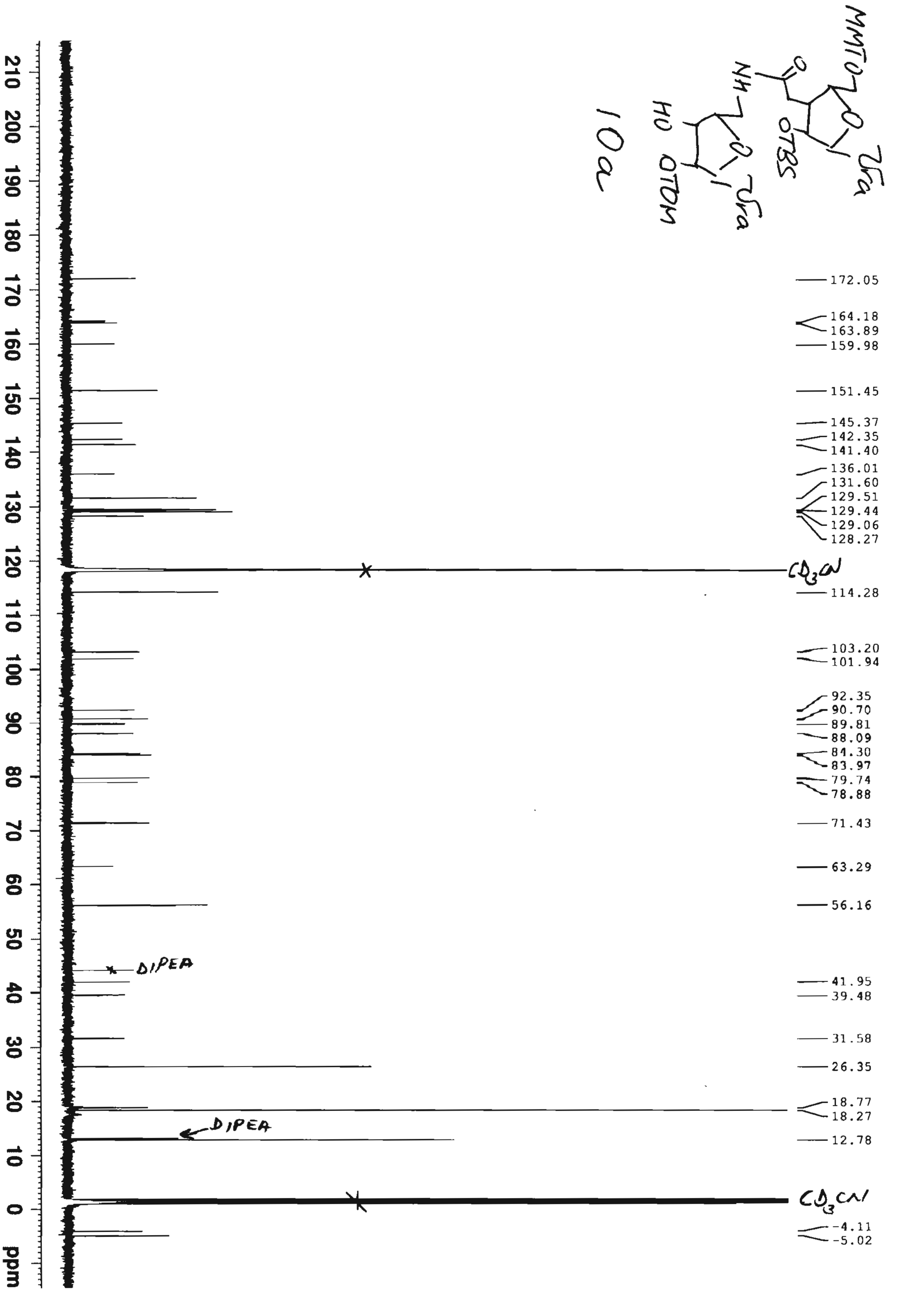
MMTO
Ura
OTBS
NH
Ura
HO OTOM
10a
172.05
164.18
163.89
159.98
151.45
145.37
142.35
141.40
136.01
131.60
129.51
129.44
129.06
128.27
CD3CN
114.28
103.20
101.94
92.35
90.70
89.81
88.09
84.30
83.97
79.74
78.88
71.43
63.29
56.16
DIPEA
41.95
39.48
31.58
26.35
18.77
18.27
DIPEA
12.78
CD3CN
-4.11
-5.02
210 200 190 180 170 160 150 140 130 120 110 100 90 80 70 60 50 40 30 20 10 0 ppm

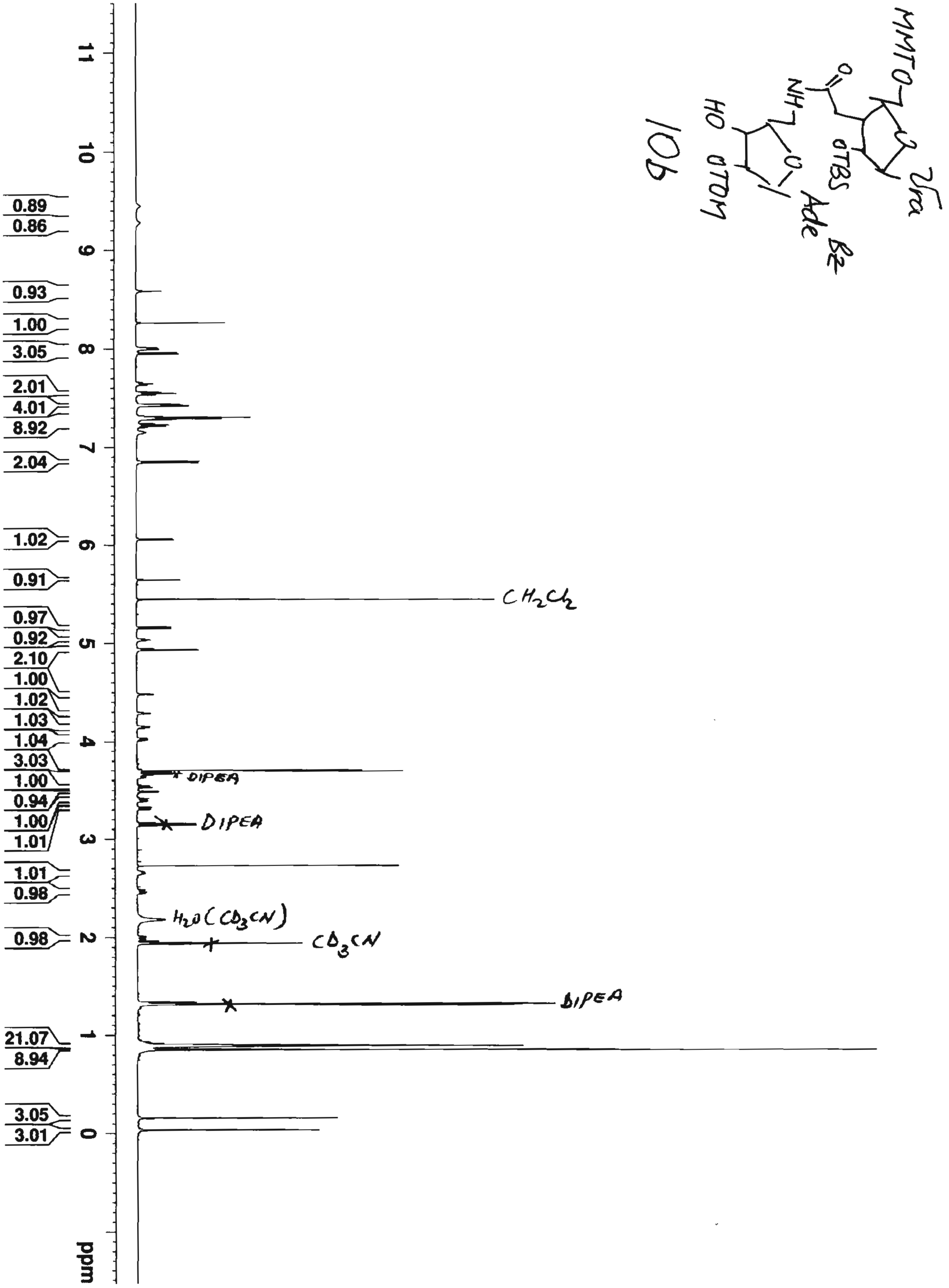
10b
CH2Cl2
DIPEA
DIPEA
H2O (CD3CN)
CD3CN
DIPEA
ppm

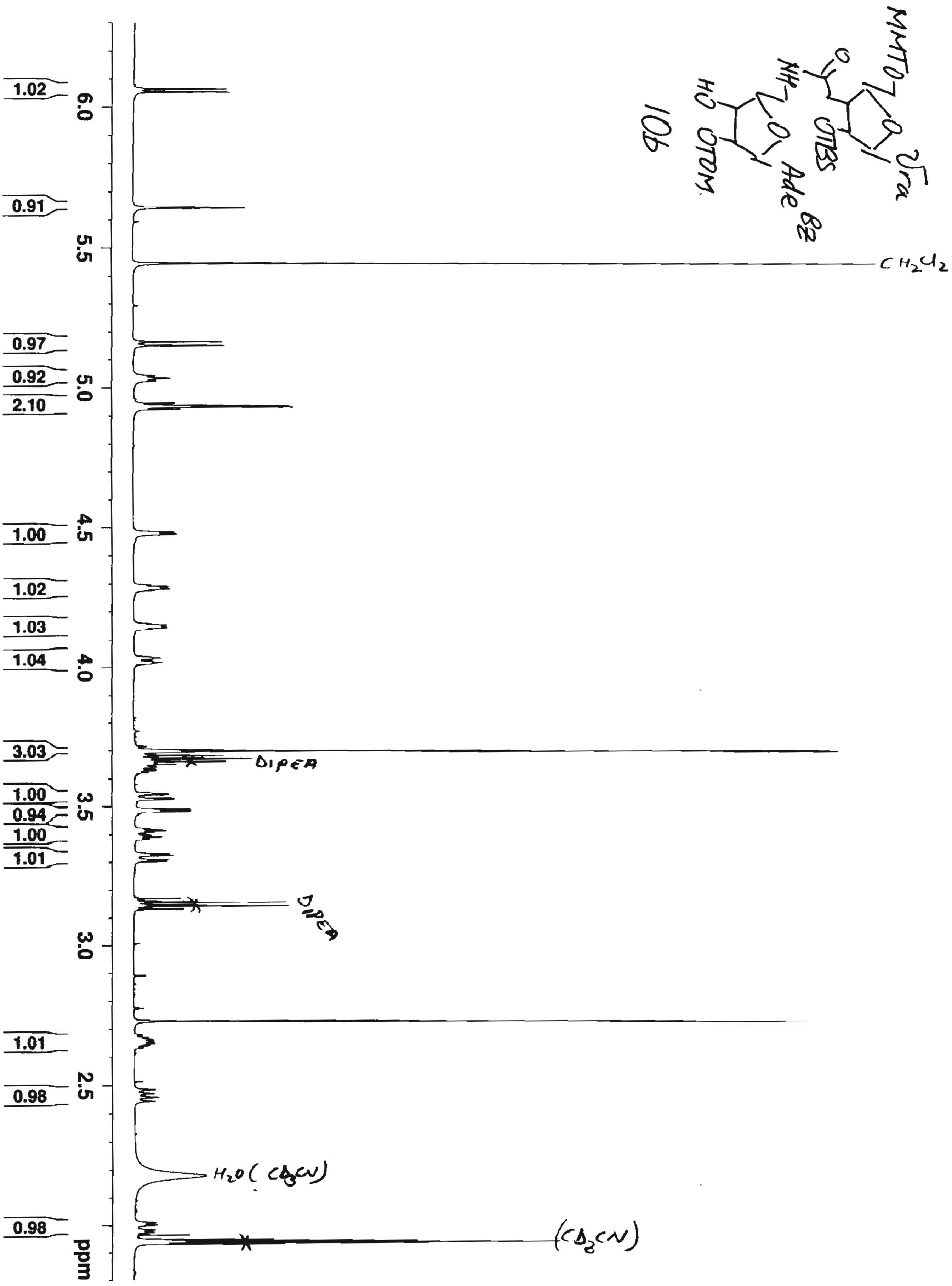

ppm
6.0
5.5
5.0
4.5
4.0
3.5
3.0
2.5
1.02
0.91
0.97
0.92
2.10
1.00
1.02
1.03
1.04
3.03
1.00
0.94
1.00
1.01
1.01
0.98
0.98
10b
DIPEA
DIPEA

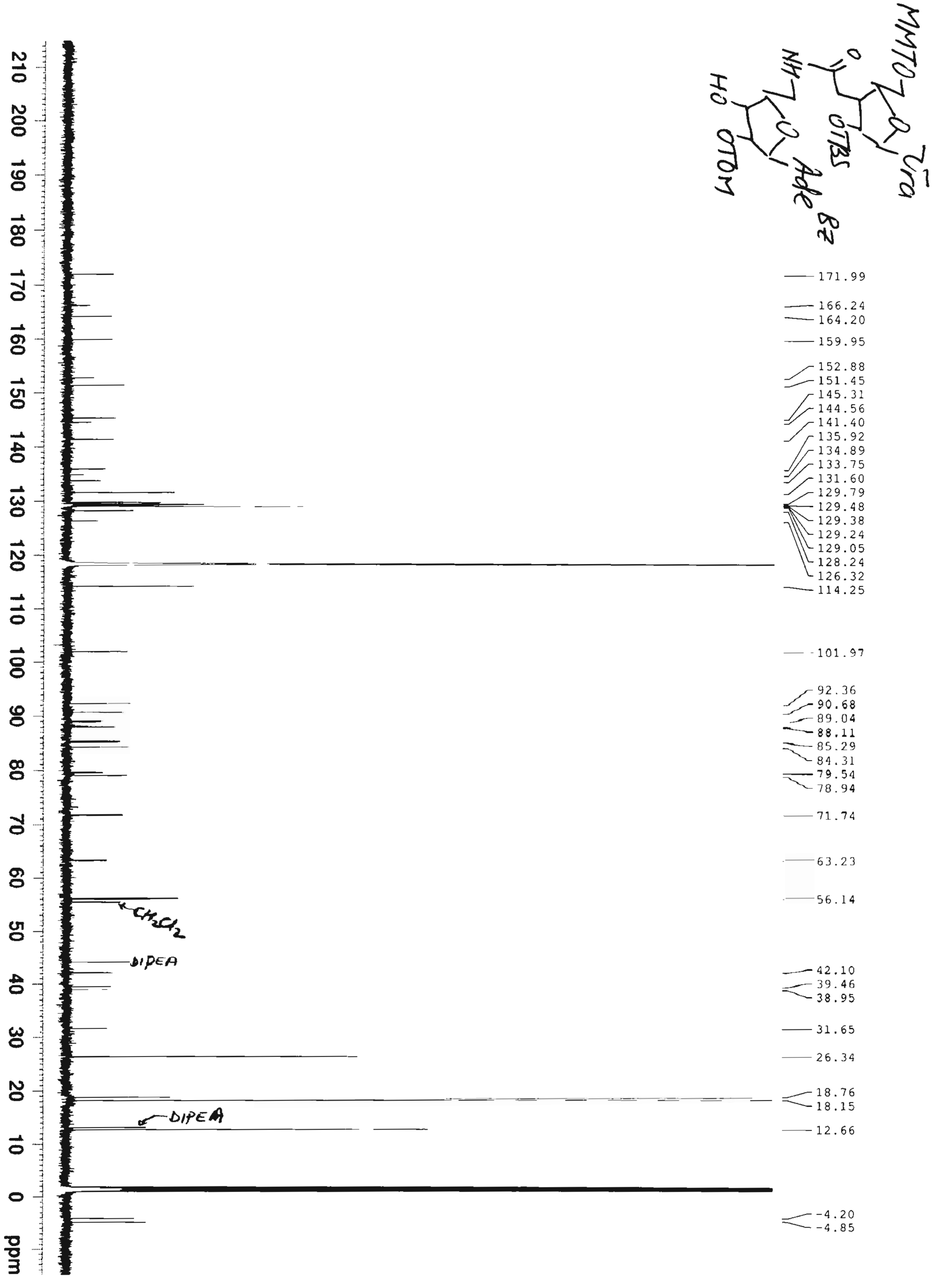

MMTO
Ura
OTBS
NH
Ade Bz
HO
OTOM
171.99
166.24
164.20
159.95
152.88
151.45
145.31
144.56
141.40
135.92
134.89
133.75
131.60
129.79
129.48
129.38
129.24
129.05
128.24
126.32
114.25
101.97
92.36
90.68
89.04
88.11
85.29
84.31
79.54
78.94
71.74
63.23
56.14
42.10
39.46
38.95
31.65
26.34
18.76
18.15
12.66
-4.20
-4.85
CH2Cl2
DIPEA
DIPEA
210
200
190
180
170
160
150
140
130
120
110
100
90
80
70
60
50
40
30
20
10
0
ppm

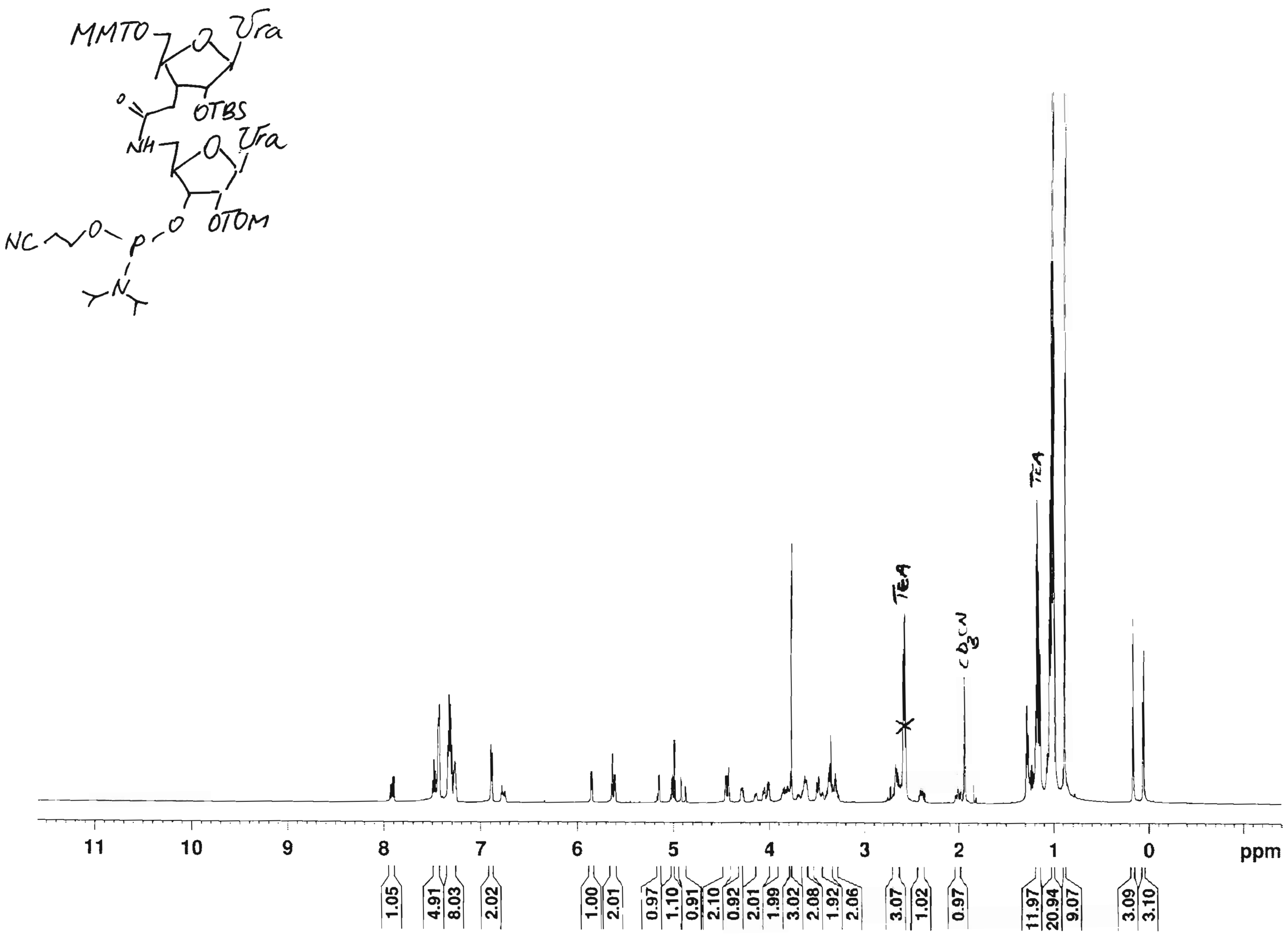
MMTO
Ura
OTBS
NH
Ura
OTOM
NC
P
N
TEA
CD_3CN
TEA
11
10
9
8
7
6
5
4
3
2
1
0
ppm
1.05
4.91
8.03
2.02
1.00
2.01
0.97
1.10
0.91
2.10
0.92
2.01
1.99
3.02
2.08
1.92
2.06
3.07
1.02
0.97
11.97
20.94
9.07
3.09
3.10

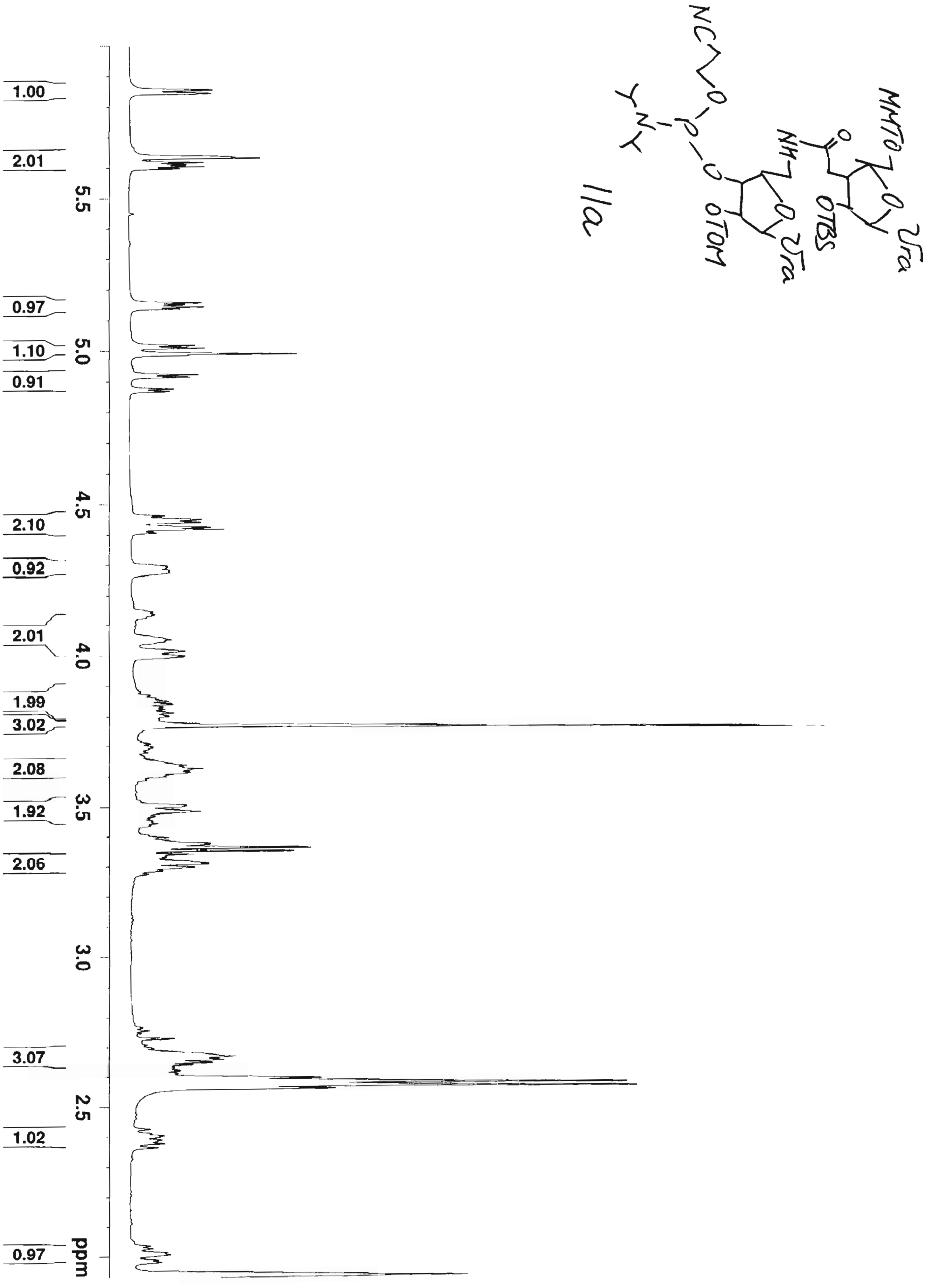
11a
1.00
2.01
5.5
0.97
1.10
5.0
0.91
4.5
2.10
0.92
2.01
4.0
1.99
3.02
2.08
3.5
1.92
2.06
3.0
3.07
2.5
1.02
0.97
ppm

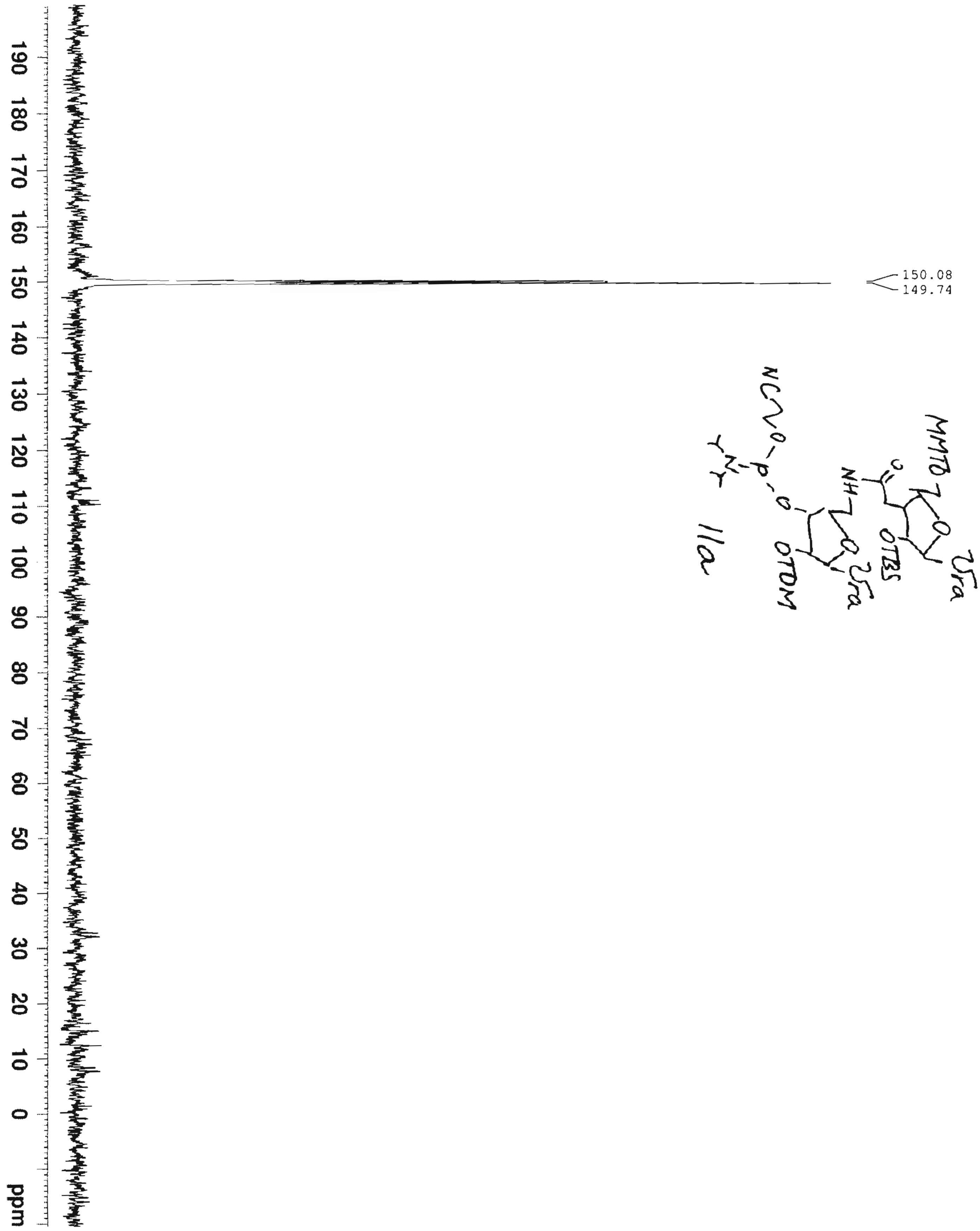
150.08
149.74
190 180 170 160 150 140 130 120 110 100 90 80 70 60 50 40 30 20 10 0 ppm
MMTO
Ura
OTBS
NH
OTDM
NC
11a

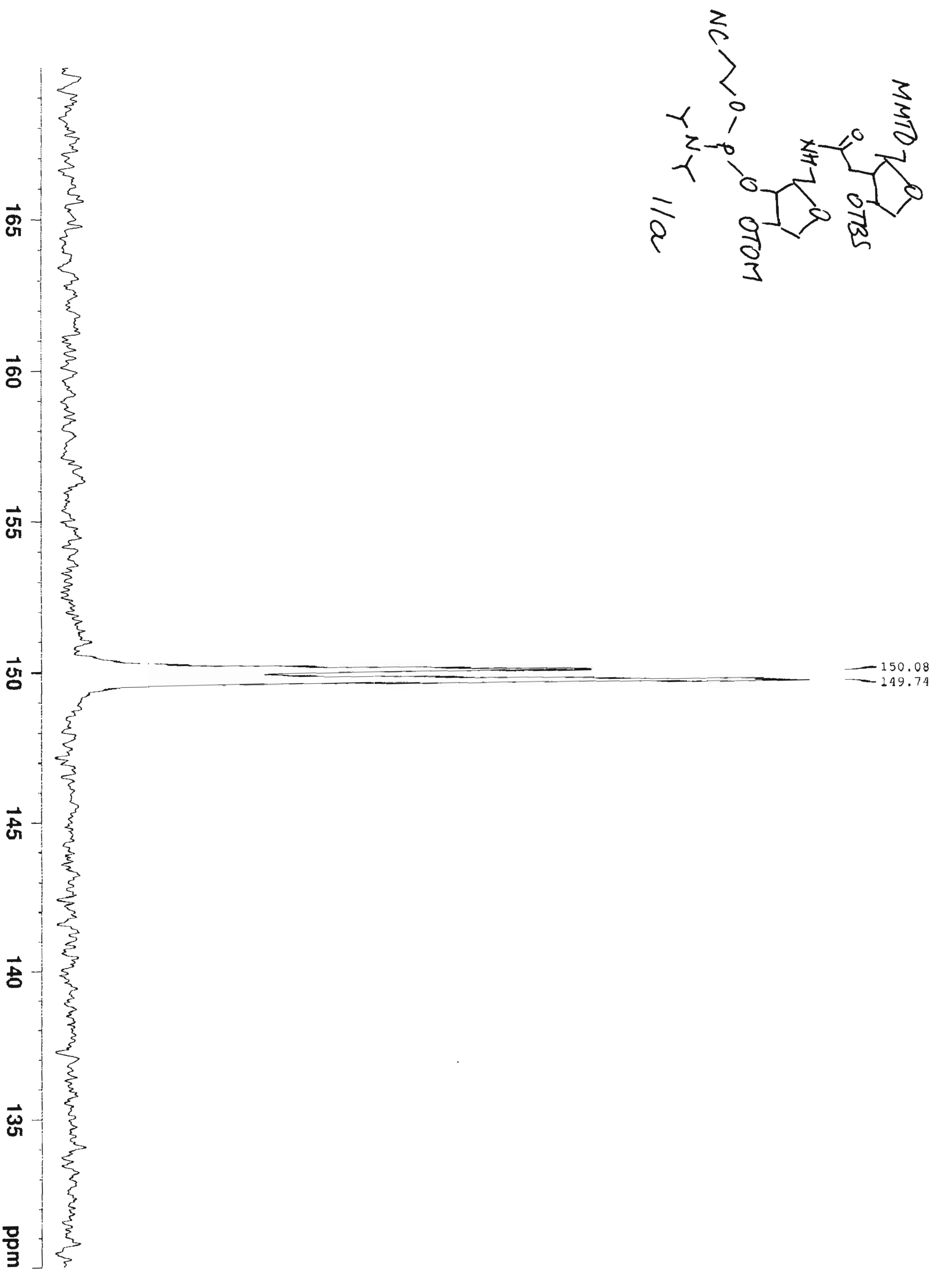
150.08
149.74
165
160
155
150
145
140
135
ppm

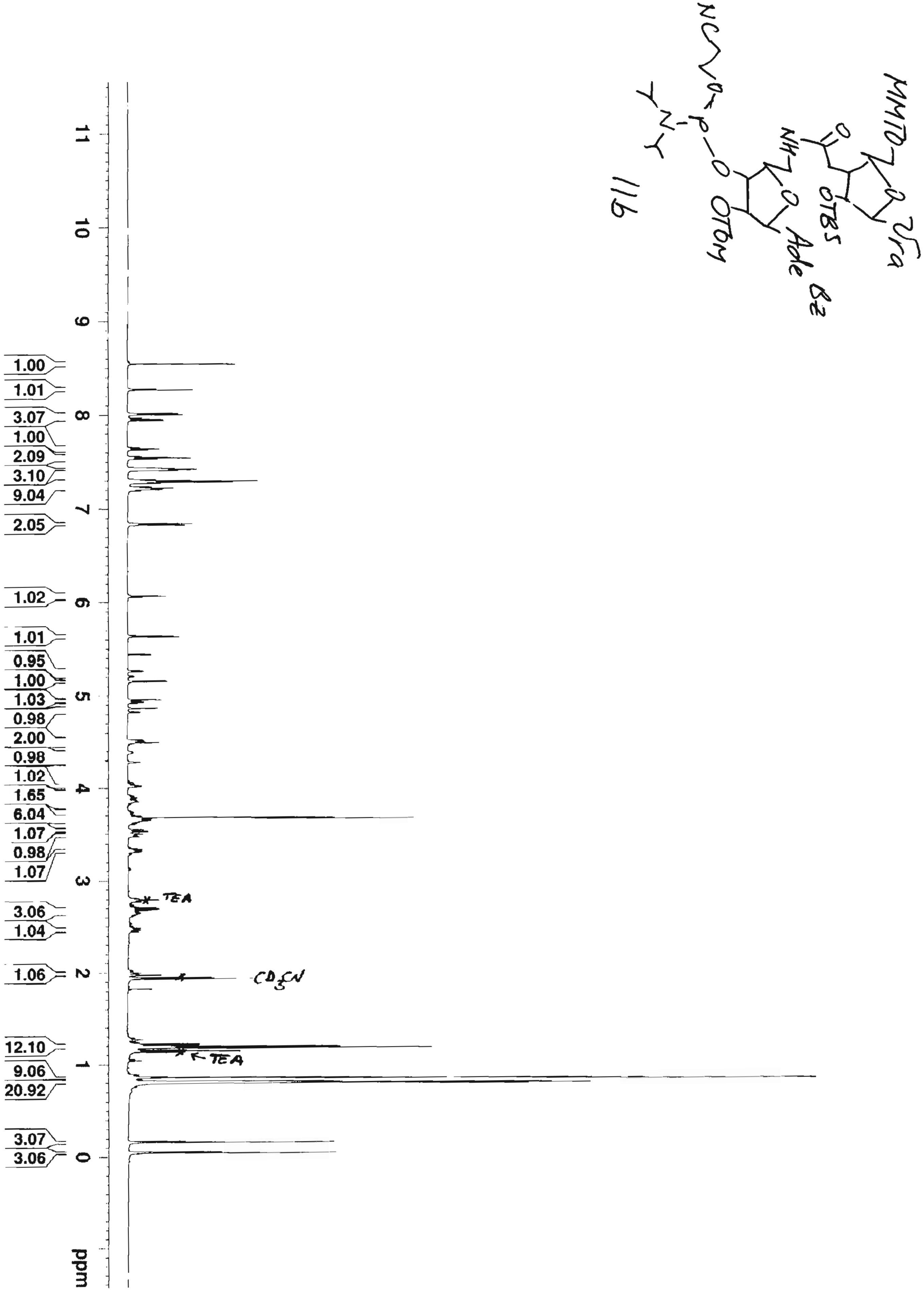

ppm
11
10
9
8
7
6
5
4
3
2
1
0
1.00
1.01
3.07
1.00
2.09
3.10
9.04
2.05
1.02
1.01
0.95
1.00
1.03
0.98
2.00
0.98
1.02
1.65
6.04
1.07
0.98
1.07
3.06
1.04
1.06
12.10
9.06
20.92
3.07
3.06
TEA
TEA

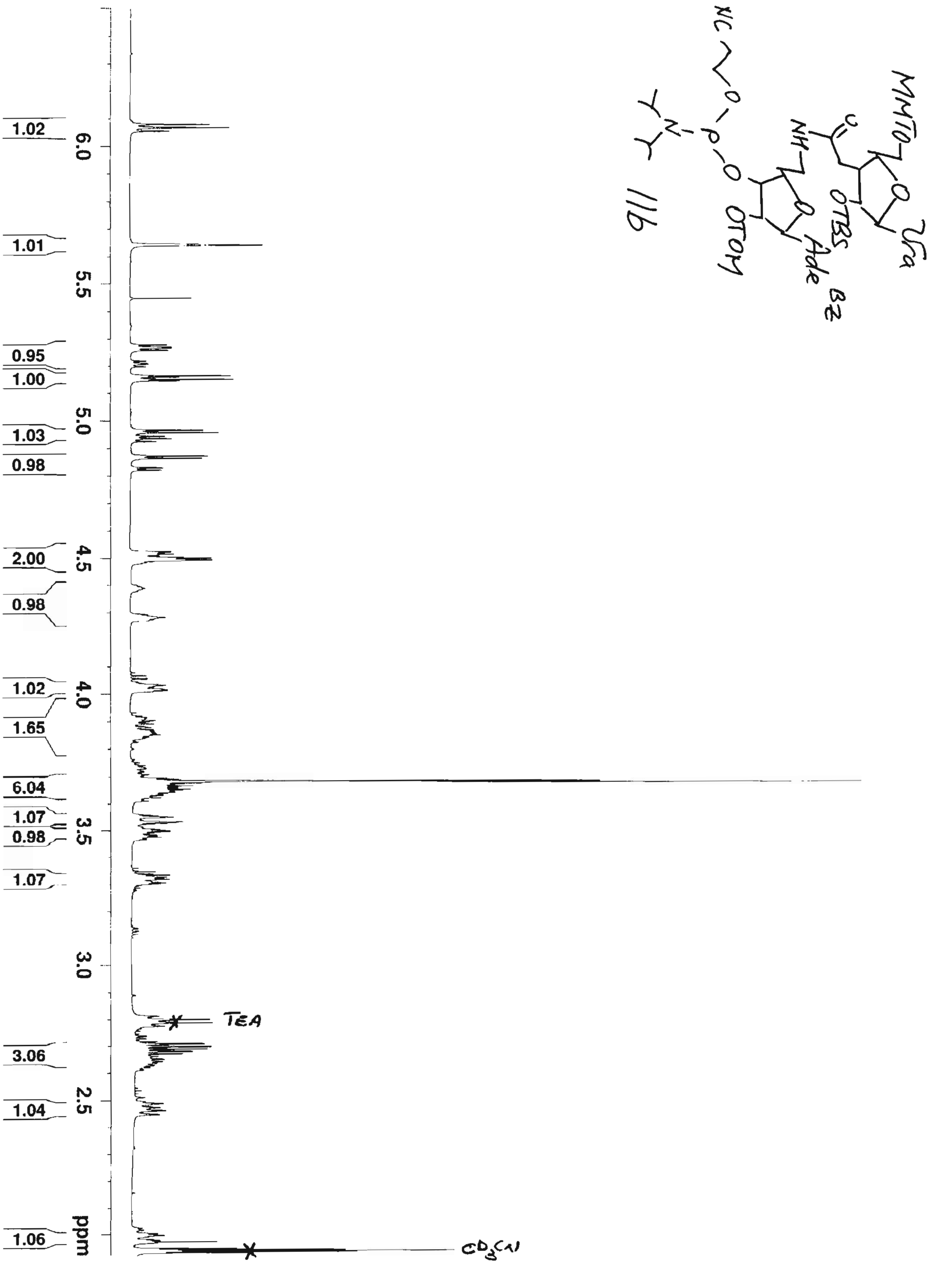
MMTO
Ura
OTBS
Ade Bz
NH
O
NC
P
N
OTOM
11b
1.02
1.01
0.95
1.00
1.03
0.98
2.00
0.98
1.02
1.65
6.04
1.07
0.98
1.07
3.06
1.04
1.06
6.0
5.5
5.0
4.5
4.0
3.5
3.0
2.5
ppm
TEA

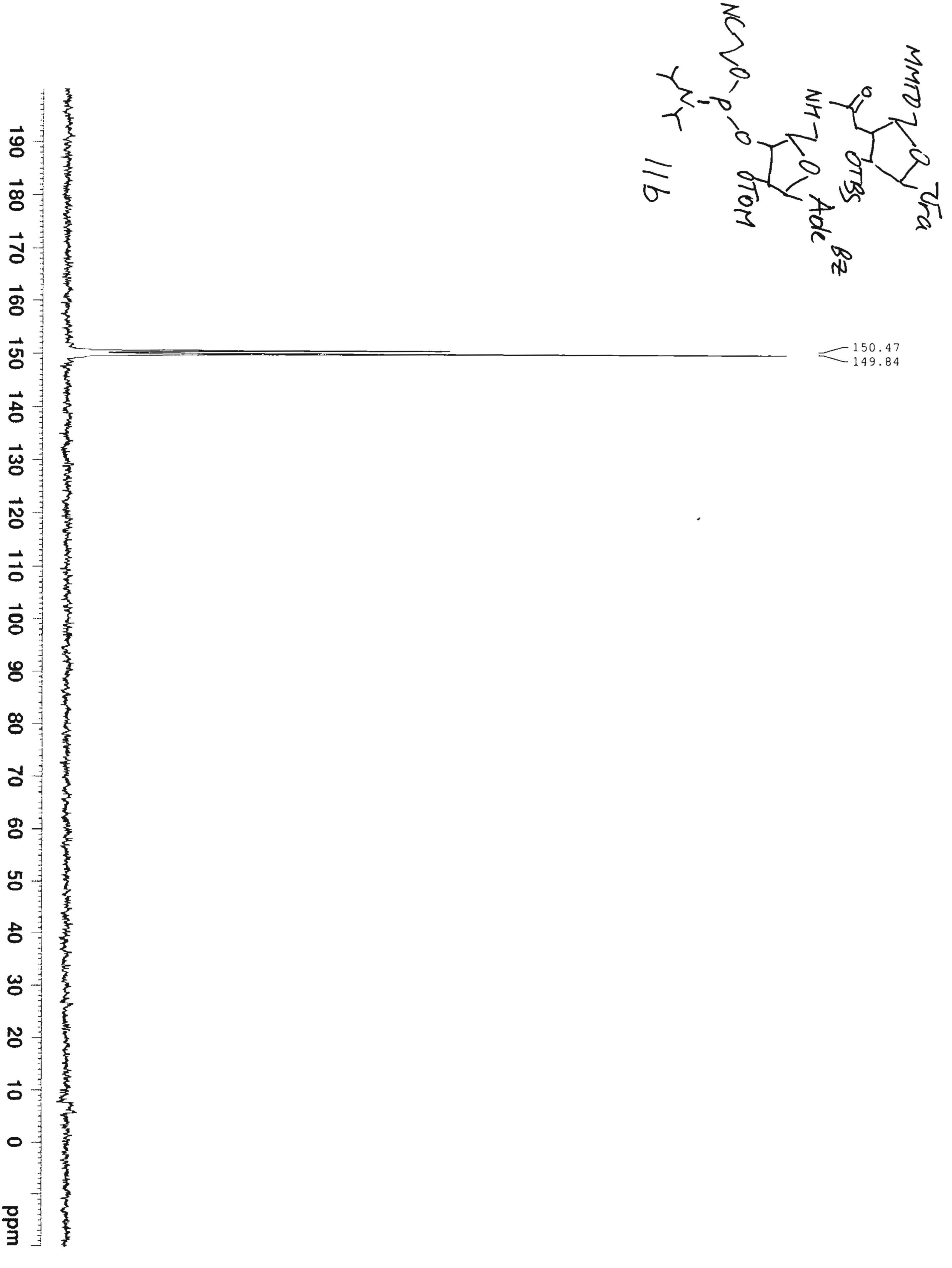
150.47
149.84
190 180 170 160 150 140 130 120 110 100 90 80 70 60 50 40 30 20 10 0
ppm

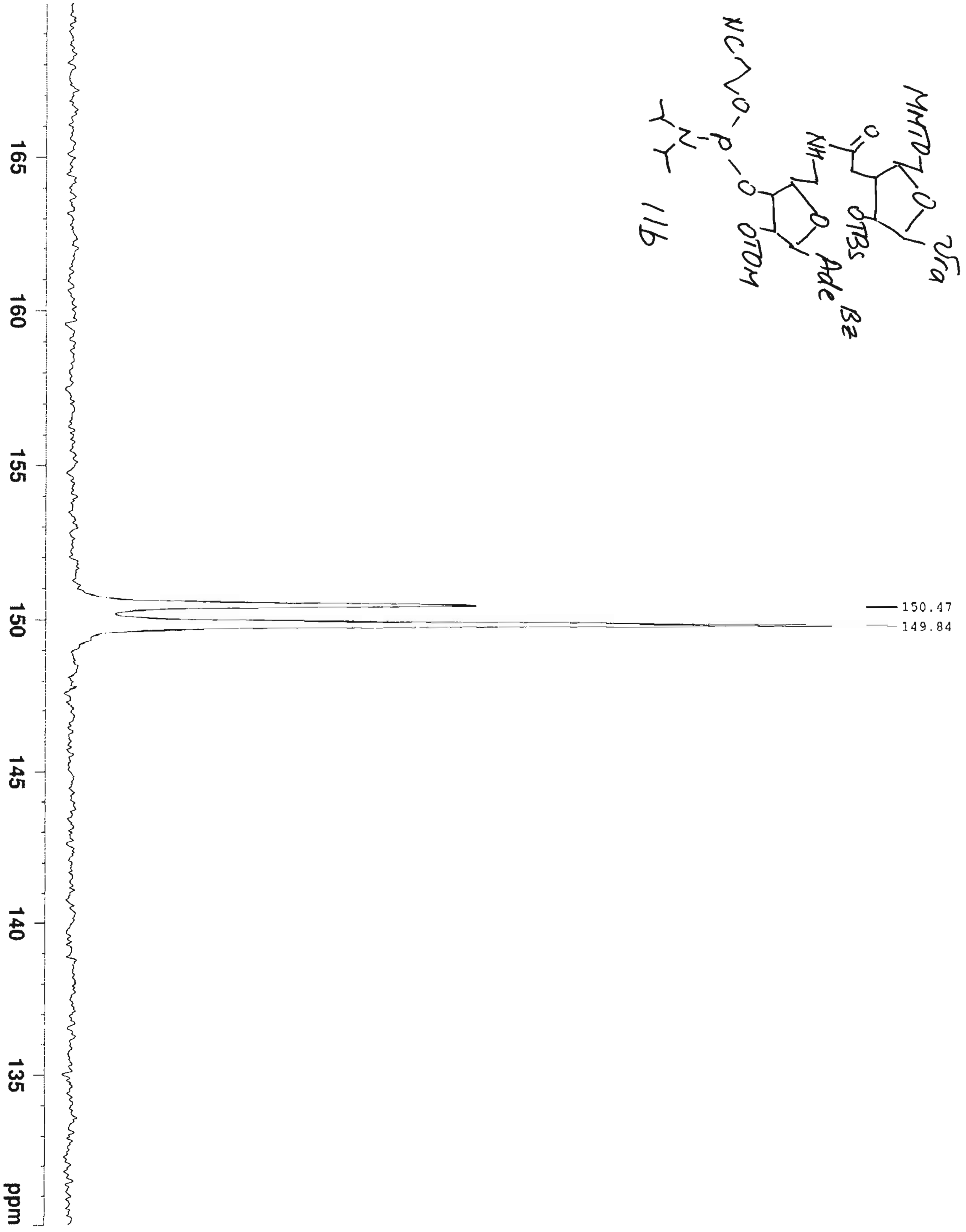
11b
150.47
149.84
165
160
155
150
145
140
135
ppm
